# Pathogenic variants in *BORCS5* cause a spectrum of neurodevelopmental and neurodegenerative disorders with lysosomal dysfunction

**DOI:** 10.1172/JCI195336

**Published:** 2026-04-21

**Authors:** Niccolò E. Mencacci, Georgia Minakaki, Reza Maroofian, Raffaella De Pace, Adeline Paimboeuf, Tiago Branco Fonseca, Tatiana Abramova, Patrick Shannon, David Chitayat, Francesca Magrinelli, Wesley J. Peng, Diptaman Chatterjee, Sara H. Eldessouky, Julia Baptista, Tamas Marton, Julie Vogt, Juan Dario Ortigoza-Escobar, Loreto Martorell, Marta Gómez-Chiari, Ingrid M. Wentzensen, Erik-Jan Kamsteeg, Maha S. Zaki, Annarita Scardamaglia, Giovanni Zifarelli, Zuhair Nasser Al-Hassnan, Elka Miller, Shiri Shinar, Lova S. Matsa, Sri Hari Chandan Appikonda, Ghada A. Otaify, Khalid Al-Thihli, Almundher Al-Maawali, Michael Schwake, Mariasavina Severino, Henry Houlden, Shunmoogum A. Patten, Juan S. Bonifacino, Kailash P. Bhatia, Dimitri Krainc

**Affiliations:** 1 Davee Department of Neurology, Northwestern University Feinberg School of Medicine, Chicago, Illinois, USA.; 2 Department of Neuromuscular Disorders, UCL Queen Square Institute of Neurology, University College London, London, United Kingdom.; 3 Division of Neuroscience and Cellular Structure, *Eunice Kennedy Shriver* National Institute of Child Health and Human Development, NIH, Bethesda, Maryland, USA.; 4 Institut National de la Recherche Scientifique, Centre Armand Frappier Santé Biotechnologie, Laval, Quebec, Canada.; 5 Department of Pathology and Laboratory Medicine, Mount Sinai Hospital,; 6 The Prenatal Diagnosis and Medical Genetics Program, Mount Sinai Hospital, and; 7 Division of Clinical and Metabolic Genetics, Hospital for Sick Children, University of Toronto, Toronto, Ontario, Canada.; 8 Department of Clinical and Movement Neurosciences, UCL Queen Square Institute of Neurology, University College London, London, United Kingdom.; 9 Prenatal Diagnosis & Fetal Medicine Department, Human Genetics and Genome Research Institute, National Research Centre, Cairo, Egypt.; 10 King’s College Hospital, Synnovis, Denmark Hill, London, SE5 9RS and Department of Medical & Molecular Genetics, Faculty of Life Sciences and Medicine, King’s College London, United Kingdom.; 11 Cellular Pathology Department, Birmingham Women’s and Children’s NHS Foundation Trust, Birmingham, United Kingdom.; 12 Department of Obstetrics and Gynecology, Semmelweis University, Baross u. 27, Budapest, Hungary.; 13 West Midlands Regional Genetics Service, Birmingham Women’s and Children’s NHS Foundation Trust, Birmingham, United Kingdom.; 14 Movement Disorders Unit, Pediatric Neurology Department, Institut de Recerca, Sant Joan de Déu Barcelona Children’s Hospital, Barcelona, Spain.; 15 European Reference Network for Rare Neurological Diseases, Barcelona, Spain.; 16 U-703 Centre for Biomedical Research on Rare Diseases, Instituto de Salud Carlos III, Barcelona, Spain.; 17 Department of Genetic,; 18 Diagnostic Imaging Department, and; 19 Institut de Recerca Sant Joan de Déu, Sant Joan de Déu Barcelona Children’s Hospital, Esplugues de Llobregat, Spain.; 20 GeneDx, LLC, Gaithersburg Maryland, USA.; 21 Department of Human Genetics, Radboud University Medical Center, Nijmegen, Netherlands.; 22 Department of Clinical Genetics, Human Genetics and Genome Research Institute, National Research Centre, Cairo, Egypt.; 23 CENTOGENE, Rostock, Germany.; 24 Department of Medical Genomics, Genomic Medicine Centre of Excellence, King Faisal Specialist Hospital & Research Center, Riyadh, Saudi Arabia.; 25 Department of Diagnostic and Interventional Radiology, Hospital for Sick Children, and; 26 Ontario Fetal Center, Department of Obstetrics and Gynecology, Mount Sinai Hospital, University of Toronto, Toronto, Ontario, Canada.; 27 Genomic Precision Diagnostic Department, Igenomix FZ LLC, Dubai, United Arab Emirates.; 28 Department of Genetics, Sultan Qaboos University Hospital, University Medical City, Muscat, Oman.; 29 Department of Genetics, College of Medicine and Health Sciences, Sultan Qaboos University, Muscat, Oman.; 30 Biochemistry III, Department of Chemistry, University of Bielefeld, Bielefeld, Germany.; 31 Neuroradiology Unit, Istituto di Ricovero e Cura a Carattere Scientifico Giannina Gaslini, Genova, Italy.; 32 Département de Neurosciences, Université de Montréal, Montréal, Quebec, Canada.

**Keywords:** Cell biology, Genetics, Neuroscience, Lysosomes, Neurodegeneration, Neurodevelopment

## Abstract

*BORCS5* encodes a subunit of the BLOC-One-Related Complex (BORC), which is known to promote anterograde movement and fusion of lysosomes. We identified 16 individuals from 9 families with bi-allelic *BORCS5* variants, revealing a spectrum of neurodevelopmental and neurodegenerative phenotypes. Carriers of homozygous protein-truncating variants (PTVs), resulting in complete loss of BORCS5, presented with prenatally lethal arthrogryposis multiplex congenita, brain malformations, and neuropathological evidence of neuroaxonal dystrophy. Individuals with missense or splice-site variants presented differently, with microcephaly, developmental epileptic encephalopathy, optic atrophy, spasticity, and progressive movement disorders. In this group, brain MRI showed diffuse hypomyelination, corpus callosum abnormalities, and progressive global cerebral atrophy, consistent with neurodegeneration. *Borcs5* KO in zebrafish resulted in microcephaly, motor deficits, and increased seizure susceptibility, mirroring the patients’ clinical presentation. At the cellular level, only BORCS5 PTVs, but not missense variants, led to perinuclear lysosomal clustering and impaired lysosomal axonal trafficking in induced pluripotent stem cell–derived forebrain neurons. However, PTVs and missense variants were associated with reduced lysosomal proteolysis and activity of lysosomal hydrolases glucocerebrosidase and cathepsin B, indicating lysosomal dysfunction. Our study reveals a role for BORCS5 in modulation of lysosomal function, in addition to its known role in lysosome movement and fusion, possibly underlying the diverse clinical manifestations in individuals with BORCS5-related disorders.

## Abstract 2

Our study identifies BORCS5 variants in cases with a broad spectrum of neurological disorders and reveals a novel role for BORCS5 in lysosomal function.

## Introduction

*BORCS5* (Mendelian Inheritance in Man [MIM] *616598; previously known as *LOH12CR1*) encodes the 196–amino acid protein BLOC-One-Related Complex (BORC) subunit 5 (BORCS5) (also known as myrlysin), a subunit of BORC. BORC is a ubiquitously expressed protein complex composed of 8 subunits (named BORCS1–8) (1), which is known for promoting the interaction between lysosomes and kinesins via ARL8 and its effector SKIP, allowing for microtubule-mediated anterograde trafficking of lysosomes (2, 3). In neurons, BORC controls anterograde movements of lysosomes, particularly in axons (4–6), but the consequences of disrupted axonal transport of lysosomes in neurological disorders are not fully understood.

BORC also mediates axonal transport of RNA granules hitchhiking on lysosome-related structures (7). Disruption of BORC resulted in depletion of axonal mRNAs encoding proteins essential for mitochondrial function (7). Additional functions attributed to BORC include autophagosome-lysosome fusion via the homotypic fusion and protein sorting (HOPS) complex (8) and maturation of late endosomes to lysosomes (9). Loss of either Borcs5 or Borcs7 in mouse models results in clustering of lysosomes in the soma and their depletion from axons, leading to axonal pathology, motor dysfunction, and perinatal respiratory failure (4, 6). Moreover, bi-allelic pathogenic variants in *BORCS8* were recently identified in 3 unrelated families with an infantile-onset neurodegenerative disorder (MIM 620987) (10).

We report the identification of bi-allelic pathogenic variants in *BORCS5*, including protein-truncating variants (PTVs) and splice-site and missense variants, in 16 individuals from 9 unrelated families. The clinical presentation of these cases ranged from perinatally lethal arthrogryposis multiplex congenita associated with brain malformations and neuropathological evidence of neuroaxonal dystrophy (NAD), to a severe neurodegenerative phenotype with developmental epileptic encephalopathy and subsequent progressive movement disorders. Cellular studies showed that only *BORCS5* PTVs, causing complete loss of function (LoF), resulted in perinuclear lysosomal clustering, whereas both PTVs and missense variants were associated with features of lysosomal dysfunction. Our data suggest an additional role for BORCS5 in the modulation of lysosomal function. The impact of *BORCS5* variants on different aspects of lysosomal biology may underlie the different clinical presentations observed in individuals with *BORCS5*-related neurological disorders.

## Results

### Identification of biallelic BORCS5 variants in affected patients from 9 unrelated families.

**Figure 1:**
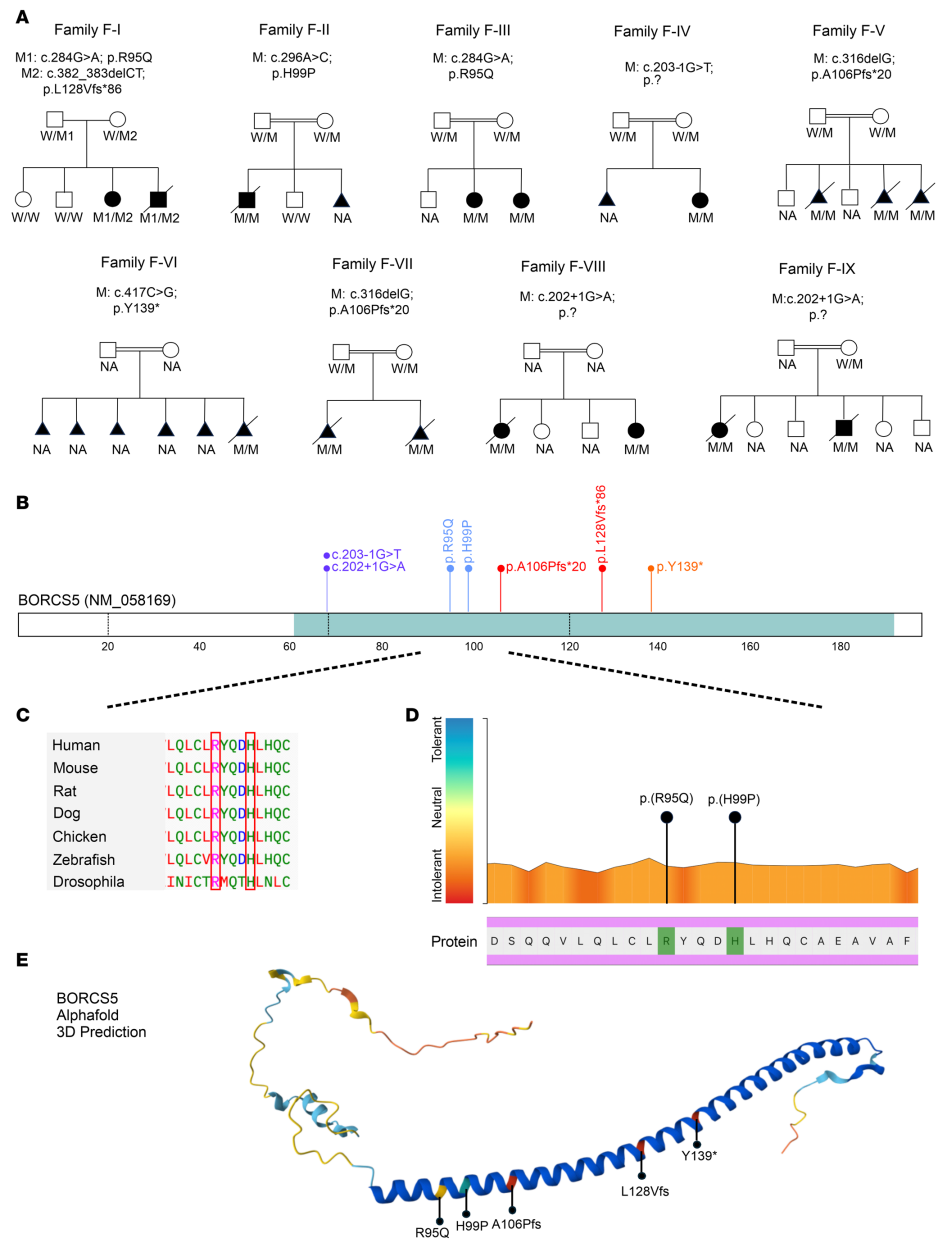
Family pedigrees and bi-allelic *BORCS5* variants. (**A**) Family pedigrees and genotypes of cases with bi-allelic *BORCS5* pathogenic variants. Triangles indicate spontaneous miscarriages, and crossed triangles indicate elective pregnancy terminations. (**B**) Schematic of BORCS5 protein indicating the position of the identified pathogenic *BORCS5* variants. (**C**) Conservation across species of the amino acid residues involved by the identified pathogenic missense variants R95Q and H99P. (**D**) Graphic representation of intolerance to *BORCS5* variants. Using Metadome software (https://stuart.radboudumc.nl/metadome/), we mapped the identified missense variants, which both affect amino acid residues that show marked intolerance to their variation. (**E**) Structure of BORCS5 predicted by AlphaFold and localization of coding variants identified in this study.

Sixteen affected patients from 9 unrelated families of Pakistani (F-I, F-V, and F-VII), Moroccan (F-II), and Arab descent (F-III, F-IV F-VI, F-VIII, F-IX), were recruited into the study (Figure 1A).

In total, we identified 7 pathogenic variants in *BORCS5* (NM_058169.4): 2 missense variants (c.284G>A, p.R95Q and c.296A>C, p.H99P), 2 essential splice-site variants (c.202+1G>A and c.203-1G>T), and 3 PTVs, including 2 frameshift deletions (c.316delG, p.A106Pfs*20 and c.382_383delAG, p.L128Vfs*86) and 1 STOP-gain variant (c.417C>G, p.Y139*) (Figure 1B).

In the index pedigree (family F-I), quad familial research whole-exome sequencing of DNA from the 2 affected siblings (F-I:1 and F-I:2) and their healthy parents identified 2 rare variants in *trans* in *BORCS5*: the maternally inherited frameshift variant L128Vfs*86 and the paternally inherited missense variant R95Q. Sanger sequencing confirmed the variants and showed that the 2 older unaffected siblings were not carriers.

Fourteen additional cases from 8 families with *BORCS5* bi-allelic variants were subsequently identified. Based on segregation analysis, *BORCS5* variants were independently prioritized as the top candidate genetic cause in each family.

Only affected family members from F-I had compound heterozygous *BORCS5* variants, whereas all other affected individuals carried homozygous variants. The 2 affected cases from family F-III carried the homozygous missense variant R95Q, which was also found in family F-I. The same homozygous frameshift variant A106Pfs*20 was identified in a total of 5 individuals from 2 unrelated Pakistani families (F-V and F-VII). The homozygous splice-site variant c.202+1G>A was identified in 4 individuals from 2 unrelated families from Oman (F-VIII and F-IX). The homozygous variants H99P (F-II:1), c.203-1G>T (F-IV:1), and Y139* (F-VI:1), were identified in single individual cases.

All identified *BORCS5* variants are absent or exceedingly rare in gnomAD or other large datasets of genomic variation (Supplemental Table 1; supplemental material available online with this article; https://doi.org/10.1172/JCI195336DS1).

The missense variants R95Q and H99P have Combined Annotation–Dependent Depletion scores greater than 20, which places them in the top 1% most deleterious changes for the human genome, and are predicted to be damaging by all available in silico tools. Furthermore, both variants affect amino acid residues that are fully conserved across species and localized in a protein region highly intolerant to genetic variation (Figure 1, C and D).

The AlphaFold prediction of the BORCS5 protein structure indicates that R95 and H99 are part of a long α helix likely to be essential for its interaction with other BORC subunits (Figure 1E). The PTVs A106Pfs* and Y139* are predicted to cause an early protein truncation, removing a large proportion of the BORCS5 α-helical domain. The L128Vfs allele is predicted to lead to a profound alteration in the amino acid composition of the C-terminus of BORCS5, extending the protein by 16 amino acids and likely resulting in a severe disruption of the protein α-helix. The splice-site variants c.202+1G>A and c.203−1G>T are predicted by SpliceAI (11) to result in complete loss of the donor and acceptor splice sites of intron 2, respectively.

### BORCS5 bi-allelic variants cause a broad phenotypic spectrum.

Detailed clinical presentations of the 16 affected individuals with bi-allelic *BORCS5* variants are included in Table 1 and Supplementary Material.

Ten individuals from families F-I, F-II, F-III, F-IV, F-VIII, and F-IX shared a core phenotype of developmental encephalopathy characterized by profound intellectual disability, delayed milestones, seizures, and progressive movement disorders with spasticity. Patients from families F-I, F-II, and F-III harbored BORCS5 missense variants (R95Q or H99P), either in compound heterozygosity with PTV (F-I) or in homozygosity (F-II and F-III). Individuals from families F-IV, F-VIII, and F-IX carried splice-site variants (c.202+1G>A in F-VIII and F-IX; c.203-1G>T in F-IV). The patient from F-IV was previously reported on with minimal clinical details (12).

None of these individuals attained speech or walking abilities, and they were completely dependent on others. Developmental regression, including loss of babbling, visual tracking, head control, and ability to stand, was observed in the patients from F-I, F-II, F-VIII and F-IX. Seizures occurred within the first year in all patients and were managed effectively with standard antiepileptic treatments in families F-I, F-II, and F-III. Older individuals from F-I became seizure-free in their teens. Conversely, affected individuals from F-IV and F-VIII had a more severe epileptic phenotype, presenting with treatment-resistant infantile spasms and myoclonic seizures and an EEG showing hypsarrhythmia.

All individuals exhibited progressive spasticity, diffuse hyperreflexia, bilateral extensor plantar response, and severe limb contractures. Movement disorders (dystonia and parkinsonism) were prominent in families F-I and F-II. Generalized dystonia led to scoliosis, painful spasms, and oculogyric crises. Parkinsonian features included severe limb bradykinesia and hypomimia. Dystonic spasms improved dramatically with carbidopa/levodopa treatment, suggesting nigrostriatal dopaminergic denervation. Other medications, including trihexyphenidyl, baclofen, and diazepam, helped manage spasticity and dystonia.

Optic neuropathy was present in all patients. Nerve conduction studies revealed sensorimotor demyelinating neuropathy in F:II-1 but were normal in F:I-1. Dysmorphic features included microcephaly, dolichocephaly, and low-set ears. Swallowing difficulties necessitated percutaneous endoscopic gastrostomy in cases from families F-I and F-II.

Five patients (F-I:2, F-II:1, F-VIII:1 and 2, and F-IX:1) died prematurely from aspiration pneumonia or uncontrolled seizures. The remaining 5 individuals were aged 28 (F-I:1), 23 (F-III:1), 13 (F-III:2), 10 (F-IV:1), and 3 (F-VIII:2) years at their most recent evaluation.

**Figure 2:**
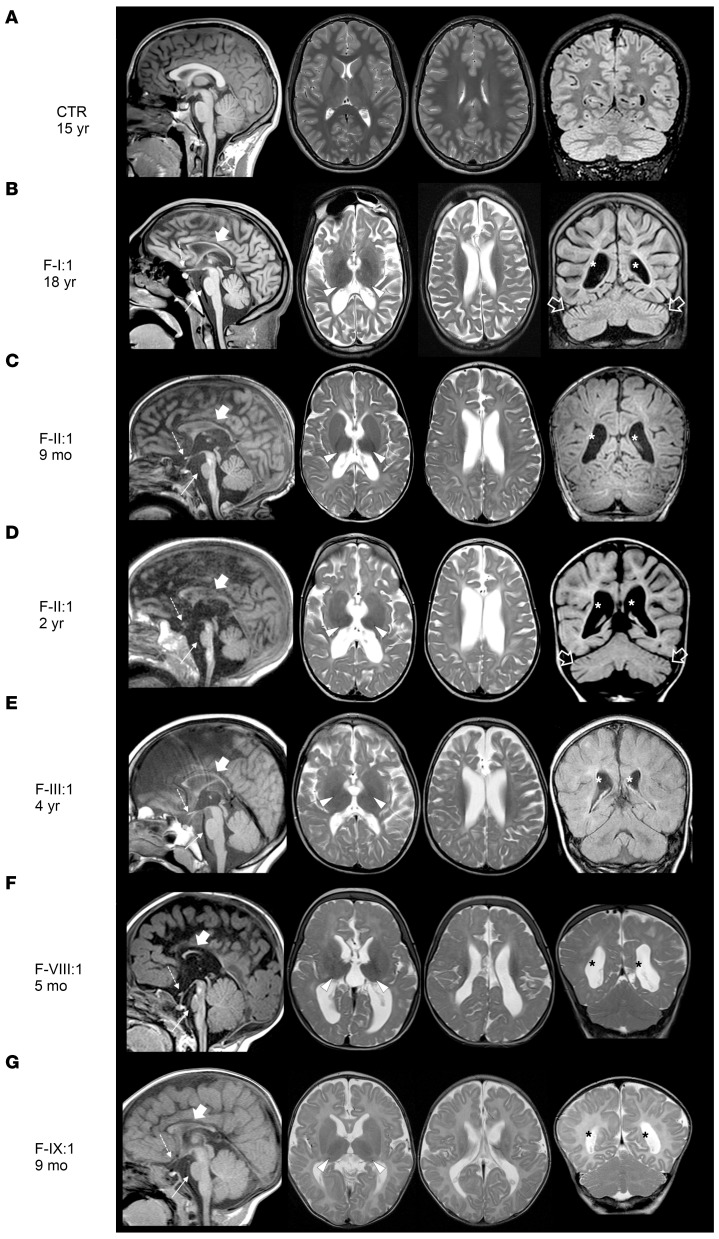
Neuroimaging findings in individuals with *BORCS5* missense and splice-site variants. Brain MRI studies of a control individual performed at 15 years of age for comparison (**A**) and of individuals F-I:1 (**B**), F-II:1 (**C** and **D**), F-III:1 (**E**), F-VIII:1 (**F**), and F-IX:1 (**G**). Presented are sagittal T1-weighted images (first column), axial T2-weighted images (second and third column), and coronal FLAIR or T1/T2-weighted images (last column). There is moderate to severe cerebral atrophy and loss of white matter volume with consequent ventricular dilatation in all individuals (marked with *). The myelination is markedly reduced or incomplete in all cases. The corpus callosum is very thin in all individuals (thick arrows) with associated hypoplastic anterior commissure. In F-VIII:1 and F-IX:1, the corpus callosum is also short and dysplastic. There is atrophy of the optic nerves (not shown) and chiasm (dashed arrows) in all patients. The thalami are small and hypointense on T2-weighted images (arrowheads). The midbrain and pons are small in all individuals (thin arrows), especially F-II:1 and F-VIII:1. Mild atrophy is noted in F-I:1 and F-II:1 (empty arrows). Note the clear progression of cerebral and cerebellar atrophy with arrested myelination in FII:1 (**C** and **D**).

Neuroimaging revealed cerebral atrophy, white matter loss, hypomyelination, small T2-hypointense thalami, thin brainstem, and optic nerve atrophy in 9 individuals from F-I, F-II, F-III, FV-III, and F-IX (Figure 2, A–G). F-I:1 and F-II:1 also had mild cerebellar atrophy (Figure 2, B–D). Changes in F-II:1 progressed rapidly between 9 months and 2 years, indicating an aggressive neurodegenerative process (Figure 2, C and D). Five patients with homozygous splice-site variants from families F-IV, F-VIII, and F-IX also had developmental anomalies such as corpus callosum dysgenesis and polymicrogyria (Figure 2, F and G).

Six fetuses from families F-V, F-VI, and F-VII and who had biallelic *BORCS5* PTVs (A106Pfs or Y139*) had a distinct phenotype with severe prenatal neurological manifestations, initially indicated by reduced fetal movements. Intrauterine imaging studies revealed arthrogryposis multiplex congenita, hydrocephalus with aqueduct stenosis, agenesis of the corpus callosum, markedly thin brainstem, cerebellar hypoplasia, and diffuse muscle atrophy in all these fetuses (Supplemental Figure 1A). Growth parameters and other organ systems were normal. These pregnancies were terminated between 21 and 25 weeks of gestation.

**Figure 3:**
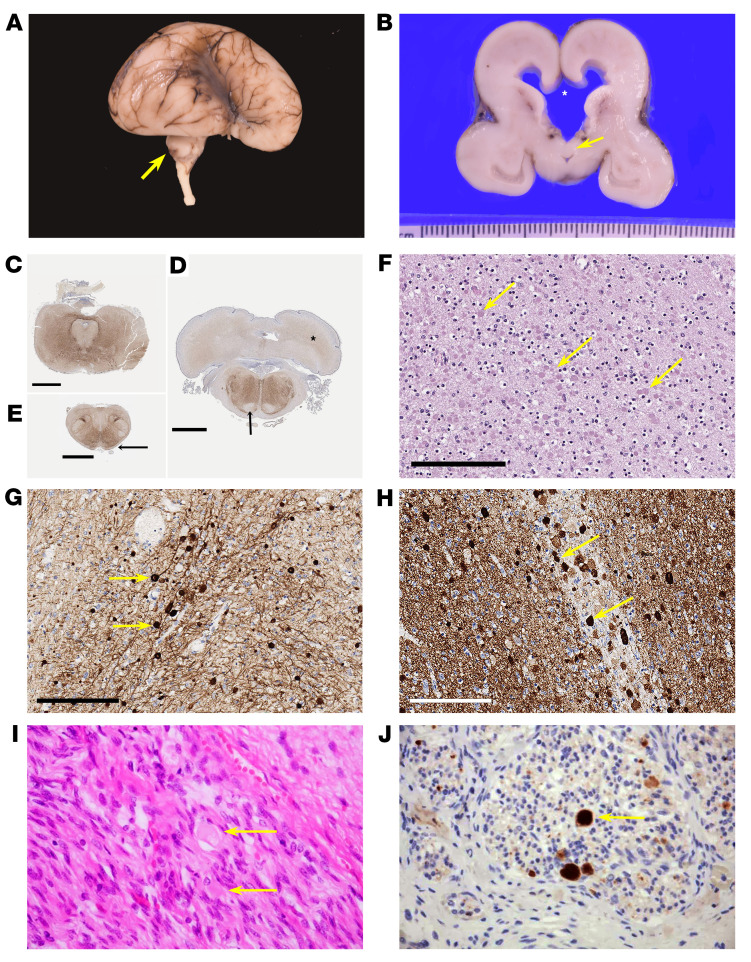
Pathological characterization of cases with bi-allelic LoF *BORCS5* variants. (**A**) Brain of case F-VII:1 demonstrating hypoplastic temporal lobes; a delayed, smooth Sylvian fissure; and a markedly hypoplastic cerebellum (arrow). (**B**) Coronal section, demonstrating ventriculomegaly. The corpus callosum is reduced to a thin membrane and has ruptured (indicated at *). The septum is ruptured, and the fornices (arrow) are descended and lie on the roof of the third ventricle. (**C**–**E**) Immunohistochemistry of neurofilament light chain in whole mounts of posterior fossa structures showing midbrain with minute aqueduct and hypoplastic cerebral peduncles (**C**); the caudal pons with very small and smooth inferior olivary nuclei, hypoplastic middle cerebellar peduncles, absent corticofugal tracts (arrow) and poorly defined dentate nuclei (*) (**D**); absent pyramids (arrows) and inferior cerebellar peduncles in the medulla (**E**). Scale bars: 3 mm (**C**–**E**). (**F**–**H**) Histological analysis of the same patient, with H&E staining and immunohistochemistry demonstrating innumerable pale eosinophilic axonal spheroids (arrows) in the internal capsule (**F**); strong positive staining of the axonal spheroids for neurofilament light chain (**G**) and α-synuclein (**H**). Scale bars: 200 μm (**F**–**H**). (**I** and **J**) Peripheral nerves of individual F-V:2 demonstrated numerous axonal spheroids (arrows) (**I**), which stained strongly positive for β-amyloid precursor protein (**J**).

Histopathological examination of the brain of each of 2 fetuses from families F-V and F-VII (Figure 3 and Supplemental Figure 1) revealed microcephaly, hypoplastic thalamus, basal ganglia and cerebellum, along with ventriculomegaly, aqueductal atresia, and absent corpus callosum (Figure 3, A and B). Brainstem examination showed hypoplasia of major neuronal groups, including tegmental nuclei, inferior olivary nuclei, basis pontis, and dentate nucleus (Figure 3, C–E) Furthermore, widespread neuroaxonal degeneration with formation of axonal spheroids, particularly in the brainstem, cerebral hemispheres, and peripheral roots and nerves were found (Figure 3, F and I, and Supplemental Figure 1, E and G). Immunohistochemistry revealed strong β-amyloid precursor protein, neurofilament, and α-synuclein staining of the axonal spheroids, consistent with a pathological diagnosis of NAD (Figure 3, G, H, and J, and Supplemental Figure 1, B and F). However, axonal spheroids did not stain positive for phosphorylated α-synuclein (Supplemental Figure 1C), tau, or TDP-43.

### Zebrafish borcs5-ko leads to neurodevelopmental defects.

To investigate the effect of loss of BORCS5 in an animal model, we generated zebrafish in which *borcs5* was knocked out (*borcs5*-ko), using CRISPR-Cas9 editing to induce targeted biallelic *borcs5* mutations directly in the injected embryos (F0 generation) (10, 13). The zebrafish genome encodes a single *borcs5* ortholog, sharing 84% amino acid identity with human *BORCS5*.

**Figure 4:**
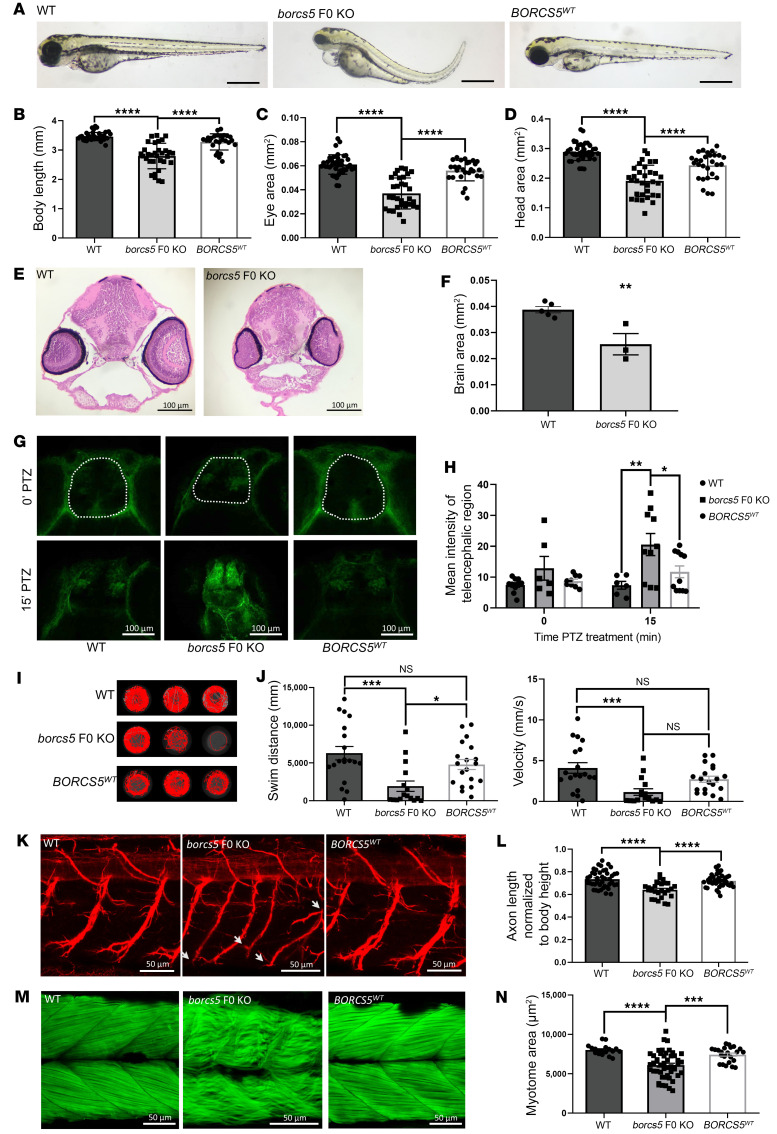
Zebrafish *borcs5*-ko larvae exhibit developmental defects and recapitulate patient symptoms. (**A**) Morphology of WT borcs5-ko and *BORCS5^WT^*-rescued larvae at 3 dpf. Scale bars: 500 μm. (**B**–**D**) Quantification of body length (**B**), eye size (**C**), and head size (**D**) in WT (*n* = 3, *n* = 40), borcs5-ko (*n* = 3, *n* = 32) and *BORCS5^WT^* (*N* = 3, *n* = 26–28) 3 dpf larvae. (Note *n* represents number of fish; *N* represents number of experimental repeats with each repeat consisting of embryos or larvae pooled from 3 independent clutches from different mating pairs.) (**E**) H&E staining of midbrain sections from 3 dpf WT and *borcs5-*ko larvae. Scale bar: 100 μm. (**F**) Quantification of brain area in WT (*N* = 5) and *borcs5*-ko 3 dpf (*N* = 3) larvae. (**G** and **H**) Neuronal activity assessed by p-MAPK/ERK fluorescence intensity in 4-dpf larvae after PTZ treatment (3 mM; 0 or 15 minutes). *borcs5*-ko (*N* = 2, *n* = 6–10) larvae showed significantly increased activity compared with WT (*N* = 2, *n* = 6–10) and *BORCS5^WT^* (*N* = 2, *n* = 9–11). Scale bars: 100 μm. (**I**) Representative swim trajectories of WT, *borcs5*-ko, and *BORCS5^WT^* larvae at 5 dpf. (**J**) Quantification of swim distance and velocity in WT (*N* = 1, *n* = 19), *borcs5*-ko (*N* = 1, *n* = 16), and *BORCS5^WT^* (*N* = 1, *n* = 19). (**K**) Acetyl tubulin staining of primary motor axon at 3 dpf. White arrows indicate axon branching defects in *borcs5*-ko larvae. Scale bars: 50 μm. (**L**) Axon length normalized by body height in WT (*N* = 2, *n* = 10), *borcs5*-ko (*N* = 2, *n* = 6), and *BORCS5^WT^* (*N* = 2, *n* = 10). (**M**) Phalloidin staining of muscle fibers at 3 dpf. Scale bars: 50 μm. (**N**) Quantification of dorsal or ventral myotome area in WT (*N* = 2, *n* = 9), *borcs5*-ko (*N* = 2, *n* = 10) and *BORCS5^WT^* larvae (*N* = 2, *n* = 12). Data are given as mean ± SEM. Statistical significance was calculated by 1-way ANOVA followed by Tukey’s multiple comparisons test (**B**–**D**, **H**, **J**, **L**, **N**), or Student’s *t* test (**F**). *P < 0.05; **P < 0.01; ***P < 0.001; ****P < 0.0001.

At 3 days after fertilization (3 dpf), *borcs5*-ko larvae exhibited reduced body size with a slight curvature of the body axis (Figure 4, A and B). Importantly, *borcs5*-ko larvae had reduced eye and head size relative to WT (Figure 4, C and D), and these gross morphological defects persisted at 5 dpf (Supplemental Figure 2, C–F). Injection of human *BORCS5^WT^* mRNA in *borcs5*-ko zebrafish rescued morphological phenotypes (Figure 4, B–D). Fewer dopaminergic neurons in *borcs5*-ko larvae were observed at 3 dpf compared with controls (Supplemental Figure 2, A and B). H&E staining revealed a reduction in overall brain size in *borcs5*-ko larvae compared with WT at 3 and 5 dpf (Figure 4, E and F, and Supplemental Figure 2, G and H), suggesting a critical role for *borcs5* in normal zebrafish brain development. We also observed larger ventricles in the *borcs5*-ko brain 5 dpf compared with WT controls (Supplemental Figure 2G).

Seizure susceptibility was examined at 4 dpf upon exposure to the pro-convulsive GABA receptor antagonist pentylenetetrazole (PTZ) (3 mM) (13–15). *Borcs5*-ko larvae exhibited increased p-MAPK/ERK staining compared with WT at 15 minutes after PTZ treatment, which was rescued in *BORCS5*^WT^ (Figure 4, G and H), suggesting abnormal neuronal hyperactivation in the *borcs5*-ko larval brain.

Locomotor activity (i.e., free swimming) was decreased in *borcs5*-ko larvae at 5 dpf, as compared with WT and to *BORCS5^WT^* (Figure 4, I and J). We also found that *borcs5*-ko larvae had shorter and less-branched axons in comparison with WT (Figure 4, K and L), and decreased area of dorsal and ventral myotomes with disorganization of somite structure and muscle fibers (Figure 4, M and N). The motor axonal and muscular defects in *borcs5*-ko zebrafish were rescued upon expression of the human *BORCS5* mRNA (*BORCS5*^WT^) (Figure 4, K–N).

Overexpression of R95Q and H99P mRNA in *borcs5*-ko embryos showed that BORCS5^R95Q^ and BORCS5^H99P^ zebrafish had shorter body length and reduced head and eye size, albeit to a lesser extent than *borcs5*-ko embryos (Supplemental Figure 2, I–L). We also found reduced swimming distance, reflecting locomotor dysfunction, in BORCS5^R95Q^ and BORCS5^H99P^ larvae.

Taken together, knockout of *borcs5* in zebrafish leads to neurodevelopmental defects, motor deficits, and increased seizure susceptibility, indicating that *borcs5* plays a role in the development and function of the CNS and supporting the pathogenic role of the identified variants.

### Effect of identified pathogenic variants on BORCS5 protein and BORC assembly.

**Figure 5:**
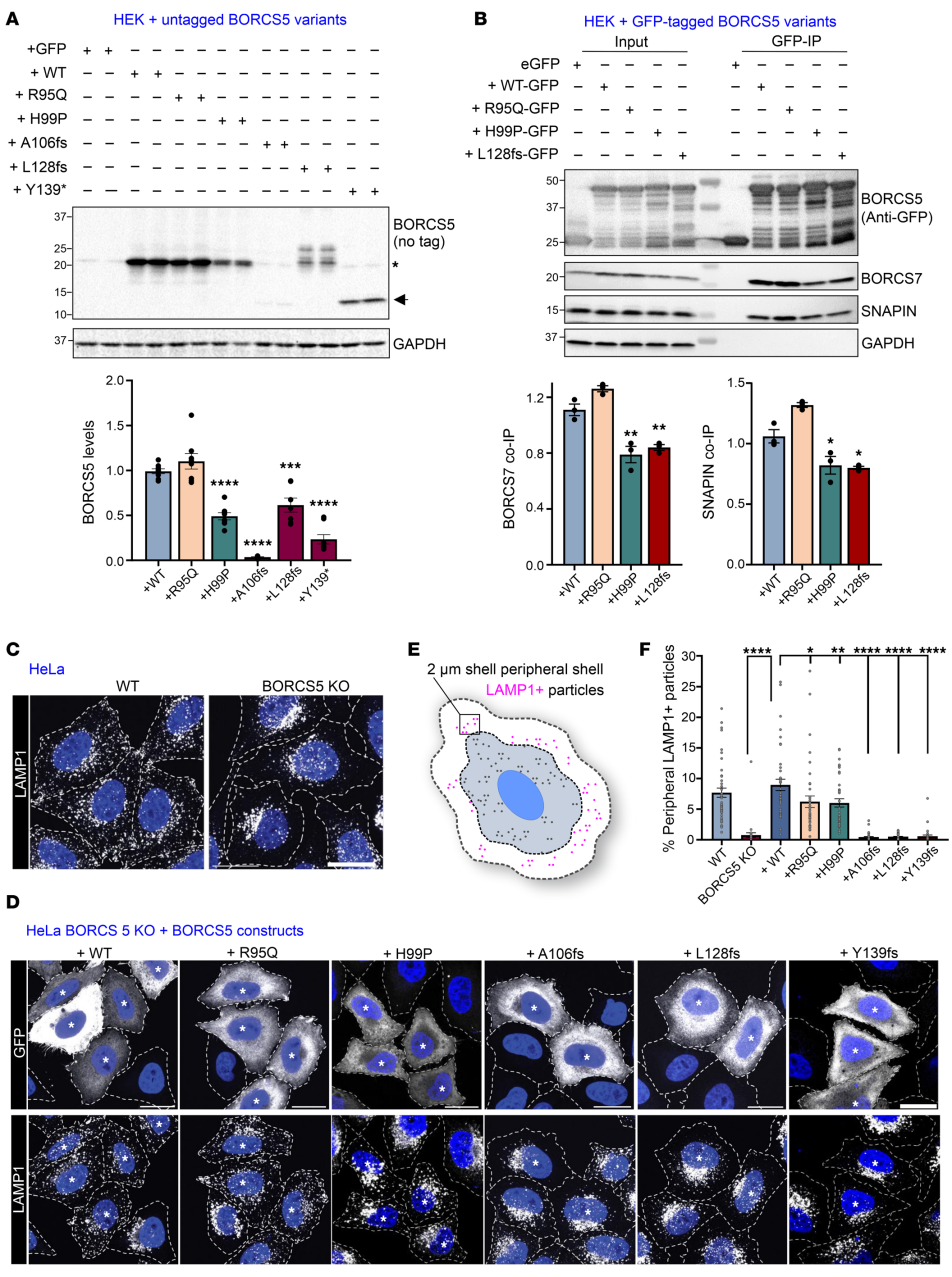
Impact of BORCS5 pathogenic variants on BORCS5 protein expression, BORC assembly, and endolysosome distribution in cell lines. (**A**) Western blot analysis of BORCS5 protein in HEK293T cells 24 hours after transfection with constructs expressing GFP (control) and BORCS5 WT or the indicated BORCS5 patient variants. GAPDH was used as a loading control. For WT and R95Q/H99P, the band indicated by * was quantified, whereas the band indicated by an arrow was quantified for A106fs and Y139*. In the case of L128fs, the entire band pattern was measured. The 25 kDa band present in the case of A106fs and Y139* variants correspond to endogenous BORCS5, as shown in GFP-only condition. The graph shows the mean ± SEM of fold change over the mean of the WT variant (*n* = 4 independent experiments). For BORCS5 levels, 1-way ANOVA F_(5,41)_ = 59.49 (*P* < 0.0001) with Dunnett’s post hoc test compared with WT. ***P = 0.0001, ****P < 0.0001. (**B**) GFP trap precipitation of GFP-tagged BORCS5 variants and their interaction (co-IP) with endogenous SNAPIN and BORCS7, with GAPDH used as a specificity control. The graph shows mean ± SEM n of 3 independent experiments. For BORCS7, 1-way ANOVA with Dunnett’s post hoc test F_(3,8)_ = 33.17 (*P* < 0.0001). **P ≤ 0.003. For SNAPIN, F_(3,8)_ = 26.53 (*P* = 0.0002). *P < 0.018. (**C** and **D**) Immunofluorescence microscopy shows endogenous LAMP1 puncta (white) distribution in untransfected WT and BORCS5 KO HeLa (as control) or BORCS5-KO HeLa cells transiently cotransfected with BORCS5 constructs and GFP (* indicates GFP^+^ cells). Nuclei were labeled with DAPI (blue), and cell edges were outlined by fluorescent phalloidin (dashed lines). Scale bars: 20 μm. (**E** and **F**) Schematic depicts LAMP1^+^ endolysosomes present in a 2 μm peripheral shell, shown in the graph as mean ± SEM for 3 independent experiments, each dot representing a different cell. For peripheral lysosomes, 1-way ANOVA with Dunnett multiple comparisons test (compared with the WT construct), F_(7,289)_ = 30.96 (*P* < 0.0001). *P < 0.05, **P = 0.0098, ****P < 0.0001.

To analyze the impact of individual coding variants on BORCS5 protein expression, BORCS5 WT and mutant constructs were transiently expressed in HEK293T. Protein expression was comparable between WT and the R95Q missense variant, whereas it was 50% less in H99P than in the WT. The PTVs A106Pfs and Y139* exhibited aberrant, shorter protein products with lower protein amounts (Figure 5A), suggesting early truncation and reduced protein expression. Expression of L128Vfs showed multiple bands between 15 and 30 kDa (Figure 5A) that are likely degradation products.

Furthermore, we examined the interaction of *BORCS5* pathogenic variants with the BORC subunits SNAPIN and BORCS7, using GFP trap precipitation upon expression of C-terminally GFP-tagged BORCS5 constructs (Figure 5B). We found 30% less endogenous BORCS7 and 20% less endogenous SNAPIN coprecipitating with the H99P and L128Vfs constructs, whereas R95Q did not affect the BORCS5-BORCS7 or BORCS5-SNAPIN interactions (Figure 5B). The A106Pfs and Y139* variants were not included in this experiment, due to low baseline protein expression levels (Figure 5A).

### BORCS5 PTVs, but not the missense variants R95Q and H99P, cause abnormal perinuclear clustering of lysosomes.

Because the BORC complex regulates the transport of endolysosomes from the perinuclear region toward the cell periphery (1, 5), we compared endolysosomal distribution in HeLa cells overexpressing WT or BORCS5 pathogenic variants (Figure 5, C–F). Loss of endogenous BORCS5 led to a perinuclear accumulation of endolysosomes, reflected by a 90% reduction of LAMP1^+^ puncta in the cell periphery of BORCS5-KO cells, restored by reintroducing BORCS5 WT (Figure 5, C and F). Expression of A106Pfs, L128Vfs, and Y139* did not rescue the perinuclear endolysosome accumulation, confirming that these variants abolish BORCS5 function (Figure 5, D and F). In contrast, R95Q and H99P expression led to approximately 30% fewer peripheral lysosomes compared with WT, suggesting a partial rescue of perinuclear endolysosomal clustering (Figure 5, D and F).

**Figure 6:**
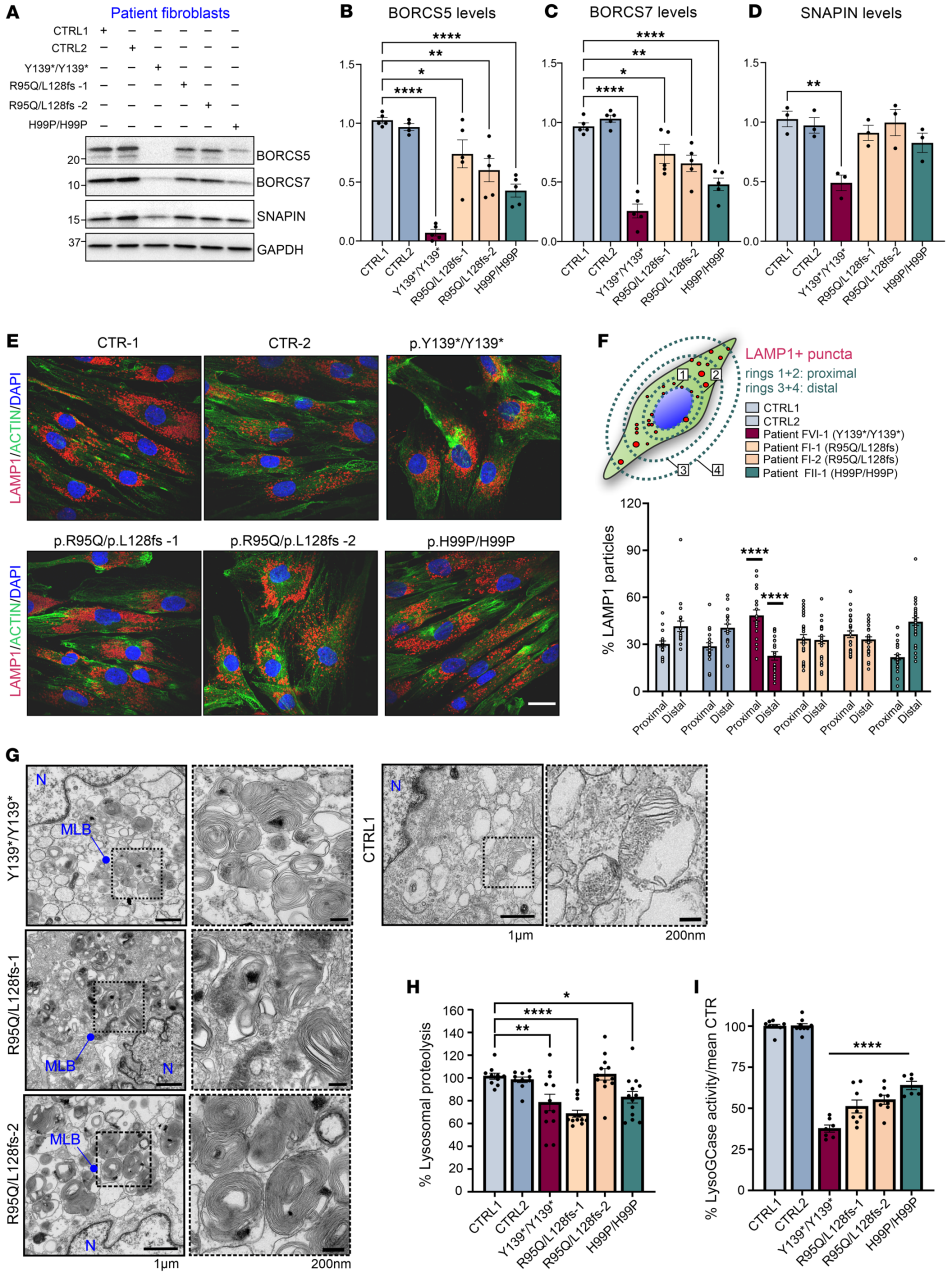
BORC-related protein expression, endolysosomal distribution, and function in fibroblasts of patients with BORCS5 pathogenic variants or 2 independent control lines. (**A**) Western blot analysis of proteins BORCS5, SNAPIN, and BORCS7; GAPDH was used as a loading control. (**B**–**D**) Graphs represent mean ± SEM of 3–5 independent experiments. For BORCS5, 1-way ANOVA with Dunnett’s post hoc test (compared with control 1 [CTRL1]), F(5,23) = 24.56 (*P* < 0.0001; for BORCS7, F_(5,24)_ = 27.02 (*P* < 0.0001); and for SNAPIN, F_(5,12)_ = 6.704 (*P* = 0.0034). *P < 0.03, **P < 0.003, ****P < 0.0001. (**E**) Immunofluorescence microscopy shows endogenous LAMP1 puncta distribution. Scale bar: 25 μm. (**F**) Schematic shows the analysis of LAMP1^+^ puncta distribution in fibroblasts, using elliptical concentric rings of 1.5 increments from the nucleus (ring 1 outlined). Puncta within rings 1 and 2 were designated “proximal” to the nucleus, and those between rings 3 and 4 were designated “distal.” The graph represents the mean ± SEM from 3 independent experiments, each dot representing a different cell. For the interaction, 2-way ANOVA F_(5,276)_ = 22.86 (*P* < 0.0001), with Tukey’s post hoc test (compared with CTRL1/CTRL2). (**G**) TEM micrographs of BORCS5 patient or control fibroblasts, with dashed lines indicating magnification of the outlined insets. MLB, multilamellar body; N, nucleus. (**H**) Lysosomal proteolysis efficiency was assessed upon administration of 25 μg/mL of DQ Green or Red BSA for 5 hours, in 10,000 single-cell events, via flow cytometry. The graph represents the mean ± SEM of the percent median fluorescence intensity, normalized to the mean of control lines (*n* = 8–9 independent experiments). For proteolysis, 1-way ANOVA with Dunnett’s post hoc test (compared with CTRL1), F_(5,63)_ = 8.687 (*P* < 0.0001). *P = 0.038, **P = 0.007, ****P < 0.0001. (**I**) Lysosomal GCase activity was assessed upon administration of 250 μM PFB-FDGlu for 30 minutes, in 10,000 single-cell events, via flow cytometry. The graph represents the mean ± SEM of the percent median fluorescence intensity, normalized to the mean of control lines. CTR-1/CTR-2, *n* = 7; p.Y139*/p.Y139* and p.R95Q/L128Vfs*86, *n* = 4; p.H99P/H99P, *n* = 3 independent experiments. For lysoGCase, 1-way ANOVA with Dunnett’s post hoc test (compared with CTRL1), F_(5,41)_ = 121.3 (*P* < 0.0001). ****P < 0.0001.

BORC assembly and lysosomal distribution were also examined in skin fibroblasts from patients carrying missense variants (F-I:1 and F-I:2 with compound heterozygous variants R95Q/L128Vfs and patient F-II:1 with homozygous H99P/H99P) and from a patient with a homozygous PTV (F-V:6 with homozygous Y139*/Y139*). All lines exhibited lower BORCS5 protein, with the 2 fibroblast lines R95Q/L128Vfs showing a 25%–40% reduction, the H99P/H99P line with a 60% reduction and the Y139*/Y139* line having 93% lower BORCS5 protein as compared with control (Figure 6, A and B).

BORCS5 mRNA transcript was reduced in the Y139*/Y139* line, consistent with nonsense-mediated decay contributing to decreased protein expression (Supplemental Figure 3A). In contrast, BORCS5 RNA levels were preserved in the 2 R95Q/L128Vfs lines, indicating that the L128Vfs variant does not impair mRNA stability (Supplemental Figure 3A).

Additionally, because loss of 1 BORC subunit affects the stability of other subunits (10), we examined the endogenous levels of SNAPIN and BORCS7. Fibroblasts from the 2 R95Q/L128Vfs patients showed 25-35% lower BORCS7, H99P/H99P showed 50% less and Y139*/Y139* fibroblasts had 75% lower protein (Figure 6, A and C). In contrast, SNAPIN was exclusively reduced in fibroblasts from the Y139*/Y139* patient (Figure 6, A and D).

Endolysosomal distribution in fibroblasts from patients with BORCS5 pathogenic variants were examined by comparing the percentage of LAMP1^+^ puncta located proximally compared with distally to the nucleus (Figure 6, E and F). BORCS5 Y139*/Y139* fibroblasts had 60% more endolysosomes proximal to the nucleus and 40% fewer distal endolysosomes (Figure 6, E and F). However, no changes were found in fibroblasts from patients with BORCS5 R95Q/L128Vfs and H99P/H99P variants (Figure 6, E and F).

These results indicate that A106Pfs, Y139*, and L128Vfs are complete LoF alleles, whereas the H99P is a hypomorphic allele associated with reduced endogenous protein levels. Complete loss of BORCS5 likely affects the assembly of BORC and the protein levels of its subunits. The R95Q allele is normally expressed and is not likely to affect the interaction with other BORC subunits and BORC assembly. Furthermore, BORCS5 PTV variants led to perinuclear lysosomal clustering, whereas the missense BORCS5 variants R95Q and H99P preserved, at least in part, anterograde endolysosomal transport.

### Both protein-truncating and missense BORCS5 variants lead to lysosomal dysfunction.

Loss of BORC subunits, including BORCS5, reduces autophagosome-to-lysosome fusion (8), leading to intracellular accumulation of autophagosomes. Consistent with this notion, BORCS5-KO HeLa cells have an increased number of puncta positive for the autophagosomal marker LC3, a phenotype that could be restored by expressing WT BORCS5 (Supplemental Figure 3, B–D). The expression of all PTVs failed to rescue the KO phenotype; however, expression of the missense variants R95Q and H99P restored LC3 to WT levels (Supplemental Figure 3, B–D), suggesting that these variants do not affect autophagosome clearance.

We further investigated endolysosomal function in fibroblasts in patients with affected BORCS5. Transmission electron microscopy (TEM) revealed the presence of multilamellar bodies, structures related to dysfunctional lysosomes (16), in 20% of the examined fibroblasts with complete BORCS5 LoF (Y139*/Y139*) as well as in 10% to 40% of those carrying the missense variant R95Q (R95Q/L128Vfs) (Figure 6G).

The efficiency of lysosomal proteolysis was reduced by approximately 20% in all fibroblast lines with both LoF and missense pathogenic variants as compared with controls (Figure 6H). Examination of total cell lysates by Western blotting showed comparable amounts of the lysosomal membrane protein LAMP2, suggesting that decreased lysosomal proteolysis was not due to lower lysosome number (Supplemental Figure 4B). Notably, the activity of lysosomal hydrolase glucocerebrosidase (GCase) was approximately 40% lower in cells with BORSC5 missense variants and approximately 60% lower in the LoF line (substrate 5-(pentafluorobenzoylamino)fluorescein di-β-d-glucopyranoside [PFB-FDGlu]) (Figure 6I), whereas GCase protein and its lysosomal transporter LIMP2 were not changed (Supplemental Figure 4, B–D). Thus, both BORCS5 LoF and missense variants led to decreased lysosomal GCase activity and impaired general lysosomal proteolysis.

Because lysosomal dysfunction may increase the release of autophagic protein cargo in association with exosomes or extracellular vesicles (EVs) (9, 17, 18), we examined EV fractions isolated from identical volumes of conditioned medium across all fibroblast lines. All fractions were positive for standard exosomal markers (Supplemental Figure 5) (19). Importantly, EVs from both the BORCS5 complete LoF (Y139*/Y139*) and the missense fibroblast line (H99P/H99P) had increased amounts of the autophagy markers LC3-II and p62/SQSTM1, and of the lysosomal membrane marker LAMP2 (Supplemental Figure 5), supporting release of undegraded protein cargo associated with EVs.

### Defects in endolysosome distribution and lysosomal function in BORCS5-mutant induced pluripotent stem cells–derived forebrain neurons.

Given that all patients with *BORCS5* pathogenic variants exhibited exclusively neurological symptoms, we further examined the impact of complete BORCS5 LoF and the R95Q missense variant on trafficking and endolysosomal function in a human neuronal model.

To this end, we reprogrammed fibroblasts from the 2 affected individuals carrying R95Q/L128Vfs into induced pluripotent stem cells (iPSCs) and used an independent control iPSC line to generate an isogenic CRISPR/Cas9 *BORCS5* KO line. All iPSC lines were differentiated into human forebrain neurons (iNeurons) via Ngn2 overexpression (20).

**Figure 7:**
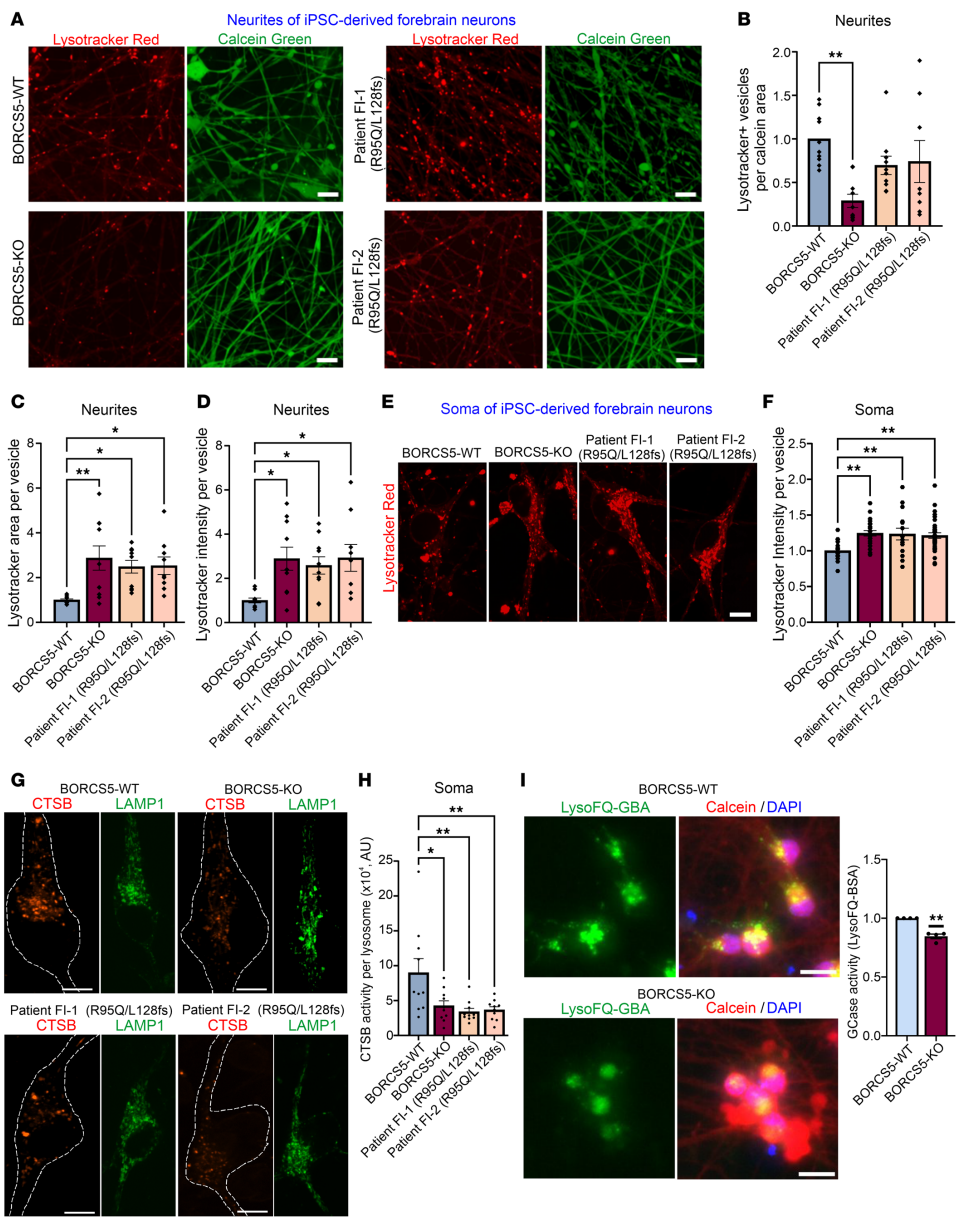
Lysosome-dispersal activity of patient-specific *BORCS5* variants. Increased LysoTracker and decreased lysosomal enzyme activity in neurons with patient-specific *BORCS5* variants. (**A**) Imaging of Lysotracker Red signal (red) in neurites of live iPSC-derived forebrain neurons (labeled with calcein, green) from WT, isogenic BORCS5-KO, or R95Q/L128fs patient iPSC lines. Scale bar: 10 μm. (**B**–**D**) Graphs show the mean ± SEM of 3 independent experiments. For vesicle number, 1-way ANOVA with Dunnett’s post-hoc test F_(3,34)_ = 5.250 (*P* = 0.0044); for vesicle area, F_(3,35)_ = 5.373 (*P* = 0.0038); for signal intensity, F_(3,35)_ = 4.362 (*P* = 0.0104). (**E**) Imaging of Lysotracker Red endolysosomes (red) in the soma of iPSC-derived forebrain neurons from the indicated lines. Scale bar: 10 μm. (**F**) The graph shows the mean ± SEM of 3 independent experiments. For signal intensity, 1-way ANOVA with Dunnett’s post hoc test F_(3,87)_ = 5.624 (*P* = 0.0014). (**G**) Fluorescence microscopy examination of LAMP1^+^ stained endolysosomes (green) and cathepsin B (CTSB) activity–derived fluorescent signal (red) in the soma of iPSC-derived forebrain neurons from the indicated lines. Scale bar: 10 μm. (**H**) The graph shows the mean ± SEM of 3 independent experiments. For CTSB activity soma, 1-way ANOVA with Dunnett’s post hoc test F_(3,36)_ = 5.478 (*P* = 0.00333). Dots represent individual cells. Scale bar: 10 μm. (**I**) Fluorescence microscopy shows viable (calcein; red) day 21 iNeurons upon uptake of the lysosomal GCase substrate LysoFQ-GBA (green) and counterstained with DAPI. Scale bar: 50 μm. The lysosomal GCase activity–dependent fluorescent signal was quantified, and the graph shows the mean ± SEM of the relative intensity over mean WT within a specific experiment (*n* = 4 independent experiments). One-sample *t* test. **P = 0.0042.

Using the fluorescent lysosomotropic dye Lysotracker Red, we found that BORCS5-KO neurites contained 60% fewer endolysosomes than did isogenic control neurons (Figure 7, A and B), which is in agreement with previous reports (4, 5, 7). However, no changes in endolysosomal distribution were observed in neurites of BORCS5-R95Q/L128Vfs versus WT neurons (Figure 7, A and B). Lysotracker Red^+^ puncta in BORCS5-KO and R95Q/L128Vfs neurons exhibited a larger area (Figure 7, A and C) and higher intensity per vesicle (Figure 7, A and D–F), which may reflect an accumulation of acidified structures. Such morphological alterations were paralleled by reduced activity of the lysosomal hydrolase cathepsin B, assessed via microscopy upon administration of Magic Red cathepsin B substrate (Figure 7, G and H). Furthermore, BORCS5-KO iNeurons had reduced lysosomal GCase activity, assessed via microscopy (LysoFQ-GBA substrate) (Figure 7I).

**Figure 8:**
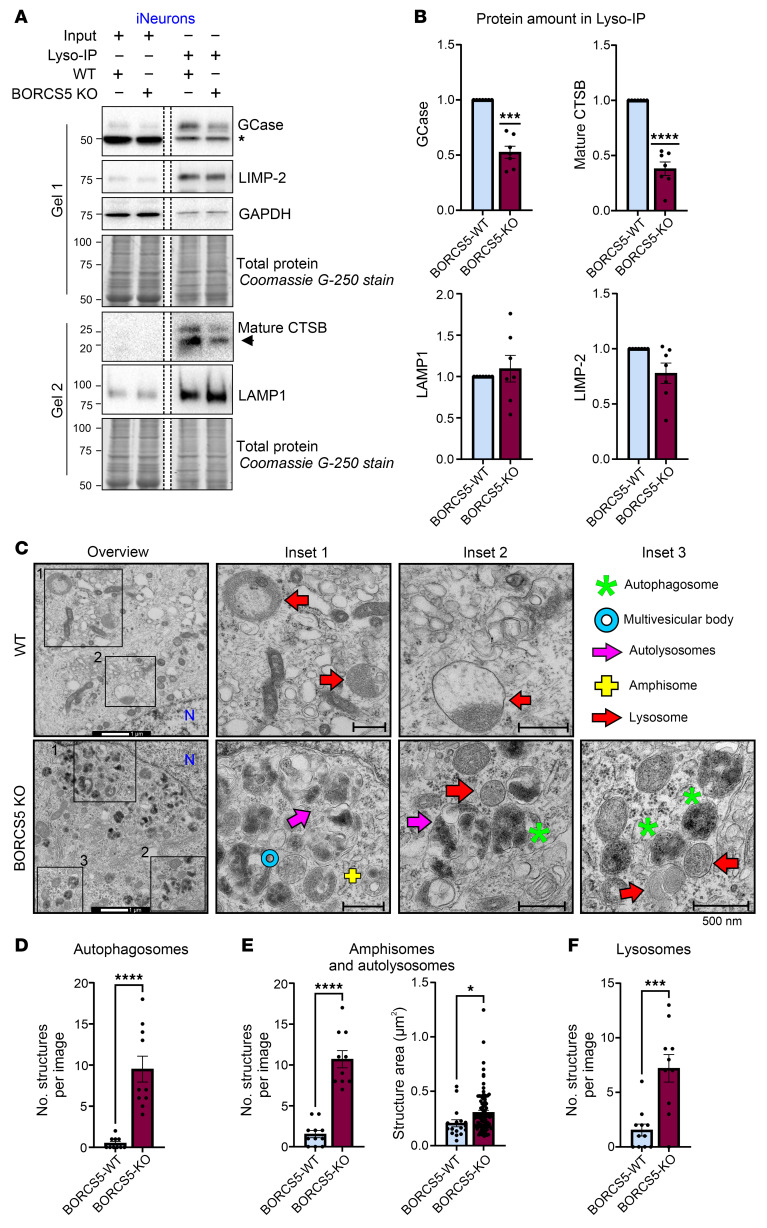
BORCS5 deficiency leads to lysosomal dysfunction in iNeurons. (**A**) Endolysosomal fractions were enriched from day 21 iNeurons expressing TMEM192-GFP-3xHA via magnetic immunopurification (Lyso-IP). Western blots of samples of the same experiment that were run in 2 different gels, with dashed lines reflecting that samples were loaded in different parts of the same gel, shown with the same exposure. Total protein was monitored by Coomassie G-250 staining of the gel after transfer. The * indicates an unspecific band at 50 kDa, and the arrow indicates the CTSB band quantified during Western blot analysis. (**B**) Graphs show the mean ± SEM of the relative protein amount normalized to the total protein, 1-sample *t* test, 7 independent experiments. ***P = 0.0001, ****P < 0.0001. (**C**) TEM images show the overview and magnified insets of the perinuclear cytoplasm from control and isogenic BORCS5 KO iNeurons. Individual autophagic and endolysosomal structures were classified based on morphological criteria. Scale bar: 1 μm (left panels), 500 nm (enlarged inset panels). (**D**–**F**) The number and/or area of the indicated structures are presented as mean± SEM obtained from 10–11 different cells per genotype. Unpaired *t* test. *P = 0.041, ***P = 0.0004, ****P < 0.0001.

To further elucidate the mechanisms underlying lysosomal dysfunction caused by BORCS5 deficiency, we assessed the amount of GCase and cathepsin B within the endolysosomal compartment. To this end, we expressed TMEM192-GFP-3xHA in WT and BORCS5-KO iNeurons, enabling affinity-based enrichment of endolysomes (21). Endolysosomal fractions from BORCS5-KO iNeurons exhibited an approximately 50% reduction in GCase protein and an approximately 60% reduction in mature cathepsin B compared with WT cells (Figure 8, A and B), whereas LIMP-2 and LAMP1 were not altered (Figure 8, A and B). The reduction in endolysosomal hydrolases could be partly attributed to decreased overall protein abundance in total neuronal lysates (Supplemental Figure 6).

To further characterize lysosomal dysfunction in BORCS5-deficient iNeurons, we performed TEM and quantified ultrastructural features associated with autophagy (autophagosomes, autolysosomes, and amphisomes) and the endolysosomal compartment (late endosomes or multivesicular bodies, and lysosomes) (Figure 8, C–F). BORCS5-KO iNeurons exhibited a 9-fold increase in the number of autophagosomes and autolysosomes or amphisomes, along with a 5.6-fold increase in lysosome number per micrograph compared with the control (Figure 8, C–F). These ultrastructural changes were corroborated by increased LC3-II and LAMP1 protein levels in total neuronal lysates (Supplemental Figure 6, A and B). Notably, induction of anterograde endolysosomal trafficking in BORCS5-KO cells by overexpression of a LAMP1–kinesin-binding sequence fusion protein — which bypasses BORC-dependent lysosomal positioning defects (1) — failed to restore LC3-II levels (Supplemental Figure 6C).

Collectively, complete loss of BORCS5 in neuronal cells disrupts anterograde axonal transport of endolysosomes and causes lysosomal dysfunction. Although these lysosomes can be acidified, they display a reduced capacity to clear autophagic cargo, even after fusion with autophagosomes. This impairment is likely, at least in part, due to decreased availability of specific lysosomal hydrolases within the endolysosomal compartment.

## Discussion

We provide genetic and functional evidence establishing *BORCS5* as a human disease–associated gene responsible for a severe neurological disorder characterized by a broad spectrum of phenotypes, from profound neurodevelopmental defects to infantile-onset neurodegeneration. Furthermore, we uncover a role for BORCS5 in the modulation of lysosomal function, expanding beyond its well-established function in mediating lysosome transport and fusion.

*BORCS5* homozygous PTVs, resulting in complete loss of BORCS5, were clinically associated with a severe, perinatally lethal phenotype with neuropathological evidence of NAD, a condition associated with extreme and rapidly progressive neurodegeneration. Similar pathological findings have also been reported in murine models of Borcs5 and Borcs7 complete LoF (4, 6).

These fetuses also displayed a severe developmental phenotype, characterized by agenesis of the corpus callosum, small brainstem and cerebellum, and aqueductal stenosis with supratentorial hydrocephalus. This suggests a critical role for BORCS5 in the development of the corpus callosum and midbrain-hindbrain structures, similar to what has been shown in individuals with pathogenic variants in *KIF5C and KIF2A*, which encode other critical components of the organelle anterograde transport machinery (22, 23).

In contrast, carriers of missense variants exhibited a chronic neurodegenerative presentation with infantile onset, associated with progressive movement disorders and severe spasticity. Neuroradiological findings in the latter group showed a profound neurodegenerative phenotype with early-onset global cerebral atrophy and hypomyelination, closely resembling features observed in individuals harboring *BORCS8* variants (10). Patients with homozygous splice-site variants showed a broadly similar presentation but with more severe epilepsy and more pronounced neuroimaging abnormalities, particularly involving corpus callosum development, consistent with an intermediate phenotype between complete BORCS5 LoF and missense variants.

The *borcs*5-ko zebrafish model recapitulated many of the phenotypic features observed in *BORCS5*-mutant cases, including microcephaly, ventriculomegaly, movement disorders, and seizure susceptibility. Thus, the *borcs5-*ko zebrafish model may be valuable to shed more light on the underlying pathophysiological mechanisms of the neurological defects associated with bi-allelic *BORCS5* variants.

Our study reveals a role for BORCS5 as a modulator of lysosomal function, expanding on its recognized role in anterograde lysosomal trafficking and autophagosome-lysosome fusion. Specifically, all fibroblast lines from patients showed decreased lysosomal proteolysis and reduced lysosomal GCase activity, and BORCS5 LoF fibroblasts showed release of autophagic or lysosomal protein cargo in association with EVs (9, 17, 18).

The multilamellar bodies found in fibroblasts from patients with affected BORCS5 have also been reported in patients with lysosomal storage disorders, including Niemann Pick disease types A and C, as well as those with Parkinson’s disease (PD) who carry pathogenic *GBA1* variants (16, 24–26). Moreover, individuals carrying *BORCS5* missense variants had clinical and radiological features resembling those seen in infantile-onset lysosomal storage disorders, such as gangliosidosis, fucosidosis, and neuronal ceroid lipofuscinoses (27). Similarly, in BORCS5-KO iNeurons, we observed accumulation of autolysosomes, indicating impaired ability to clear autophagic cargo.

The underlying mechanism of lysosomal dysfunction caused by *BORCS5* pathogenic variants remains to be fully elucidated. In iNeurons, BORCS5 depletion led to reduced lysosomal GCase and cathepsin B activity, partly attributed to their reduced levels within neuronal endolysosomes. The lower cathepsin B activity may, per se, impair lysosomal GCase activity through deficient processing of prosaposin into the endogenous GCase activator saposin C (28). Cathepsin B is also involved in processing of progranulin to granulin fragments that can modulate both GCase and lysosomal function (29, 30). BORCS5 deficiency inhibits late endosome-lysosome fusion, thus impairing delivery of endocytosed cargo to lysosomes for degradation (9, 31) and potentially hampering the delivery of lysosomal proteins trafficked via the endolysosomal compartment. Moreover, BORCS5 depletion led to reduced levels of cation-independent–mannose 6-phosphate receptor protein, which can impair the sorting of lysosomal hydrolases and luminal proteins to the endolysosomal compartment (32).

The role of BORCS5 in lysosomal function may have implications for other neurological disorders characterized by lysosomal dysfunction, including PD and dystonia. Intriguingly, several elements link BORCS5 dysfunction to PD pathogenesis. The genes encoding cathepsin B and glucocerebrosidase, whose activity is reduced by *BORCS5* pathogenic variants, are established risk factors for PD (33, 34). Additionally, we observed that the axonal spheroids in 1 of the individuals with *BORCS5* homozygous PTVs (F-VII:1) with NAD displayed strong staining for α-synuclein, the most abundant protein found in Lewy bodies (LBs). Moreover, the most common genetic cause of NAD are pathogenic variants in *PLA2G6*, a gene associated with a broad spectrum of neurodegenerative conditions, spanning from infantile NAD to early-onset PD (35, 36) and pathologically characterized by diffuse and severe α-synuclein pathological aggregation and formation of LBs (37, 38). Although we did not observe LBs in the brain of fetuses with *BORCS5* homozygous PTVs, this was likely due to their prenatal lethality. Finally, we observed loss of dopaminergic neurons in *borcs5*-ko zebrafish, a result that corroborates the response to dopaminergic treatment of the movement disorders in some of the individuals with *BORCS5* pathogenic variants.

Pathogenic mutations in several components of the HOPS complex, including *VPS11*, *VPS16*, and *VPS41*, which are known to functionally interact with BORC and are involved in autophagosome-lysosome fusion and late-endosome to lysosome maturation (8, 9), have been described as causative for individuals with movement disorders like ataxia and dystonia (39–42). Thus, lysosomal dysfunction underlying these genetic defects might be a unifying factor in the pathogenesis of these movement disorders.

In conclusion, the dual role of BORCS5 in anterograde lysosomal movement and lysosomal function may explain the distinct clinical phenotypes observed in individuals with different *BORCS5* pathogenic variants. Complete BORCS5 LoF leads to severe neurodevelopmental defects and diffuse prenatal NAD, whereas missense variants impair lysosomal activity without disrupting lysosomal distribution, resulting in infantile-onset neurodegeneration. These insights provide a foundation for further research into BORC-mediated lysosomal homeostasis, with potential therapeutic implications for neurodegenerative disorders.

## Methods

### Sex as a biological variable.

Sex was controlled as a biological variable by including in the study fibroblast lines from both sexes.

### Patient ascertainment and clinical and molecular studies.

Comprehensive clinical data were collected from all affected individuals, including detailed phenotypic features, family history, photographs, videos, clinical notes, and brain MRI findings. All brain MRI scans were reviewed and interpreted by an experienced pediatric neuroradiologist.

Whole-exome sequencing and Sanger sequencing were performed independently in different research and clinical laboratories using established protocols (43–45).

Allele frequencies of the identified *BORCS5* variants were evaluated in population databases, including gnomAD v4.1.0 (covering ~800,000 individuals, ~5% of whom are of South Asian descent), the University College London (UCL) Queen Square Genomics Database (*n* ~ 35,000 individuals, enriched for underrepresented populations), and the Igenomix internal database (*n* ~ 65,000 individuals, ~20% of whom are of Arab ancestry).

Skin fibroblasts were obtained from affected individuals from F-I (*n* = 2 lines), F-II (*n* = 1 line), and F-V (*n* = 1 line). Two unrelated fibroblast lines from sex- and age-matched control individuals were also included in the study.

### Culture and transfection of human cell lines.

Cells were maintained at 37°C in a 5% CO_2_ incubator and routinely tested for mycoplasma contamination using PCR-based detection (Venor GeM Mycoplasma Detection Kit; Sigma, MP0025). HEK-293 FT and HeLa cells were cultured in DMEM (Gibco, 11995-065) supplemented with 10% heat-inactivated FBS (HI-FBS; Benchmark, 100-106). Fibroblasts were cultured in DMEM (Gibco, 11995-065) supplemented with 15% HI-FBS (Benchmark, 100-106), 10 U/mL penicillin and 10 μg/mL streptomycin (1% Pen Strep; Gibco, 15140-122). Cells were passaged with trypsin (TrypLE; Invitrogen, 12605-010) for maintenance. Details on cell line generation, cell culture, transfection of plasmids, Western blot analysis, are available in the Supplemental Methods.

### Lysosomal distribution analysis by imaging.

Quantification of LAMP1 distribution in HeLa cells was performed as previously described (46). Briefly, transfected HeLa cells were immunostained and Z-stack cell images were acquired on a Zeiss LSM 900 inverted confocal microscope (Carl Zeiss) using a Plan-Apochromat ×63 objective (numeric aperture = 1.4). The final images were subjected to shell analysis (as shown in the schematic of Figure 5E), excluding cell in which perinuclear lysosome clusters were situated too close to the plasma membrane. Cell outlines were traced in Fiji (https://imagej.net/software/fiji/) using the phalloidin staining as track, and the total fluorescence of LAMP1 signal was measured. The LAMP1 signal intensity was measured in this smaller shell and subtracted from the larger value. The intensity of the LAMP1 signal within the peripheral 2 μm shell was then plotted as percentage of the total cellular LAMP1 signal.

For fibroblasts, lysosomal distribution in confocal images was analyzed by ImageJ/Fiji, with modifications we made to a published protocol (47). Specifically, fibroblast coverslips were co-stained for LAMP1, actin to outline the cell area, and DAPI to define nuclei. Four different regions per coverslip were imaged by confocal microscopy. In each image, nonoverlapping individual fibroblasts were analyzed using the DAPI channel as a reference to define 4 regions of interest per fibroblast, as shown in the schematic of Figure 6F. To this end, we designed 4 concentric rings of 1.5 increments from the nucleus towards the cell periphery (oval shaped rings 1-4). LAMP1 particles were counted for each of the 4 rings, with the number counted for the regions of interest defined by the outermost ring representing 100%.

### Transmission electron microscopy.

Fibroblasts and iNeurons were pelleted by centrifugation at 300–500*g* for 3 minutes at room temperature (RT) in PBS. The cell pellet was fixed with 2.5% glutaraldehyde and 0.1 M cacodylate buffer for 1 hour at RT and then gently dislodged and embedded into 3% UltraPure LMP Agarose (Invitrogen, 16520-100) diluted in water for 1 hour at RT. The CAM/FEI Spirit G2 TEM microscope was used for imaging. Structures were classified according to morphological criteria established in published articles (48–53).

### Assessment of lysosomal GCase activity in fibroblasts.

For fibroblasts, the protocol was adapted from previous studies (54, 55). Fibroblasts were plated at the density of 100,000 cells/well of a 12-well plate for 48 hours. After trypsin dissociation and precipitation at 500*g* for 3 minutes at RT, cells were resuspended in 100 μL of fibroblast medium containing 250 μM PFB-FDGlu substrate (catalog P11947, Invitrogen) for 30 minutes in a 37°C incubator, stopped by the addition of 1 mL ice-cold FACS buffer (DPBS Ca^2+^/Mg^2+^–free; Gibco, 14190-144), with 1 mM EDTA (Invitrogen, 15575-020), 25 mM HEPES (Sigma, H0887), and 5% vol/vol HI-FBS. After 2 washes, cells were placed in 300 μL ice-cold FACS buffer, and the fluorescence signal was measured by flow cytometry using 3C.A1 LSR Fortessa 1 Analyzer.

### Assessment of lysosomal proteolysis in fibroblasts.

Fibroblasts were plated on 12-well plates at a density of 100,000 cells/well. After 72 hours’ conditioning, the medium was replaced by 1 mL of fresh medium containing 25 μg/mL DQ Red BSA (Invitrogen, D12051) or DQ Green BSA (Invitrogen, D12050) for 5.5 hours, at 37°C. After the incubation, the medium was aspirated, followed by incubation with 500 μL trypsin for 5 minutes, at 37°C. The dissociation was stopped by the addition of 1 mL ice-cold FACS buffer, centrifugation at 500*g* for 3 minutes at RT, followed by an additional wash with final resuspension to 300 μL of ice-cold FACS buffer. The fluorescence signal was measured by flow cytometry in 3C.A1 LSR Fortessa 1 Analyzer.

### Generation of iPSCs and neuronal differentiation.

iPSCs were generated from fibroblasts derived from patients F-I:1 and F-1:2, and a control iPSC line was purchased (KOLF2.1J; catalog JIPSC1000, The Jackson Laboratory) to generate isogenic CRISPR/Cas9 *BORCS5* KO (additional information is provided in Supplemental Methods).

iPSCs were differentiated into iNeurons via NGN2 overexpression using published protocols (20, 51, 56). For Lysotracker Red and cathepsin B activity experiments, iNeurons were generated via lentivirus with doxycycline-inducible NGN2 expression cassette (20).

For iNeurons used in the lysosomal GCase activity assay and protein analysis of endolysosome-enriched fractions (lysosomal immunoprecipitation [Lyso-IP], iNeurons were differentiated from iPSCs with stably integrated neurogenin-2 transgene under a doxycycline-inducible promoter (55, 56).

### Endolysosome enrichment from iNeurons (Lyso-IP).

iNeurons (*n* = 6 × 10^6^ per genotype) were transduced at day 14 with a viral construct expressing TMEM192-GFP-3xHA at an MOI of approximately 1, and processed for endolysosome-enrichment (Lyso-IP) at day 21 based on published protocols (21), with modifications (see protocol details in Supplemental Methods).

### Live-cell confocal microscopy of iPSC-derived neurons.

Cortical neurons were incubated with LysoTracker Red DND-99 (L7528; Thermo Fisher Scientific) (50 nM); Magic Red cathepsin B (ICT937; BioRad) (1:2,000); or calcein-AM-488 (20 nM) for 30 minutes in fresh culture medium. After 3 washes in fresh medium, cells were randomly selected for imaging based on calcein-488 staining. To assess GCase activity, iNeurons were cultured in 96-well plates and assayed at day 21, upon incubation with substrate LysoFQ-GBA (57) and calcein AM Red-Orange (Thermo Fisher; 20 nM).

Confocal live-cell images were processed and analyzed using NIS-Elements 5.3 software (Nikon). Photobleaching in time-lapsed images was corrected using intensity equalization over time and further processed using Denoise AI and the 2D deconvolution module (automatic mode). The General Analyzer tool was used for the analysis of Magic Red cathepsin B and LysoTracker Red DND-99 staining. Briefly, rolling ball was performed for background correction, and the Magic Red and LysoTracker signals were thresholded and filtered for all objects between 0.1 and 2 μm. The number of Magic Red– or LysoTracker Red–positive puncta represents the number of puncta divided by cell area, and mean fluorescence intensity was measured per thresholded object. Automated imaging of LysoFQ-GBA fluorescence intensity was obtained using a ×20 ImageXpress high-content imaging system (Molecular Devices) and quantified following established protocols (55).

### Generation of borcs5-ko F0 zebrafish model and rescue experiments.

*borcs5*-ko embryos were generated using the CRISPR-Cas9 system and 3 selected gRNA target sequences (10), with 1 nL of the mix injected into embryos at the 1-cell stage. High-resolution melting analysis and Sanger sequencing were used for genotyping (primer sequences are available upon request).

For rescue and overexpression experiments in zebrafish *borcs5*-ko models, cDNA constructs encoding WT human *BORCS5*, as well as the homozygous missense variants c.284G>A; p.(R95Q) and 296A>C; (p. H99P), were generated. WT BORCS5, R95Q BORCS5, and H99P BORCS5 were subcloned into the pcs2^+^ vector, and mRNA synthesis was carried out using the mMESSAGE mMACHINE SP6 transcription kit (Ambion). Capped and polyadenylated mRNA of WT *BORCS5* and the mutant variants was synthesized in vitro using the mMESSAGE mMACHINE kit (Ambion). Embryos at the 1-cell stage were injected with 1 nL of WT *BORCS5* mRNA or mutant mRNAs (40 ng/μL) co-injected with the Cas9/gRNA mix for functional rescue in *borcs5*-ko embryos. Morphological analysis of zebrafish embryos was performed under a Leica S6E stereoscope.

### Statistics.

Comprehensive information on statistical analyses and post hoc tests applied is provided separately in each figure legend. *P* < 0.05 was considered significant.

### Study approval.

Recruited individuals and/or their legal guardians gave informed consent for their participation. This study received approval from the Review Boards and Bioethics Committees at University College London Hospital (project 06/N076). Informed consent was obtained for all individuals enrolled in the study.

### Data availability.

The data supporting the findings of this study are available within the article and its supplemental materials. Values for all data points in graphs are reported in the Supporting Data Values file. Whole-exome sequencing data are not publicly available, due to privacy or ethical restrictions. Additional information is available upon request.

## Author contributions

NEM, GM, RM, SAP, JSB, KPB, and DK contributed to the conception and design of the study. NEM, GM, RM, RDP, AP, TBF, TA, PS, DC, WJP, FM, DC, SHE, JB, TM, JV, JDOE, LM, MGC, IMW, EJK, MSZ, AS, GZ, ZNAH, EM, SS, LSM, SHCA, GAO, KAT, AAM, M Schwake, M Severino, HH, SAP, JSB, KPB, and DK contributed to the acquisition and analysis of data. NEM, GM, RDP, AP, PS, M Severino, SAP, JSB, and DK contributed to drafting the text and preparing the figures. The sequence of co–first authors was arranged to represent their major contributions to the study, while also recognizing differences in the extent of their involvement in the collaborative effort.

## Conflict of interest

NEM is supported by the Align Science Across Parkinson’s Global Parkinson’s Genetics Program. He is a member of the steering committee of the PD GENEration study, for which he receives an honorarium from the Parkinson’s Foundation. FM is supported by the National Institute for Health and Care Research University College London Hospitals Biomedical Research Centre (BRC) Translational Neuroscience Intermediate Clinical Fellowship (Grant ID: BRC1287/TN/FM/101410), the Edmond J. Safra Movement Disorders Research Career Development Award (grant MJFF-023893), Parkinson’s UK (grant G-2401), the American Parkinson Disease Association (grant 1282403), and the David Pearlman Charitable Foundation. IMW is an employee of and may own stock in GeneDx, LLC. KPB has received grant support from the Edmond J. Safra Movement Disorders Research Career Development Award (grant MJFF-023893), Parkinson’s UK (grant G-2401), Engineering and Physical Sciences Research Council (EPSRC), and the David Pearlman Charitable Foundation. He has received a stipend from the international Parkinson’s Disease and Movement Disorders Society as editor of Movement Disorders Clinical Practice journal and book royalties from Oxford University Press. DK is the founder of Vanqua Bio and Lysosomal Therapeutics Inc and is a venture partner at OrbiMed.

## Funding support

This work is the result of NIH funding, in whole or in part, and is subject to the NIH Public Access Policy. Through acceptance of this federal funding, the NIH has been given a right to make the work publicly available in PubMed Central.

- NIH (grants 1K08NS131581 to NEM and R37 NS096241 and R35 NS122257 to DK).
- NIH intramural program of Eunice Kennedy Shriver National Institute of Child Health and Human Development (project ZIA HD001607 to JSB).
- Parkinson’s Foundation (grant PF-SPE-874858 to NEM).
- Canadian Institutes of Health Research (grant OGB-177940 to SAP).
- Academy of Scientific Research and Technology-Academia/Industry Partnership Program to Leverage Economic Growth, Egypt (grant 9294 to SHE).
- Fonds de Recherche du Québec-Santé doctoral scholarship to AP.
- Deutsche Forschungsgemeinschaft (grants SCHW866/6-1 and 7-1 to M Schwake).

## Supplementary Material

Supplemental data

Unedited blot and gel images

Supporting data values

## Figures and Tables

**Table 1:**
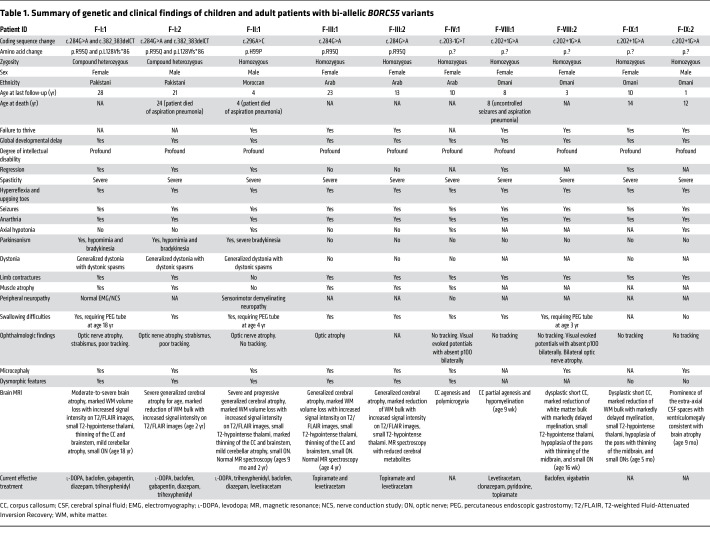
Summary of genetic and clinical findings of children and adult patients with bi-allelic *BORCS5* variants

## Version 1 04/21/2026

In-Press Preview

## Version 2 06/01/2026

Electronic publication
