# Supplementary material for: Pathogenic variants in BORCS5 cause a spectrum of neurodevelopmental and neurodegenerative disorders with lysosomal dysfunction
Source: J Clin Invest. 2026 Apr 21;136(11):e195336. doi: 10.1172/JCI195336 (PMC13221227; doi:10.1172/JCI195336)
Supplement: Supplemental data [file jci-136-195336-s098.pdf]

Supplementary methods

Patient ascertainment and clinical and molecular studies

Comprehensive clinical data were collected from all affected individuals, including detailed phenotypic features, family history, photographs, videos, clinical notes, and brain MRI findings. All brain MRIs were reviewed and interpreted by an experienced pediatric neuroradiologist.

WES and Sanger sequencing were performed independently in different research and clinical laboratories using established protocols (1-3).

In the index family (F-I), WES was carried out on the two affected siblings and both parents. Based on the consanguineous family structure, analysis focused on identifying rare bi-allelic coding and essential splice-site variants. *BORCS5* emerged as the sole candidate gene from this analysis.

To identify additional families with bi-allelic *BORCS5* variants, we adopted a genotype-first approach. Systematic re-analysis and screening of large-scale sequencing datasets were performed across multiple collaborative research networks and data-sharing platforms. These included GeneMatcher (Families F-II, F-IV, F-V), Igenomix (Family F-VI), CENTOGENE (Family F-III), GeneDx (Family F-VII), as well as screening of datasets from the UCL Queen Square Genomics platform, the 100,000 Genomes Project, Solve-RD, Baylor Genetics, Lifera Omics, Genesis, ClinVar, VarSome, and several smaller local and private diagnostic or research laboratories.

Allele frequencies of the identified *BORCS5* variants were evaluated in population databases, including gnomAD v4.1.0 (covering ~800,000 individuals, ~5% of whom are of South Asian descent), the UCL Queen Square Genomics Database (~35,000 individuals, enriched for underrepresented populations), and the Igenomix internal database (~65,000 individuals, ~20% of whom are of Arab ancestry).

Skin fibroblasts were obtained from affected subjects from F-I (two lines), F-II (one line), and F-V (one line). Two unrelated fibroblast lines from gender and age-matched control subjects were also included in the study.

Culture and transfection of human cell lines

Cells were maintained at 37°C in a 5% CO_2 incubator and routinely tested for mycoplasma contamination using PCR-based detection (Venor GeM Mycoplasma Detection Kit (Sigma, MP0025). HEK-293 FT and HeLa cells were cultured in Dulbecco's modified Eagle's Medium (DMEM; Gibco, 11995-065) supplemented with 10% heat-inactivated fetal bovine serum (HI-FBS; Benchmark, 100-106). Fibroblasts were cultured in Dulbecco's modified Eagle's Medium (DMEM; Gibco, 11995-065) supplemented with 15% heat-inactivated fetal bovine serum (HI-FBS; Benchmark, 100-106), 10 U/ml penicillin and 10 µg/ml streptomycin (1% Pen Strep; Gibco, 15140-122). Cells were passaged with trypsin (TrypLE; Invitrogen, 12605-010) for maintenance.

HeLa and HEK-293T cells were transiently transfected with 0.8 µg plasmid DNA using 2 µl Lipofectamine 2000 (Invitrogen), according to the manufacturer's instructions. Approximately 24 h after transfection, HEK-293T cells were harvested and HeLa cells were replated onto 12-mm coverslips coated with collagen. HeLa cells were then cultured for an additional 24 h before fixation and immunofluorescence labelling.

HeLa BORCS5-KO cell lines stably expressing BORCS5-3xHA or LAMP1-KBS-GFP: BORCS5-3xHA plasmid was generated by Gibson cloning using BORCS5-GFP and a lentiviral vector containing 3xHA repeats. Lentiviral particles for BORCS5-3xHA and LAMP1-KBS were obtained from HEK 293T cells, using VSVG and DR8.91 plasmids, and used to transduce HeLa BORCS5 KO cells (together with 8mg.mL polybrene, cat 7711, Tocris). Hygromycin-resistant clones (300mg.mL, cat 10687010, Thermofisher) were selected and BORCS5-3xHA or LAMP1-KBS expression confirmed.

HeLa BORCS5 CRISPR/Cas9 knockout generation

Briefly, two 20-base pair (bp) targeting sequences (GCTCAACAGCATGCTGCCCG and AGCAGATCCAGAAAGTGAAC) were synthesized (Eurofins) and introduced separately into the px330 plasmid (Addgene). HeLa cells were co-transfected with both plasmids and re-seeded after 72 h to allow single colony formation. After 12 days, genomic DNA was extracted from individual colonies, and cleavage of the target sequence was tested by PCR using a pair of primers (ATCTGCGGGACTGTGTCCCT and CAGATTTTCATGCCAGCCGG), which produced a 99-

bp smaller band in KO cells relative to WT cells. The KO was confirmed by Sanger sequencing and immunoblotting.

BORC assembly studies using GFPtrap precipitation

For GFP precipitation, cells were transfected with cDNA of wild-type or mutant BORCS5-GFP, using GFP alone as control. After 24 hours of expression, cells were harvested and subsequently lysed by sonication in 10mM Tris-CL pH 7.5, 150 mM NaCl, 0.5 mM EDTA, 1% Triton X 100, 10%glycerol) supplemented with protease inhibitor cocktail (Roche). Cell debris was removed by centrifugation and the supernatant was subjected to immunoprecipitation. A fraction of the supernatant was kept as an input sample. ChromoTek GFP-Trap Agarose beads (Proteintech, USA) blocked with 5% BSA in lysis buffer were incubated with residual supernatant for 2 hours at 4°C. Beads were collected by centrifugation at 800xg and 4°C and washed five times with 500 µl lysis buffer. Precipitates were eluted with Laemmli buffer (Bio-Rad) containing β-mercaptoethanol at 55°C for 30 min. The immunoprecipitated samples and inputs were analyzed by immunoblotting.

Protein extraction and western blot analysis

HEK-293T and fibroblasts were washed with PBS and protein was extracted in RIPA buffer (Boston BioProducts, BP-115-5x; 50 mM Tris-HCl, pH 7.4, 150 mM NaCl, 1% Nonidet P-40 substitute, 0.5% Na-Deoxycholate, 0.1% sodium dodecyl sulfate), supplemented with protease (Roche, #11836170001) inhibitors. Depending on the protein marker of interest, 20 to 50 µg of total protein were electrophoresed using Tris-Glycine gradient gels (Novex™ 4-12% Tris-Glycine Mini Gels, WedgeWell™ format, 12-well, XP04122BOX; 15-well- WedgeWell, XP04125BOX; Novex™ WedgeWell™ 4-20% Tris-Glycine Mini Gels, 12-well, XP04202BOX; 15-well-WedgeWell, XP04205BOX, Thermo Fisher Scientific), semi-dry transferred onto PVDF membranes (Trans-Blot Turbo System, 1704275, BioRad) and incubated for 1 h at room temperature with blocking buffer (TBS-T; Tris-buffered saline with 0.1% Tween-20 (Applichem, A1389,0500) supplemented with 1% w/v bovine serum albumin (Sigma-Aldrich, A9647). The primary antibody was diluted in a blocking agent and incubated overnight at 4°C with mild

shaking, followed by TBS-T washes, and incubation with appropriate secondary antibodies (also diluted in blocking buffer). Chemiluminescence was visualized and analyzed using the Chemidoc Software (BioRad).

Lysosomal distribution and LC3 levels analysis by imaging in HeLa and fibroblasts

Quantification of LAMP1 distribution in HeLa cells was performed as previously described (Williamson et al., 2022 PMID: 35819772). Briefly, HeLa cells grown on 6-well plates were transiently transfected with 0.8 µg of each plasmid DNA using 2 µl Lipofectamine 2000 (Invitrogen), according to the manufacturer's instructions. Approximately 24 h after transfection, cells were replated on collagen-coated coverslips in 24-well plates at 40,000 cells per well. Cells were then cultured for an additional 24 h to allow rescue of the BORC phenotype. Cells were then fixed in 4% w/v paraformaldehyde (Electron Microscopy Sciences) in PBS for 20 min, permeabilized and blocked with 0.1% w/v saponin, 1% w/v BSA (Gold Bio) in PBS for 20 min, and sequentially incubated with primary and secondary antibodies (mouse anti-LAMP1 (H4A3, DSHB, IF 1:500), chicken anti-GFP (A10262, Thermo Fisher, IF 1:500), Alexa Fluor 555-conjugated donkey anti-mouse IgG (A-31570, Thermo Scientific, IF 1:1,000), Alexa Fluor 488-conjugated Goat anti-Chicken IgY (H+L) (A-11039, Thermo Scientific, IF 1:1,000), Alexa FluorTM 647-conjugated phalloidin (A22287, Thermo Scientific, IF 1:50) diluted in 0.1% w/v saponin, 1% w/v BSA in PBS for 30 min at 37°C. Coverslips were washed three times in PBS and mounted on glass slides using Fluoromount-G (Electron Microscopy Sciences) with DAPI. Z-stack cell images were acquired on a Zeiss LSM 900 inverted confocal microscope (Carl Zeiss) using a Plan-Apochromat 63X objective (NA=1.4). Maximum intensity projections and final composite images were created using ImageJ/Fiji (https://fiji.sc/). The final images were subjected to shell analysis (as shown in the schematic of Fig. 5C). Briefly, cells exhibiting morphologies where perinuclear clusters of lysosomes were situated too close to the plasma membrane were excluded from analysis. Cell outlines were traced in Fiji (https://imagej.net/software/fiji/) using the phalloidin staining as track, and the total fluorescence of LAMP1 signal was measured. The cell outline was then shrunk by 2 µm using the "enlarge" function in Fiji. The LAMP1 signal intensity was measured in this smaller shell and subtracted from the larger value. The intensity of LAMP1 signal within the peripheral 2-µm shell was then plotted as percentage of total cellular LAMP1

signal. For statistical analysis, we performed a one-way analysis of variance (ANOVA), followed by multiple comparisons using Dunnett's test. All statistical analyses were conducted using Prism version 9 (GraphPad Software). For the LC3B quantification cells were plated, stained (rabbit anti-LC3 (3868, Cell Signaling, IF 1:200), Alexa Fluor 555-conjugated goat anti-rabbit IgG (A-21428, Thermo Scientific, IF 1:1,000), and images were acquired as described earlier in this paragraph. LC3B particles were counted for each cell using the "Analyze particles" function of Fiji.

For fibroblasts, lysosomal distribution in confocal images was analyzed by ImageJ/Fiji, with modifications in a published protocol (4). Specifically, fibroblast coverslips were co-stained for LAMP1, actin to outline the cell area, and DAPI to define nuclei. Four different regions per coverslip were imaged by confocal microscopy. In each image, non-overlapping individual fibroblasts were analyzed using the DAPI channel as reference to define four regions of interest (ROI) per fibroblast, as shown in the schematic of Fig. 5F. The first ROI was the outline of the nucleus (oval-shaped ring number 1) and an additional three concentric rings of 1.5 increments towards the cell periphery (oval-shaped rings 2 to 4) were designed. Next, using the LAMP1 channel (type 8-bit) a threshold (Image > Adjust > Threshold) and a mask of LAMP1 particles were created (Process > Binary > Convert to mask), and overlapping particles were distinguished (Process > Binary > Watershed). Finally, LAMP 1 particles were counted for each of the 4 rings (Analyze > Analyze particles), with the number counted for the ROI defined by the outermost ring representing 100%. The data was expressed as the % of total $LAMP1^{+}$ vesicles present within each ring, thus representing the dispersion of endolysosomes in the perinuclear region (nucleus proximal rings 1 and 2) towards the cell periphery (between nucleus distal rings 3 and 4).

Exosome/extracellular vesicle isolation by ultracentrifugation

All centrifugations as well as the 0.20 µm filtration were performed at room temperature, while the ultracentrifugation was performed at 4°C. Fibroblasts were cultured in a 15-cm dish until 80% confluency. Culture medium was replaced with 18 mL DMEM (Gibco, 11995-065) supplemented with 10% exosome-depleted FBS (Gibco, A27208-03) and conditioned for 24 h. The conditioned medium was pre-cleared by sequential centrifugation at 300 × g, 5 min, then at 3000 × g, 10 min, and subsequently filtered through a 28-µm syringe 0.2-µm filter (Corning, 431219). Finally, 4 mL of the pre-cleared medium (corresponding to approximately $8x10^{6}$ cells) was ultracentrifuged

(open-top thin-wall ultra-clear tube, 11 × 60 mm; Beckman Coulter, 344062) for 90 min, using the SW60 Ti rotor (Beckman Coulter). The final pellet (EV fraction) was resuspended in 30 µL of sample buffer (32.4 mM Tris HCl pH 6.8, 13.15% glycerol, 1.05% SDS).

Generation of induced pluripotent stem cells

Two patient-derived fibroblast lines from subjects F-I:1 and F-1:2 were reprogrammed by the Stem Cell Core Facility at Northwestern University to generate induced pluripotent stem cells (iPSCs). For additional information regarding iPSC generation refer to supplemental materials. iPSC lines were shown to express the pluripotency markers Nanog, Oct4, SSEA-4, and Tra-1–81 through immunofluorescence analysis, and g-band karyotype analysis was performed by Cell Line Genetics (https://www.clgenetics.com/). Additionally, an iPSC isogenic BORCS5 knock-out (BORCS5-KO) line was generated using the CRISPR/Cas9 system. Optimal CRISPR guides were chosen using the CRISPR design tool (5) to target *BORCS5* exon 2 and introduce a bi-allelic frameshift variant which would result in a complete loss of BORCS5. Guide RNAs were cloned into a plasmid expressing the Cas9 D10A nickase pSpCas9n(BB)-2A-GFP (PX461; Addgene # 48140) and Sanger sequenced to ensure proper cloning. iPSC colonies grown on a 10 cm dish were dissociated using Accutase and 5 million cells were transduced with 3 µg CRISPR guides using the Neon transfection system (Thermo Fisher). Transduced cells were then plated using mTeSR with 10 µM ROCK inhibitor. After 48 h, GFP-positive cells were sorted and plated at clonal density (10,000 cells/plate) on Matrigel-coated 10 cm dishes. Individual colonies were manually passaged and plated in 48 well plates. Clones were grown to confluence and passaged using Accutase. About 15% of cells were replated and the remaining were used for Sanger sequencing reactions. Crude genomic DNA was obtained using Viagen extraction reagents. Corrected clones were expanded, resequenced, and submitted for g-band karyotype analysis (Cell Line Genetics). The presence of pluripotency markers and a normal karyotype was confirmed in all four lines. The introduction of bi-allelic frameshift variants in BORCS5 exon 2 was demonstrated by Sanger sequencing and the complete KO of BORCS5 was confirmed by Western Blot analysis.

Live-cell confocal microscopy of iPSC-derived neurons

Confocal live-cell imaging was performed using a Nikon W1 Spinning Disk microscope with a 100×-oil objective (TIRF 100x 1.49 NA; Nikon Plan Apo). iNeurons were cultured and transduced or stained as described above and imaged in four-chamber glass-bottom dishes (D35C4-20-1.5-N; Cellvis) in a temperature-controlled (37°C) and a humidified chamber with 5% CO_2. Images were acquired in single-camera mode with 500-ms exposure time. Cells were imaged at 1 frame every 2 s for 3 min total. Live cells were imaged in a temperature-controlled chamber (37°C) at 5% CO_2 at one frame every 2–3 s. Dual-color videos were acquired as consecutive green-red images and tricolor videos were acquired as consecutive green-red-blue images. For live-cell imaging microscopy, cortical neurons were incubated with LysoTracker Red DND-99 (L7528; Thermo Fisher Scientific) (50 nM), Magic Red cathepsin B (ICT937; BioRad) (1:2,000), or Calcein AM-488 (20 nM) for 30 min in fresh culture media. Cells were imaged after three quick washes in fresh culturing media. Imaged cells were randomly selected based on Calcein-488 staining to achieve blinding of the investigator.

To measure lysosomal GCase activity in iNeurons, cells were plated on coated 96-well plates. At 21 days, iNeurons were incubated with LysoFQ-GBA(6) and Calcein AM Red-Orange (Thermofisher; 20 nM). Automated imaging of LysoFQ-GBA fluorescence intensity was obtained using a 20× ImageXpress high-content imaging system (Molecular Devices) and was quantified using CellProfiler (7) following established protocols (8).

Immuno-precipitation of TMEM192+ neuronal fractions (Lyso-IP)

Enrichment of endolysosomes was based on published protocols with modifications (9). Briefly, $6x10^6$ iNeurons per genotype were transduced at day 14 with a viral construct expressing TMEM192-GFP-3xHA (TMEM192 from Addgene plasmid #102930, EGFP, and 3x HA tag were subcloned together into Addgene plasmid #170995, replacing the coding sequence between the BamHI and EcoRV restriction sites) at an MOI ~ 1, with half media change, followed by full medium change one day post transduction. The iNeurons were harvested and processed for endolysosome-enrichment (Lyso-IP) at day 21. During harvesting, medium was aspirated and intact neuronal sheets were collected in ice-cold PBS followed by centrifugation for 500xg for 3 min, 4^oC. The neuronal pellet was resuspended in 500 µL KPBS pH 7.25 (136 mM KCl, 10 mM

KH2PO4) supplemented with protease inhibitors (Roche, 11836170001) and immediately passed through an ice-cold 23G syringe seven times. The suspension was centrifuged at 1000 xg for 3 min at 4°C for 3 min, and 5% of the supernatant was kept as input. The remaining supernatant was mixed with 60-100 µL Anti-HA Magnetic Beads (Pierce, Cat. no. 88837) and placed on rotation for 40 min at 4°C. Subsequently, beads were placed on a magnetic stand, followed by three washes with 500 µL KPBS supplemented with protease inhibitors. After the final wash, the endolysosomes were eluted from the beads by adding 40-60 µL sample buffer (32.4mM Tris HCl pH 6.8, 13.15% glycerol, 1.05% SDS) and boiled at 65°C for 20 min or 95°C for 5 min. Protein was quantified by Bicinchoninic Acid (BCA) Assay, and 3-6 µg input or Lyso-IP were analyzed by western blot. For normalization purposes, following protein transfer to membrane, the remaining protein bands on the gel were stained using for 1 h by Coomassie G-250 stain (SimplyBlue™ SafeStain, Cat. no. LC6060), at RT, followed by 3 rinses with tap water and overnight de-staining. The protein ladder was visualized and quantified using Odyssey® Imager (LICORbio).

Zebrafish husbandry

Adult wild-type (WT) zebrafish (*Danio rerio*; AB strain) were maintained at 28°C under a 12 h light/12 h dark cycle, according to the Westerfield zebrafish book. All zebrafish in this study used for cross-breeding were housed in groups and fed twice daily with a standardized diet of Skretting® Gemma Micro starting as of 5 days post-fertilization (dpf). Embryos were maintained at 28.5°C, collected, and staged as previously described.(10) All experiments were conducted in accordance with the guidelines of the Canadian Council for Animal Care and approved by the Institutional Animal Care and Use Committee of INRS-LNBE.

Zebrafish behavioral assays

Larvae (5 dpf) were transferred individually into a 96-well plate containing 200 µl of E3 medium. The well plate was placed in the Daniovision® recording chamber (Noldus) for 30 min before the start of the experiment. Locomotor activity for 2 hours was recorded using a Basler GenIcam camera. Analysis was performed using the Ethovision XT 12 software (Noldus) to quantify the cumulative distance swam and swim velocity.

Zebrafish phalloidin staining

To visualize muscles using phalloidin staining, 3 dpf embryos were fixed overnight at 4 °C in 4% paraformaldehyde (PFA). Phalloidin staining was performed following the previously described protocol.(11) Analyses were conducted on Z-stack images acquired with a Zeiss LSM 780 confocal microscope.

Zebrafish motor axon visualization

Immunohistochemical analyses were performed on 3 dpf zebrafish embryos to visualize motor neuron axonal projections. The embryos were fixed overnight at 4 °C in Dent's fixative (20% DMSO and 80% methanol). Phalloidin staining was carried out as previously described.(11) Analyses were conducted on Z-stack images acquired using a Zeiss LSM 780 confocal microscope.

Zebrafish H&E brain staining

For brain section staining, hematoxylin and eosin (H&E) staining was performed on 5-µm paraffin-embedded brain sections of 3 dpf larvae. Sections were post-fixed in 10% formalin (Chaptec) for 5 minutes and subsequently rinsed with tap water. Tissue sections were subjected to hematoxylin staining (StatLab) for a duration of 4 minutes, followed by differentiation in an acid-alcohol solution and thorough rinsing with tap water. To enhance nuclear contrast, sections were subsequently immersed in a saturated lithium carbonate solution for 10 seconds and rinsed again with tap water. Counterstaining was performed using Eosin Y (StatLab) for 2 minutes. Finally, sections were mounted under coverslips using Permount mounting medium (Thermo Fisher) to ensure long-term preservation and optical clarity.

Zebrafish p-MAPK/ERK staining

PTZ treatments were performed in the dark for 15 or 30 minutes on larvae (4 dpf), then rapidly fixed in 4% PFA and kept at 4°C overnight. Following fixation, larvae were extensively washed with phosphate-buffered saline (PBS) containing 0.1% Tween 20 and subsequently incubated in

100% acetone for 15 minutes. The acetone was then removed by washing with PBS containing 0.3% Triton X-100, followed by PBS-DT (PBS supplemented with 1% bovine serum albumin [BSA], 1% dimethyl sulfoxide [DMSO], and 1% Triton X-100). To minimize nonspecific binding, samples were blocked for 1 hour in PBS-DT supplemented with 5% normal goat serum. Larvae were then incubated overnight at 4°C with the primary antibody against phospho-MAPK1/ERK2-MAPK3/ERK1 (1:500; Cell Signaling Technology, 4370S). After multiple washes in PBS-DT, samples were incubated overnight at 4°C with the Alexa Fluor 488-conjugated goat anti-rabbit secondary antibody (1:1000; Invitrogen, A-11008). The larvae heads were subsequently mounted ventrally on slides using Fluoromount (ThermoFisher) and imaged using a Zeiss LSM 780 confocal microscope. Image processing and analysis were performed using Fiji (ImageJ).

Zebrafish tyrosine hydroxylase staining

Visualization of dopaminergic neurons was performed on 3 dpf larvae. Briefly, animals were fixed in 4% paraformaldehyde overnight at 4°C. After fixation, the larvae were rinsed several times for 1 h with PBS-Tween (0.1%) and then incubated in PBS-Tween (1%) for 2 h. Larvae were incubated in freshly blocking solution (2% normal goat serum, 1% BSA, 1% DMSO, 1% Triton-X in PBS). Then incubated in prepared blocking solution containing primary antibody TH (aTH, 1:200, Developmental Studies Hybridoma Bank) overnight at 4°C. The primary antibody was washed several times for 1 h with PBS-Tween (0.1%) and larvae were incubated in blocking solution containing an Alexa-Fluor-488-conjugated secondary antibody (1:1000, A-21042, Invitrogen) overnight at 4°C. The following day, the larvae were washed several times with PBS-Tween (0.1%) and mounted on a glass slide in Fluoromount (ThermoFisher). Slides were blinded for Z-stack imaging with a Zeiss LSM780 confocal microscope (Carl Zeiss). The images were then processed with ZEN software (Carl Zeiss).

Antibody list

Antibody	Species	Company	Cat. no.	Dilution	Method
Alexa Flour-488 donkey anti-rabbit	Rabbit	Thermo Fisher	A-21206	1 to 500	ICC
Alexa Flour-647 goat anti-mouse	Mouse	Thermo Fisher	A-21235	1 to 500	ICC
Alexa Fluor 488-conjugated Goat anti-Chicken IgY (H+L)	Mouse	Thermo Fisher	A-11039	1 to 1000	ICC
Alexa Fluor 555-conjugated donkey anti-mouse IgG	Mouse	Thermo Fisher	A-31570	1 to 1000	ICC
Alexa Fluor 555-conjugated goat anti-rabbit IgG	Rabbit	Thermo Fisher	A-21428	1 to 1000	ICC
Alexa Fluor 555-conjugated goat anti-rabbit IgG	Rabbit	Thermo Fisher	A22287	1 to 500	ICC
Alix	Rabbit	Proteintech	12422-1-AP	1 to 500	WB
BORCS5	Rabbit	Proteintech	17169-1-AP	1 to 1000	WB
BORCS7	Rabbit	Abnova	PAB23142	1 to 1000	WB
CTSB	Goat	Novus Biologicals	AF953-SP	1 to 1000	WB
Flotillin-1 [D2V7J]	Rabbit	Cell Signaling	18634T	1 to 500	WB
GAPDH	Mouse	Millipore	MAB374	1 to 4,000	WB

GCase	Rabbit	Sigma	G4171	1 to 500	WB
GFP	Chicken	Thermo Fisher	A10262	1 to 500	ICC
LAMP1	Mouse	Santa cruz	sc-20011	1 to 1000	WB and ICC
LAMP1 (H4A3)	Mouse	DSHB	H4A3	1 to 500	ICC
LAMP2	Mouse	Developmental Studies Hybridoma Bank (DSHB)	H4B4	1 to 500	WB and ICC
LC3	Rabbit	Cell Signaling	3868	1 to 200	ICC
LC3A	Rabbit	Novus Biologicals	NB100-2331	1 to 1000	WB
LIMP-2 [EPR12080]	Rabbit	Abcam	ab176317	1 to 1000	WB
Peroxidase AffiniPure Goat Anti-Mouse IgG (H+L)	Mouse	Jackson ImmunoResearch	115-035-146	1 to 5,000	WB
Peroxidase-AffiniPure Goat Anti-Rabbit IgG (H+L)	Rabbit	Jackson ImmunoResearch	111-035-144	1 to 5,000	WB
SNAPIN	Rabbit	Synaptic Systems	148 102	1 to 1000	WB
SQSTM1	Mouse	Abcam	ab56416	1 to 1000	WB
TUBBA	Rat	Pierce	MA1-80189	1 to 10,000	WB

Clinical case reports

Family F-I

Case F-I:1 is a 30-year-old female born to non-consanguineous parents of Pakistani origin. She had normal prenatal, birth, and early postnatal history. At the age of 8 months, she started developing increased muscle tone. At around the age of 1 year, she was diagnosed with epilepsy after being noticed with fluttering eye movements, which were however later interpreted as oculogyric crises. She has never been able to walk. By the age of 5-6 years, she was able to babble, but her speech then deteriorated, and she became anarthric. She has never been continent. Since the age of 18 years, she has experienced dystonic spasms characterized by arching of the back, neck hyperextension, and limb extension, which have improved after L-DOPA initiation. L-DOPA has also reduced the frequency of oculogyric crises. She has been fed via percutaneous endoscopic gastrostomy (PEG) since the age of 19 years when she presented with significant weight loss and an episode of aspiration pneumonia.

On examination (age 28), she was anarthric and wheelchair bound. She presented with facial dysmorphic features, including a short forehead, strabismus, and gaze-evoked nystagmus. She had severe bruxism. There was generalized dystonia with severe opisthotonus as well as limb rigidity. She had brisk reflexes in the upper limbs, while reflexes were sluggish in the lower limbs. She had acral contractures.

Extensive investigations included normal cerebrospinal fluid (CSF) dopamine pathway metabolites, plasma amino acids, and organic urinary acids. No acanthocytes were detected on a peripheral blood smear. The levels of leukocyte beta-galactosidase and plasma beta-glucuronidase activity were higher than normal. An electroencephalogram (EEG) showed irregular 6-7 Hz and 5-10 μV activity over the posterior region, rare 9 Hz and 10 μV activity over the posterior region, widespread low amplitude fast beta activity at 14-15 Hz and less than 5 μV, some 3-5Hz and 5uV activity over the frontocentral region, without epileptic activity. A brain magnetic resonance imaging (MRI) performed at 18 years of age revealed moderate-to-severe brain atrophy associated with marked reduction of white matter (WM) bulk, thinning of the corpus callosum and brainstem, ventricular enlargement, and diffuse white matter increased signal intensity on T2/FLAIR images, consistent with reduced/incomplete myelination. The thalami were small and hypointense on T2-weighted images. Mild cerebellar atrophy with prevalent involvement of the lateral portions of the

cerebellar hemispheres was also noted. Finally, the optic nerves and chiasm were slightly thinned. Electroretinogram (ERG) and flash visual evoked potentials (VEP) were reported to be normal during childhood, thus likely excluding retinal and optic nerve involvement. At age 26, X-rays pelvis revealed bilateral hip subluxation and left hip arthritis, ultrasound (US) abdomen did not detect signs of organomegaly, and nerve conduction study/electromyography (NCS/EMG) ruled out peripheral large fiber neuropathy or myopathic features of the right tibialis anterior muscle.

Her current treatment consists of L-DOPA/Carbidopa 50/12.5 mg three times a day, Baclofen 20 mg three times a day, Gabapentin 300 mg three times a day, Diazepam 2.5 mg three times a day, Trihexyphenidyl 3 mg three times a day, Paracetamol 400 mg twice a day, Buprenorphine patch and laxatives.

The proband's younger brother (case F-I:2) had a normal perinatal history and was first noticed to be different from other infants at the age of 8 months. He had global developmental delay. He managed to stand without support at the age of 1.5 years and to babble. At around the age of 2 years, he developed epilepsy, and his speech started to regress. His brain magnetic resonance imaging (MRI performed at 2 years of age) revealed severe generalized cerebral atrophy for age, marked reduction of WM bulk with increased signal intensity on T2/FLAIR images. At age 11, he was still able to stand with support, hold a cup, and drink. At around the age of 12 or 13 years, he developed problems with posture. Because of hip dislocation, he underwent surgery with insertion of a metal prothesis. Since then, he developed generalized dystonia with superimposed dystonic spasms, which responded to L-DOPA and Trihexyphenidyl.

At the age 18 years, his neurological examination revealed dysmorphic features, including short stature, strabismus, short forehead, and scoliosis. He was anarthric, PEG-fed and wheelchair bound. There was upgaze restriction, severe generalized dystonia with intermittent superimposed extensor spasms, and hyperreflexia.

Metabolic screening, CSF analysis (including pterins and neurotransmitter metabolites), serum ceruloplasmin, plasma amino acids, and lysosomal enzymes were unremarkable.

His treatment included L-DOPA/Carbidopa 100/50 mg three times a day, Baclofen 10 mg three times a day, Trihexyphenidyl 10 mg three times a day, and Gabapentin 700 mg three times a day.

He died from pneumonia at the age of 23 years.

Family F-II

Patient F-II:1 of Moroccan descent was evaluated due to persistent irritability since birth, progressive neurodevelopmental impairment, and severe movement disorders. Symptoms worsened around four months of age, with increasing rigidity, poor visual attention, and feeding difficulties. The primary concerns were refractory hypertonia/dystonia and failure to achieve developmental milestones.

The child was born at term (39+4 weeks of gestation) following an uneventful pregnancy. Delivery was forceps-assisted due to prolonged labor, with normal Apgar (Appearance, Pulse, Grimace, Activity and Respiration) scores (9/10/10). Birth weight was 2720 g (6th percentile), length at birth was 47.5 cm (6th percentile), and head circumference at birth was 34 cm (30th percentile). Neonatal jaundice required phototherapy, but no other immediate complications were reported. However, from the first weeks of life, parents noted excessive irritability, feeding difficulties, and poor visual tracking. Early developmental concerns included lack of head control, absence of social smiling, and progressive hypertonia.

On examination, the child presented with progressive microcephaly, mydriasis with sluggish pupillary responses, and lateral conjugate eye movements without clear fixation. Severe generalized hypertonia/dystonia was observed, with significant extensor posturing, particularly in response to stimuli, but no clear choreiform movements at rest. Deep tendon reflexes were brisk, with sustained clonus in the lower limbs. The child had a weak cry, oral motor dysfunction, frequent vomiting, and required nasogastric feeding, later transitioning to a gastrostomy.

Brain MRI performed at 9 months of age revealed reduced white matter volume with delayed myelination, predominantly supratentorial, associated with widening of the cerebral subarachnoid spaces and enlarged lateral ventricles. The corpus callosum was thin. The thalami appeared small and slightly dark on T2-weighted images. Small hemosiderin deposits were identified in the right caudothalamic groove. Optic nerve and corpus callosum thinning were also noted.MR spectroscopy performed at the level of the right parietal white matter was normal for the age. Follow-up brain MRI performed at 2 years of age demonstrated progression of the cerebral atrophy in association with mild cerebellar atrophy. Both the corpus callosum and brainstem were smaller, as well as the optic nerves. The myelination process did not improve.

Extensive metabolic and mitochondrial laboratory studies were unremarkable. Video EEG showed moderate multifocal epileptiform abnormalities with diffuse distribution in both hemispheres. A subsequent video EEG recorded a tonic motor seizure, likely of focal onset with expression in the right hemisphere.

EMG revealed prolonged latencies and conduction velocities within the demyelinating range for the patient's age, with reduced amplitudes in the motor nerve studies of the median, peroneal, and posterior tibial nerves. Sensory nerve conduction studies of the plantar tibial and right median nerves showed absent potentials. EMG of the tibialis anterior muscle demonstrated a normal interference pattern, suggestive of a motor-sensory demyelinating neuropathy. VEP were poorly structured for the patient's age, while the ERG was normal. Brainstem Auditory Evoked Potentials (BAEP) was also within normal limits. Fundoscopic examination revealed optic disc pallor, predominantly temporal. The echocardiogram was normal.

The initial clinical presentation with severe spasticity/dystonia and the neuroimaging findings suggested Krabbe disease as a potential diagnosis. Genetic testing initially included an array-Comparative Genomic Hybridization (CGH), which was normal, and clinical exome sequencing, which identified a *THAP1* (NM_018105.2): c.433C>T; p.Gln145* variant inherited from the healthy father. However, based on the phenotype and clinical evolution, this was not considered causative of the disease. Subsequent singleton WES identified a *BORCS5* (NM_058169.6): c.296A>C; p.His99Pro variant in homozygosity, confirmed by Sanger sequencing to be inherited from both parents. The variant was absent in the healthy sibling.

The patient received symptomatic treatment. Perampanel, levetiracetam, and valproic acid were introduced to control seizures. L-DOPA, trihexyphenidyl, baclofen, and diazepam were administered for hypertonia and spasticity, with limited benefit. Scopolamine patches were used to reduce excessive secretions. Supportive care included gastrostomy feeding, respiratory physiotherapy, and close monitoring for complications.

Despite medical management, the child exhibited progressive neurological deterioration, with increasing rigidity, reduced responsiveness, and worsening autonomic dysfunction. Episodes of unexplained dystonic posturing became more frequent. Recurrent respiratory infections and gastrointestinal complications led to multiple hospitalizations. Over time, the child developed severe bradycardia and signs of brainstem dysfunction. The patient ultimately passed away

following a progressive decline in neurological and systemic function at 3 years and 10 months of age.

Family F-III

Subject F-III:2 is a 13-year-old girl who has been evaluated for a history of global developmental delay and seizures. She had her first attack of convulsion at the age of 8 months after which she was noticed to become less attentive than before and then has been showing neurodevelopmental delay. She had uneventful prenatal and neonatal history and was discharged on the 2nd day of life. She has had severe motor disability, requiring a wheelchair with minimal use of hands. She never attained the ability to walk. She also has cognitive delay. Her language is severely impaired, she was only capable of imitating sounds.

Her parents are consanguineous. They have one older sister with the same condition.

Her growth parameters are below the 3rd percentiles. She is not dysmorphic. Cranial nerves examination is normal. She has spasticity with brisk tendon reflexes and no focal neurological signs. Chest, cardiovascular and abdomen examination is unremarkable.

Semiology of seizures showed that she has been having 2 types of convulsions: (i) generalized tonic clonic occurring almost monthly; (ii) episodes of staring occurring every 2-3 days. EEG was severely abnormal with abundant epileptiform abnormalities originating from multiple regions but mainly from the right frontotemporal region and associated with a continuous slow activity from the same region, suggestive of a possible underlying cortical dysfunction. She has been on topiramate and levetiracetam.

Brain MRI at the age of 4 years showed generalized cerebral atrophy, marked WM volume loss with increased signal intensity on T2/FLAIR images, small T2-hypointense thalami, thinning of the CC and brainstem, and small ON Brain MRI spectroscopy was normal.

Her similarly affected sister (F-III:1) is 20-year-old who presented with her first attack of convulsion at the age of 8 months. She had an uneventful prenatal and neonatal history. She was noticed to have severe motor disability. She never attained the ability to sit unsupported. She had cognitive delay, and her language was severely impaired, she was never capable of saying words.

Investigations included plasma acylcarnitine, urine organic acids, carbohydrate-deficient transferrin, CGH array, and clinical WES which were all unremarkable.

Her growth parameters were below the 3rd percentiles. She was not dysmorphic. Cranial nerves examination was normal. She had spasticity with brisk tendon reflexes and no focal neurological signs. Chest, cardiovascular and abdomen examination was unremarkable.

EEG showed (i) sharp waves independently bitemporal maximum right mid posterior temporal; (ii) continuous slow activity, right temporal and generalized; (iii) intermittent slow activity independent bitemporal, maximum right; (iv) background slow activity. She was on topiramate and levetiracetam.

Brain MRI revealed severe cerebral atrophy with WM volume loss and marked thinning of the corpus callosum. Both thalami were small and T2- hypointense as compared to the basal ganglia. MR spectroscopy showed reduction of all cerebral metabolites without lactate peaks.

Investigations included plasma ammonia, serum lactate, plasma acylcarnitine, serum biotinidase, carbohydrate-deficient transferrin, urine for creatine disorders, SNP array, and clinical whole exome sequencing which were all normal.

In family F-III, research reanalysis of exome sequencing performed in the proband identified the variant NM_058169.4: c.284G>A; p.(R95Q), in the homozygous state, and Sanger sequencing confirmed that the similarly affected sister also carried the same variant and both parents were heterozygous.

Family F-IV

This patient is a 10-year-old female born to a 36-year-old healthy mother following an uneventful pregnancy.

She was delivered at full term (38 weeks) via spontaneous vaginal delivery. Her birth weight was 3 kg (25th percentile), and her head circumference was 36 cm (75th percentile). There was no history of perinatal or postnatal complications.

She first presented with polymorphic seizures at the age of 2 months, which lasted for a few seconds to a maximum of one minute. By 6 months of age, she developed infantile spasms, and her initial EEG was consistent with hypsarrhythmia.

She was started on vigabatrin; however, despite reaching optimal doses, she continued to experience frequent breakthrough seizures in the following years. Over time, her seizure pattern evolved, with brief myoclonic seizures replacing the initial semiology. Adequate seizure control was not achieved despite trials of levetiracetam, topiramate, and clonazepam, both individually and in combination. However, with the introduction of a ketogenic diet, the patient demonstrated a fair response. No recent EEG was available for assessment.

Her developmental history is marked by severe global developmental delay, affecting motor, language, and cognitive domains. She does not fix or follow visual stimuli and is nonverbal.

The parents are second-degree cousins. The mother had one previous miscarriage. There is no known family history of a similar condition.

On physical examination, the patient appeared microcephalic with subtle dysmorphic features. Head circumference was 45.5 cm (below the 3rd percentile), height: 121.5 cm (60th percentile) and weight was 22.2 kg (60th percentile).

Additional dysmorphic features included rounded eyebrows with mild synophrys, wide, prominent eyes, mild distichiasis with long upper eyelashes, macro-otia with a prominent ear tragus, greek nose, prominent vermilion of the lips with a deep cupid's bow and pronounced commissures, a central pad on the upper lip, mild jowl.

She also exhibited contractures in both hands with tapering fingers, a swan-neck deformity of the right hand, and a flexion deformity of the distal joint of the second right finger. Severe contractures of both ankles were also noted.

Neurological examination showed axial hypotonia with appendicular hypertonia, diffusely increased deep tendon reflexes, without clonus, bilateral extensor plantar responses. The remainder of the examination was unremarkable.

Extensive laboratory and genetic testing yielded normal results, including complete blood count, thyroid function tests, renal profile, plasma glucose, and bone profile, tandem mass spectroscopy, urine organic acids, liver function tests, ammonia, lactate, and quantitative plasma amino acids, very long-chain fatty acids, hexosaminidase A & B levels, and normal carbohydrate-deficient transferrin, karyotype.

VEP confirmed the absence of P100 bilaterally. Brain MRI showed callosal agenesis and polymicrogyria.

Family F-V

The proband was a naturally conceived male fetus, the fifth pregnancy of a consanguineous first-cousin Pakistani couple. An ultrasound scan at 17 weeks of gestation showed abnormal limb posture, with flexed arms and hands and hyperextended legs with bilateral talipes. Additionally, severe ventriculomegaly and a hypoplastic cerebellum were observed. The couple opted to discontinue the pregnancy.

The couple had previously experienced two similarly affected pregnancies. The first pregnancy involved a male fetus. A mid-trimester ultrasound scan at 22 weeks of gestation revealed an appropriately grown fetus with multiple abnormalities, including a suspected single cerebral ventricle and a small cerebellum. Dilation of the right renal pelvis, abnormal hand posture, and talipes were also noted. The pregnancy was discontinued at 24 weeks due to multiple fetal anomalies.

Postmortem examination showed that the fetus was in the 2nd percentile for growth, with an average head size. Dysmorphic features included prominent eyes, severe micrognathia, and low-set ears. The fingers exhibited overlapping with severe camptodactyly and bilateral single transverse palmar creases. The legs had flexion contractures at the groins and knees, with severe bilateral talipes. Additionally, a parietal fontanelle and a large Wormian bone were present. The brain was mildly enlarged, with an interhemispheric arachnoid cyst causing displacement of the hemispheres. The corpus callosum was thin, and aqueductal stenosis was noted. Histological analysis revealed numerous globular bodies in the white matter, suggestive of axonal bulbs.

The second affected fetus was female. Detailed US scans at 20 weeks of gestation detected postural abnormalities, a small cerebellum, and a subthalamic cyst in the brain. Fetal brain MRI confirmed the presence of a small cerebellum associated with an enlarged cisterna magna. Severe ventriculomegaly involving the lateral and the third ventricles with absent corpus callosum and septum pellucidum, and a large midline interhemispheric cyst. Minimal fetal movement, bilateral talipes, and abnormal hand positioning were also observed. Other organ systems appeared normal. The pregnancy was terminated at 21 weeks of gestation.

Postmortem examination revealed an appropriately grown fetus with mild nuchal oedema. There was mild fixed flexion of the elbows and abnormal positioning of the hands. The legs exhibited scissoring, with contractures at the groins. The knees were in fixed extension, and there were bilateral talipes with rocker-bottom feet. Facial features included downslanting palpebral fissures, micrognathia, and mildly low-set ears. Histological analysis of skeletal muscle showed generalized wasting with atrophy of the distal muscles. The diaphragm was thin, and bilateral lung grooving was observed due to muscle loss between the ribs. Axonal spheroids were identified in the peripheral nerves and intestinal neuronal plexuses.

A Wormian bone was noted at the occiput. Examination of the brain revealed an enlarged cisterna magna, a hypoplastic cerebellum, a small brainstem and spinal cord, dilated lateral ventricles, abnormal temporal lobes, an absent corpus callosum, and an interhemispheric arachnoid cyst. Widespread, abundant axonal spheroids were found in the brainstem, cerebral hemispheres, and nerve roots, along with absent olivary nuclei. The pyramidal tracts appeared underdeveloped.

Genetic investigations in the affected fetuses included normal quantitative fluorescent-polymerase chain reaction (QF-PCR) for trisomies 13, 18, 21 with no evidence of sex chromosome aneuploidy, normal microarrays and normal *PLA2G6* sequencing and multiplex ligation-dependent probe amplification (MLPA). WES did not detect any definite pathogenic variants however a biparentally inherited frameshift variant NM_058169.4: c.316del; p.A106Pfs*20 was detected in the *BORCS5* gene. This was present in all three affected fetuses.

Regarding family history, the couple has two healthy sons. The father has had profound hearing loss since birth and has suffered from epilepsy since early adulthood. He also has chronic renal disease and borderline polycythemia of unknown cause. The maternal grandmother experienced recurrent miscarriages, and a paternal aunt had three early miscarriages.

Family F-VI

A 37-year-old female, Gravida 6, Para 0, was referred for prenatal genetic consultation at 28 weeks' gestation during her sixth pregnancy following the detection of hydrocephalus, cerebellar hypoplasia, and persistent hand fisting throughout the examination. The father, aged 41, and the mother were first-degree paternal cousins of Egyptian descent. Both parents were completely healthy, with normal chromosomal analyses and an unremarkable family history.

The patient's obstetric history was significant for four early neonatal deaths occurring within the first two days of life. During her fifth pregnancy, a retrospective evaluation of the second-trimester ultrasound revealed hydrocephalus, cerebellar hypoplasia, and abnormal positioning of the hands and feet. Chromosomal analysis performed for the last neonate revealed a normal male karyotype, though no further genetic testing was conducted. The mother also reported markedly reduced fetal movements in all her previous pregnancies as well as in the current one.

Ultrasound examination was performed using a Voluson E8 system, which confirmed polyhydramnios with poor visualization of the stomach. A three-dimensional anomaly scan identified a male fetus with biometric parameters within normal limits. The diagnosis of arthrogryposis multiplex congenita (AMC) was suspected based on significantly reduced fetal movements, fixed extended knees with bilateral talipes equinovarus, and minimal arm movements with clenched hands. The absence of pterygia further supported this diagnosis. A fetal neurosonogram revealed severe ventriculomegaly, characterized by grossly dilated lateral ventricles with dangling choroid plexuses, along with agenesis of the corpus callosum. Cerebellar hypoplasia was also noted, with a transverse cerebellar diameter measuring approximately at the fifth percentile for the corresponding gestational age.

Further examination of the fetal face showed dysmorphic features, including midface hypoplasia, hypertelorism, a depressed nasal bridge, down-slanting palpebral fissures, micrognathia, and low-set ears. Bilateral hydronephrosis was observed, with the renal pelvis measuring 8 mm bilaterally. Given the complexity of findings and the lack of a definitive clinical diagnosis, amniocentesis was performed for whole-exome sequencing to determine the underlying genetic cause, assess recurrence risk, and explore reproductive options for future pregnancies.

The couple was counseled regarding the high probability of an unfavorable prognosis due to the combination of severe hydrocephalus and AMC. Given the anticipated poor outcome, they opted for pregnancy termination at 25 weeks' gestation, and an autopsy was declined. The male fetus weighed 1,210 grams, corresponding to the 50th percentile. Postmortem examination confirmed the ultrasound findings, including characteristic dysmorphic facial features, such as hypertelorism, down-slanting palpebral fissures, midface hypoplasia, low-set ears, a depressed nasal bridge, and micrognathia. Arthrogryposis multiplex congenita was also confirmed, with multiple joint contractures, clenched hands, and bilateral club feet. No pterygia were observed.

WES in the fetus revealed the *BORCS5* homozygous nonsense variant NM_058169.4: c.417C>G; p.Y139*.

Family F-VII

The mother was a 24-year-old woman of Pakistani descent, and the father was of the same age and descent. The couple was healthy and consanguineous, being first cousins. Their family history was non-contributory, with no known cases of intellectual disability, congenital abnormalities, inherited conditions, or recurrent miscarriages.

Their first pregnancy was conceived naturally. An ultrasound performed at 13.5 weeks of gestation showed a nuchal translucency of 1.5 mm. Enhanced first-trimester screening (eFTS) indicated a low risk for trisomy 21 and 18. However, a detailed fetal ultrasound at 18 weeks and 5 days revealed ventriculomegaly, cerebellar hypoplasia, and severe ventriculomegaly, with the lateral ventricles measuring over 15 mm. Additional findings included micrognathia, overlapping fingers on both hands (fixed flexed fingers on the right and fixed extended fingers on the left), and crossed legs throughout the ultrasound examination.

Following genetic counselling, the couple opted for amniocentesis, which revealed a normal male chromosomal microarray analysis (CMA). Given the severity of the findings, the couple decided to terminate the pregnancy, permitting an external autopsy. The autopsy findings included a male foetus at 22 weeks of gestation with an enlarged, rounded head, mild hypertelorism, a high-arched palate with a posteriorly positioned tongue, and mild micrognathia. Additional anomalies included bilateral axillary pterygia, flexed hip positions, genu recurvatum of both knees, a clenched right hand with overlapping digits, irregular positioning of the left-hand fingers with simplified palmar creases, bilateral rocker-bottom feet, and overlapping toes on the right foot. Growth parameters were appropriate for gestational age.

A few months later, the couple conceived again. Early ultrasound confirmed a single intrauterine pregnancy. At 12.1 weeks of gestation, the nuchal translucency measured 1.6 mm. A detailed foetal ultrasound and neurosonogram at 20 weeks and 5 days revealed cerebellar and vermian hypoplasia, severe ventriculomegaly with both lateral ventricles measuring over 15 mm, and "dangling" choroids. The brainstem appeared thin and narrow, and micrognathia was noted. There was reduced muscle bulk and decreased fetal movements. Multiple joint contractures were present,

with fixed hand and elbow positions, hyperextended wrists, and both hips and knees held in a hyperflexed position.

Subsequent fetal MRI confirmed a single intrauterine gestation with transverse presentation. Notable findings included a diffusely dysmorphic brainstem with severe aqueduct stenosis and a prominent tectum. Marked proximal hydrocephalus involved the lateral and third ventricles. The cerebellar hemispheres and vermis were severely hypoplastic, with suspected poor differentiation superiorly. The corpus callosum and septum pellucidum were absent, and a large midline interhemispheric cyst/ventricular diverticulum was present. Dehiscence of the mesial cortical mantle was noted, along with reduced parenchymal volumes bilaterally. The foetus exhibited markedly reduced motion, hyperextended extremities, and arthrogryposis. No acute diffusion abnormalities or hemorrhagic foci were observed. Additional findings included micrognathia, diffuse scalp oedema extending into the nuchal region, focal buckling of the right frontal bone, and generalized muscle bulk reduction.

Given the recurrence of findings similar to their first pregnancy, the couple was counselled and opted for pregnancy termination. CMA revealed a normal female karyotype. Autopsy findings included microcephaly with hypoplasia of major neuronal groups, including the brainstem tegmental nuclei, inferior olivary nuclear complex, basis pontis, dentate nucleus, thalamus, and basal ganglia. Widespread axonal dystrophy was noted in the brainstem and internal capsule, with loss of axonal tracts in the corticofugal pathways, cerebellar peduncles, and corpus callosum. Additional abnormalities included posterior cerebral cortex maturation anomalies, aqueduct atresia, ventriculomegaly, hippocampal dysplasia, periventricular nodular heterotopia, and basal leptomeningeal heterotopia. The principal pathological findings indicated a neuroaxonal dystrophy with neural migration and maturation anomalies, as well as long tract hypoplasia.

Trio WES was performed for both affected fetuses and showed a homozygous frameshift indel in *BORCS5* NM_058169.4: c.316del; p.(A106Pfs*20), which was shared by both fetuses and was heterozygous in both parents, as the only candidate gene variant.

Family F-VIII

The proband (F-VIII:2) is a 3-year-old girl, born at term via spontaneous vaginal delivery with a normal APGAR score and a birth weight of 2.7 kg. She was discharged home after birth.

Shortly after birth she was found to have low hemoglobin and platelets. She was admitted for a bone marrow aspirate, which demonstrated a leukoerythroblastic picture suggestive of osteopetrosis. A subsequent skeletal survey revealed generalized increased bone density, confirming the diagnosis.

She failed newborn hearing screening on multiple occasions, and brainstem evoked auditory response (BEAR) testing revealed bilateral mild-to-moderate sensorineural hearing loss. Global developmental delay was evident early, with partial head support at three months but no further milestone progression. Currently she can hold her head and roll to the sides, but she is unable to sit or speak. She does not fix nor follow with her eyes. Ophthalmologic assessment showed poor vision with bilateral ON atrophy, and visual evoked potentials demonstrated no response.

At 16 months, she developed progressive spasticity in the lower limbs, followed at 20 months by seizures characterized by arm and head extension with staring episodes, typically occurring after awakening in clusters with up to 10 episodes with short inter-episode breaks. EEG showed diffuse slowing with frequent generalized spike–wave discharges and runs of generalized fast beta activity, consistent with infantile spasms. She was started on Vigabatrin that led to partial improvement, but spasticity progressed despite Baclofen.

Brain MRI demonstrated dysplastic short CC, marked reduction of white matter bulk with markedly delayed myelination, small T2-hypointense thalami, hypoplasia of the pons with thinning of the midbrain, and small ON. She developed recurrent vomiting with gastroesophageal reflux, necessitating nasogastric feeding and planned gastrostomy.

At her last assessment at age of three years, she had microcephaly (head circumference 44 cm, –2.88 SD), failure to thrive (weight 8.7 kg, –3.84 SD; length 79.5 cm, –3.8 SD), persistent seizures (2–5 sudden flexion episodes/day), partial head control, inability to grasp objects, generalized spasticity (more pronounced in lower limbs), and exclusive nasogastric feeding without choking. She remains on Vigabatrin, Baclofen, and Esomeprazole.

The parents are first cousins. She has two healthy older siblings and one older sister with a similar presentation of infantile osteopetrosis with hearing and visual impairment in addition to global developmental delay and seizures in the form of infantile spasm. The sister underwent hematopoietic stem cell transplantation (HSCT) at 6 months, which improved bone density

radiographically but not the neurological manifestations. She had global developmental delay, progressive spasticity with leg scissoring, and seizures characterized by eye staring, back arching, and repetitive upper limb flexion, often upon awakening, accompanied by frothing of saliva. EEG showed epileptic encephalopathy with burst suppression, generalized polyspike discharges, and asynchrony. She partially responded to Vigabatrin, with Levetiracetam and Pyridoxine added later. MRI demonstrated partial agenesis of the CC and hypomyelination. BEAR testing revealed moderate sensorineural hearing loss, and ophthalmologic evaluation confirmed bilateral ON atrophy. Her condition progressed with severe neurological impairment, and she died at the age of 8 years from seizures and aspiration.

WES was performed in the proband only (F-VIII:2) and revealed the homozygous *BORCS5* variant.

Family F-IX

The proband was at 10 months of age when he was first evaluated at a local tertiary hospital for global developmental delay, failure to thrive, and irritability. He was the fourth child to be delivered to this consanguineous couple related as first cousins, originally from Oman. He was born at 40 weeks of gestation after clinically unremarkable pregnancy. Delivery was uncomplicated, and he had normal APGAR scores. All growth parameters were between the 10th percentile and 25th percentile for age. Although he was discharged within 48 hours of delivery, he was noted to be very irritable, and he had intermittently bloated abdomen with feeds. By the end of the first month of life, the parents noted that he was visually inattentive, and he was not reacting to light. By the time he was evaluated at the age of 10 months, only partial head support was noted. None of the expected developmental milestones for age were otherwise attained then. He was not fixing, following or tracking. He also showed evidence of failure to thrive with all growth parameters then dropping to just below the 3rd percentile for age. He was not encephalopathic and he had no dysmorphic features when examined then. He was very irritable, excessively crying during the exam. He had axial hypotonia with appendicular hypertonia and pyramidal signs.

Brain MRI done externally was reported to show prominence of the extra-axial CSF spaces with ventriculomegaly consistent with brain atrophy (image not available). EEG was reported to show bilateral posterior cerebral epileptiform activity. He had anemia, his aspartate aminotransferase and alkaline phosphatase were significantly elevated.

The parents were lost to follow up. When contacted in the context of update related to this study, the parents reported that the child had slowly progressed over the years into spastic quadriparesis with multiple joint contractures and had been in a chronic vegetative state with no appreciable development beyond moaning and head support. He died at the age of 12 years with cardiorespiratory failure.

The child had an older sister who presented similarly in early infancy. She was reported to have lost her ability to fix, follow or track at the age of 3 months although no clinical or parental concerns were raised about her earlier. She was also diagnosed with seizures and was started on anti-epileptics. By the time she was seen in clinic at the age of 10 years, she was bedbound in chronic vegetative state with no appreciable development except for head support and moaning. She had failure to thrive with weight at – 3 SD for age. Measurement of the length was hindered by the severe contractures. Head circumference was 50 cm at the 4th percentile for age. Reflexes could not be elicited and plantar responses were frozen.

Brain MRI performed at 5 months of age demonstrated dysplastic short CC, marked reduction of WM bulk with markedly delayed myelination, small T2-hypointense thalami, hypoplasia of the pons with thinning of the midbrain, and small ONs.

She continued to be in chronic vegetative state and died of cardiorespiratory failure at the age of 14 years.

Supplementary table 1. Details of identified BORCS5 variants

Family	Chromosome position (GRCh38)	cDNA (NM_058169)	Amino acid change	GnomAD MAF	Igenomix Database MAF	UCL Queen Square Genomics Database MAF	CADD	Polyphen-2	SIFT	Mutation Taster	SpliceAI
F-VIII, F-IX	12-12361350-G-A	c.202+1G>A	p.?	0	0	0	32	N/A	N/A	N/A	0.99 (Donor Loss)
F-IV	12-12435627-G-T	c.203-1G>T	p.?	0	0	0	35	N/A	N/A	N/A	1.00 (Acceptor Loss)
F-I, F-III	12-12435709-G-A	c.284G>A	p.R95Q	0.000003717 (6 het carriers)	0	0	29.5	probably_damaging	deleterious	disease_causing	0
F-II	12-12435721-A-C	c.296A>C	p.H99P	0	0	0	26.4	probably_damaging	deleterious	disease_causing	0
F-I	12-12465564-ACT-A	c.380_381delCT	p.L128Vfs*86	0.00001115 (18 het carriers)	0.00000792921 (1 HET carrier)	0	N/A	N/A	N/A	N/A	0
F-V, F-VII	12-12435740-GG-G	c.316delG	p.A106Pfs*20	0	0	0	N/A	N/A	N/A	N/A	0
F-VI	12-12465602-C-G	c.417C>G	p.Y139*	0.000004337 (7 het carriers)	0.0000158587 (2 HET carriers)	0	33	N/A	N/A	N/A	0

MAF=minor allele frequency; HET=heterozygous; N/A=not applicable

Supplementary figures

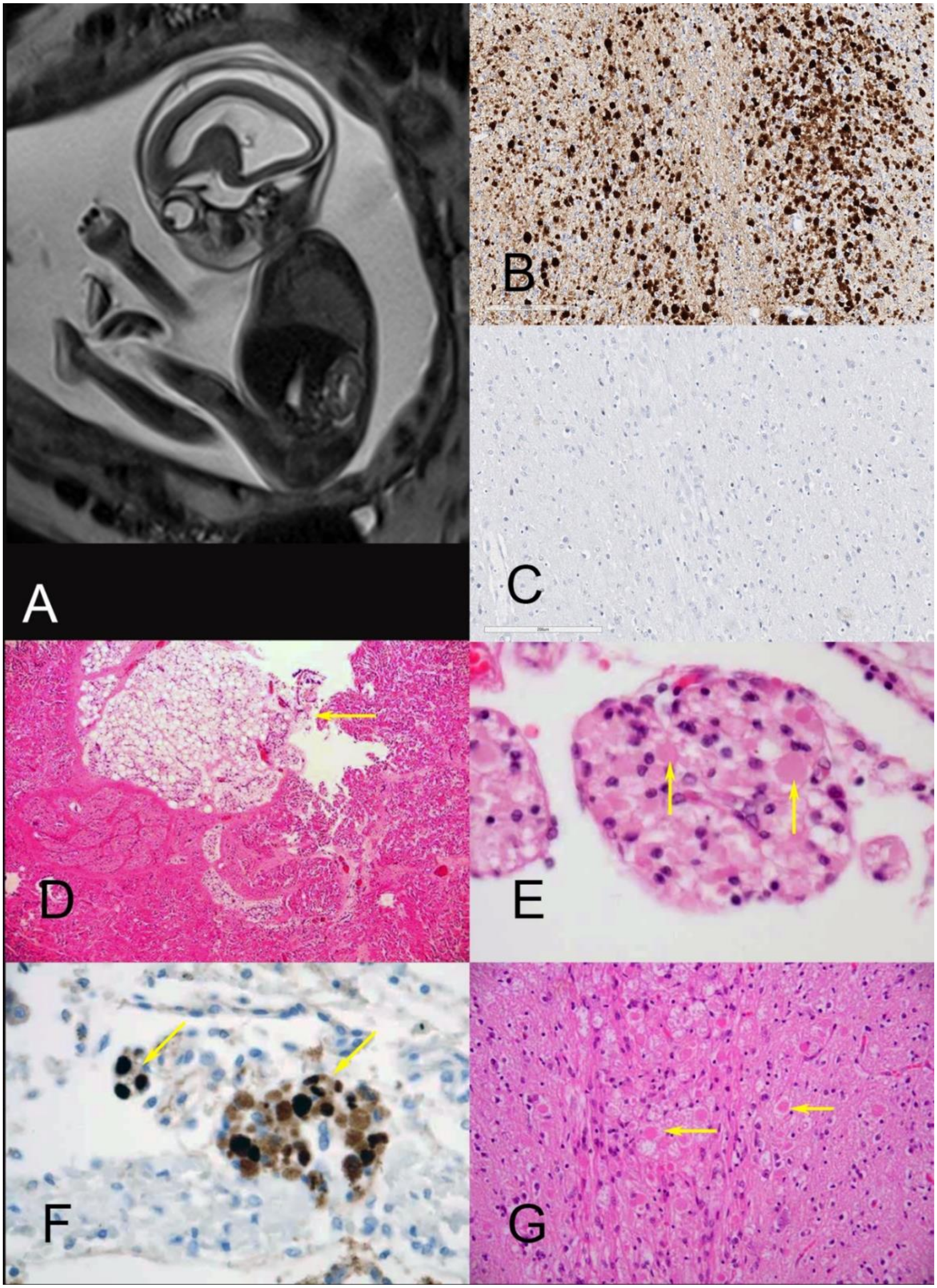

Supplementary Figure 1. A. MRI Sagittal T2-weighted image of the fetus F-VII:1 showing markedly reduced fetal motion with hyper extended extremities and generalized decreased muscle bulk consistent with arthrogryposis multiplex congenita (AMC). B. Internal capsule of the fetus F-VII:1 with innumerable axonal swellings staining for beta amyloid precursor protein. C. Negative staining for aggregated phosphorylated alpha synuclein. D. Skeletal muscle of case F-V:2. demonstrating small myofascicles with excess fibromyxoid perimysium and fat (arrow). E-F. Cranial nerve root of case F-V:2 containing many large eosinophilic axonal spheroids (arrow) (E), which stained positive for β-amyloid precursor protein (arrows) (F). G. Brainstem of case F-V:2 showing widespread eosinophilic axonal spheroids (arrows).

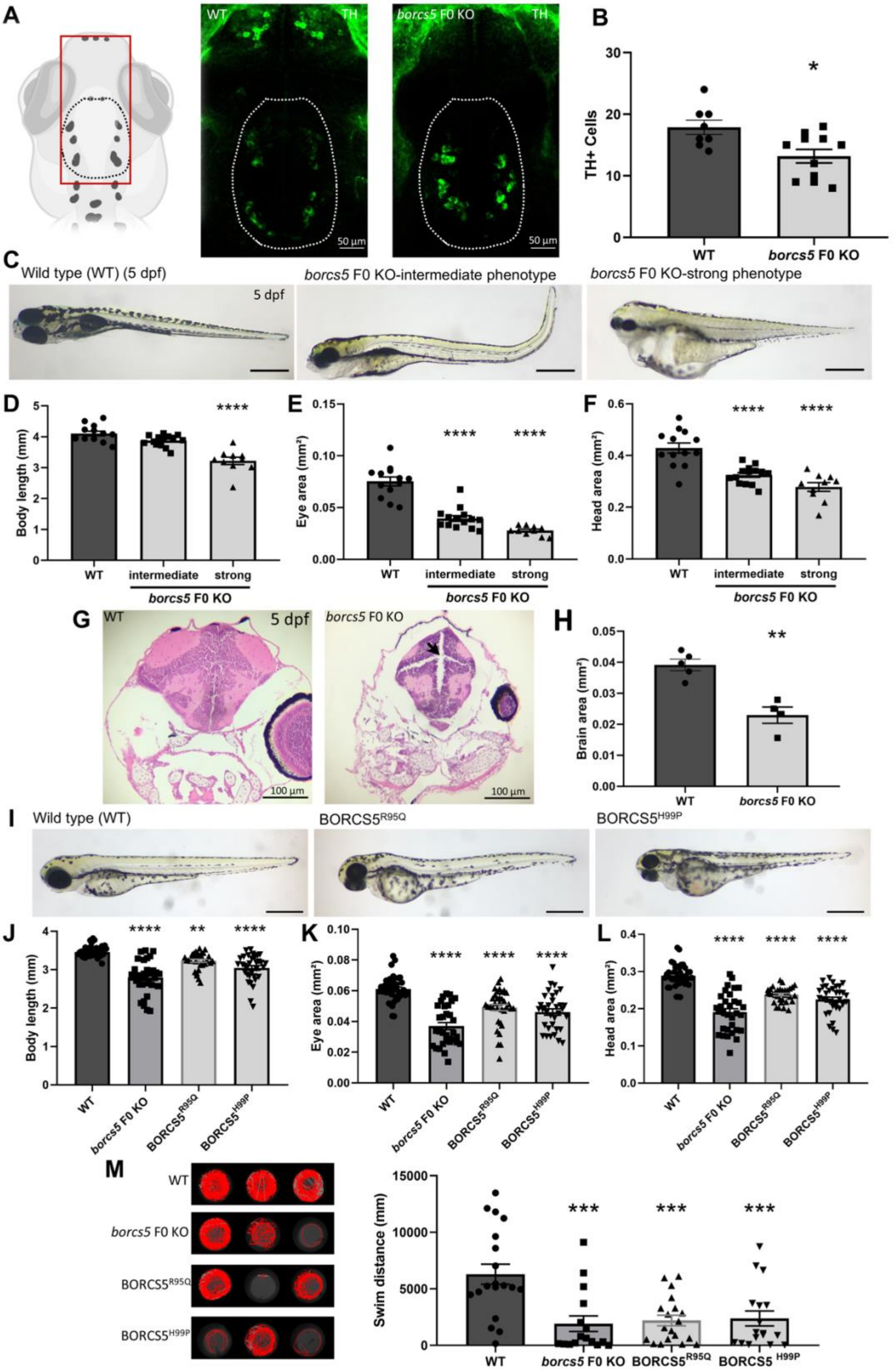

A
WT
TH
borcs5 F0 KO
TH
50 μm
50 μm
B
TH+ Cells
WT
borcs5 F0 KO
C
Wild type (WT) (5 dpf)
5 dpf
borcs5 F0 KO-intermediate phenotype
borcs5 F0 KO-strong phenotype
D
Body length (mm)
WT
intermediate
strong
borcs5 F0 KO
E
Eye area (mm²)
WT
intermediate
strong
borcs5 F0 KO
F
Head area (mm²)
WT
intermediate
strong
borcs5 F0 KO
G
WT
5 dpf
borcs5 F0 KO
100 μm
100 μm
H
Brain area (mm²)
WT
borcs5 F0 KO
I
Wild type (WT)
BORCS5R95Q
BORCS5H99P
J
Body length (mm)
WT
borcs5 F0 KO
BORCS5R95Q
BORCS5H99P
K
Eye area (mm²)
WT
borcs5 F0 KO
BORCS5R95Q
BORCS5H99P
L
Head area (mm²)
WT
borcs5 F0 KO
BORCS5R95Q
BORCS5H99P
M
WT
borcs5 F0 KO
BORCS5R95Q
BORCS5H99P
Swim distance (mm)
WT
borcs5 F0 KO
BORCS5R95Q
BORCS5 H99P

Supplementary figure 2. A. Tyrosine hydroxylase (TH) staining dopaminergic neurons at 3 dpf of WT and borcs5-ko larvae. Scale bars: 50 µm. B. Quantification of tyrosine hydroxylase (TH) positive neurons in diencephalic (paraventricular organ) region for WT, *borcs5* F0 KO (N=2, n=8-11). C. Morphology of zebrafish WT, and borcs5-ko larvae at 5 dpf. Scale bars: 500 µm. (D-F) Body length, eye size, and head size of WT, borcs5-ko larvae at 5 dpf (N=2, n=10-14). G. Midbrain sections stain with hematoxylin & eosin of larvae of 5 dpf. Scale bar: 100 µm. The arrow indicates ventriculomegaly in borcs5-ko larvae. H. Brain area comparison of borcs5-ko larvae (N=4) relative to WT (N=5). I. Morphology of zebrafish WT and variants models, $BORCS5^{R95Q}$ and $BOCRS5^{H99P}$ larvae at 3 dpf. Scale bars: 500 µm. (J-L) Body length, eye size, and head size of WT (N=3, n=40), borcs5-ko (N=3, n=32), $BORCS5^{R95Q}$ (N=2, n=32-35) and $BORCS5^{H99P}$ (N=2, n=34) larvae at 3 dpf (WT and borcs5 F0 KO data are the same as those presented in Figure 4 and are included here to facilitate comparison). (M) $BORCS5^{R95Q}$ (n=19) and $BORCS5^{H99P}$ (n=19) larvae show motor behavior comparable to borcs5-ko (n=19) and display impaired swim distance and velocity compared to WT (n=19). All data are represented as the mean ± SEM. Statistical significance was calculated by one-way ANOVA followed by Tukey's multiple comparisons tests (D-F, J-L,M), or Student's T-test (B and H). *P < 0.05; **P < 0.01; ****P < 0.0001.

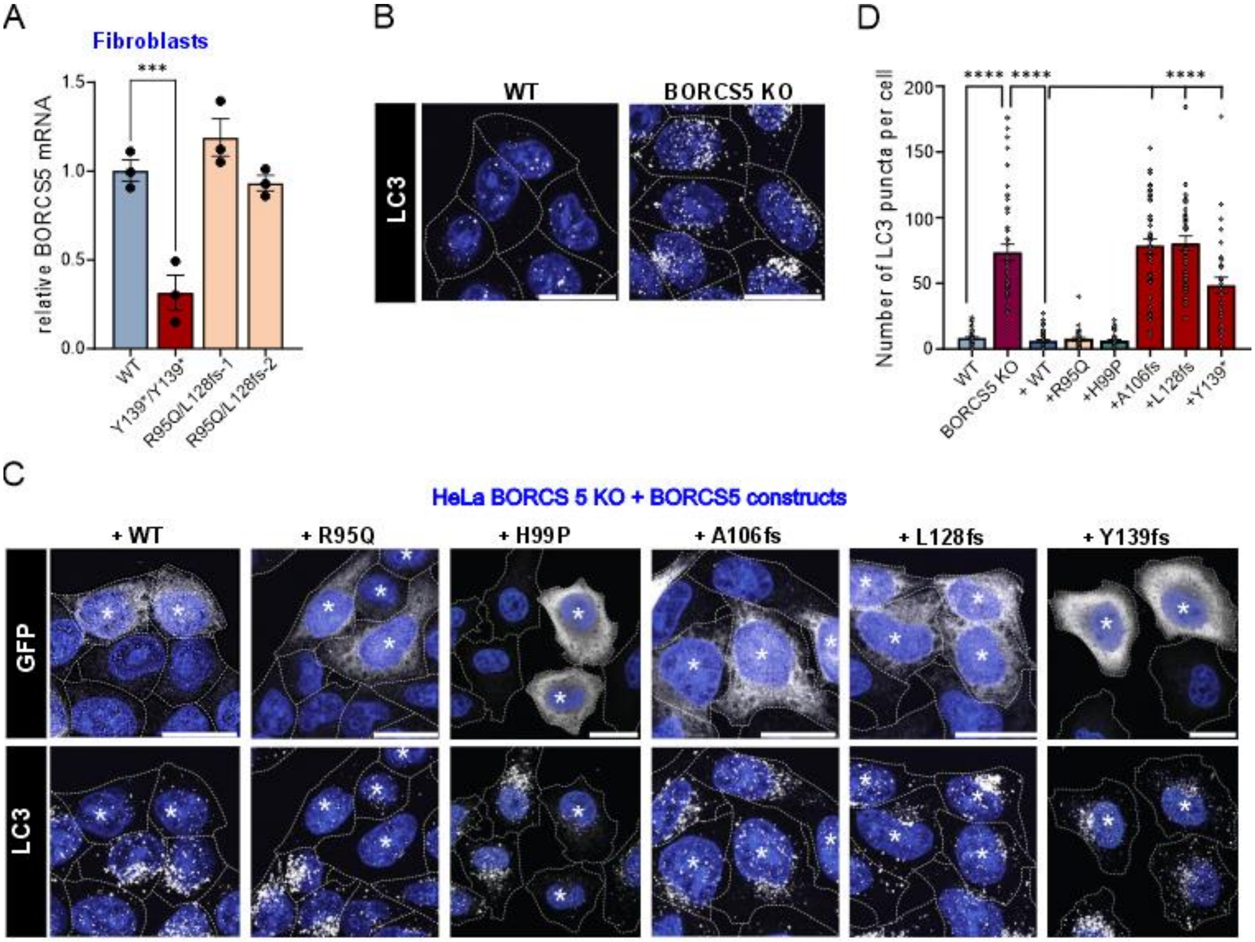

Supplementary figure 3: A. BORCS5 mRNA was analyzed by qPCR in fibroblasts and expressed as fold change over WT. Graph shows mean±SEM, N=3 independent samples. Statistics: One way ANOVA with Dunnette's post hoc, $F_{BORCS5\ mRNA}(3,8)=22.01$, P=0.0003, ***p=0.0008. B, C. ICC shows endogenous LC3 (white puncta) distribution in untransfected WT and BORCS5 KO HeLa cells as control. BORCS5 KO HeLa cells were transiently co-transfected with the indicated BORCS5 constructs and GFP. ICC shows endogenous LC3 distribution in GFP+ transfected cells (indicated by asterisk). Nuclei were labeled with DAPI (blue), and cell edges were outlined by fluorescent phalloidin (indicated by dashed lines). Scale bars: 20 µm. D. Quantification shows mean±SEM, N=3 independent experiments. Statistics: One-way ANOVA with Tukey's multiple comparisons test (mean of each column compared to the mean of every other column), $F_{LC3\ puncta}(7,320)=72.53$, $P<0.0001$. ****$p < 0.0001$.

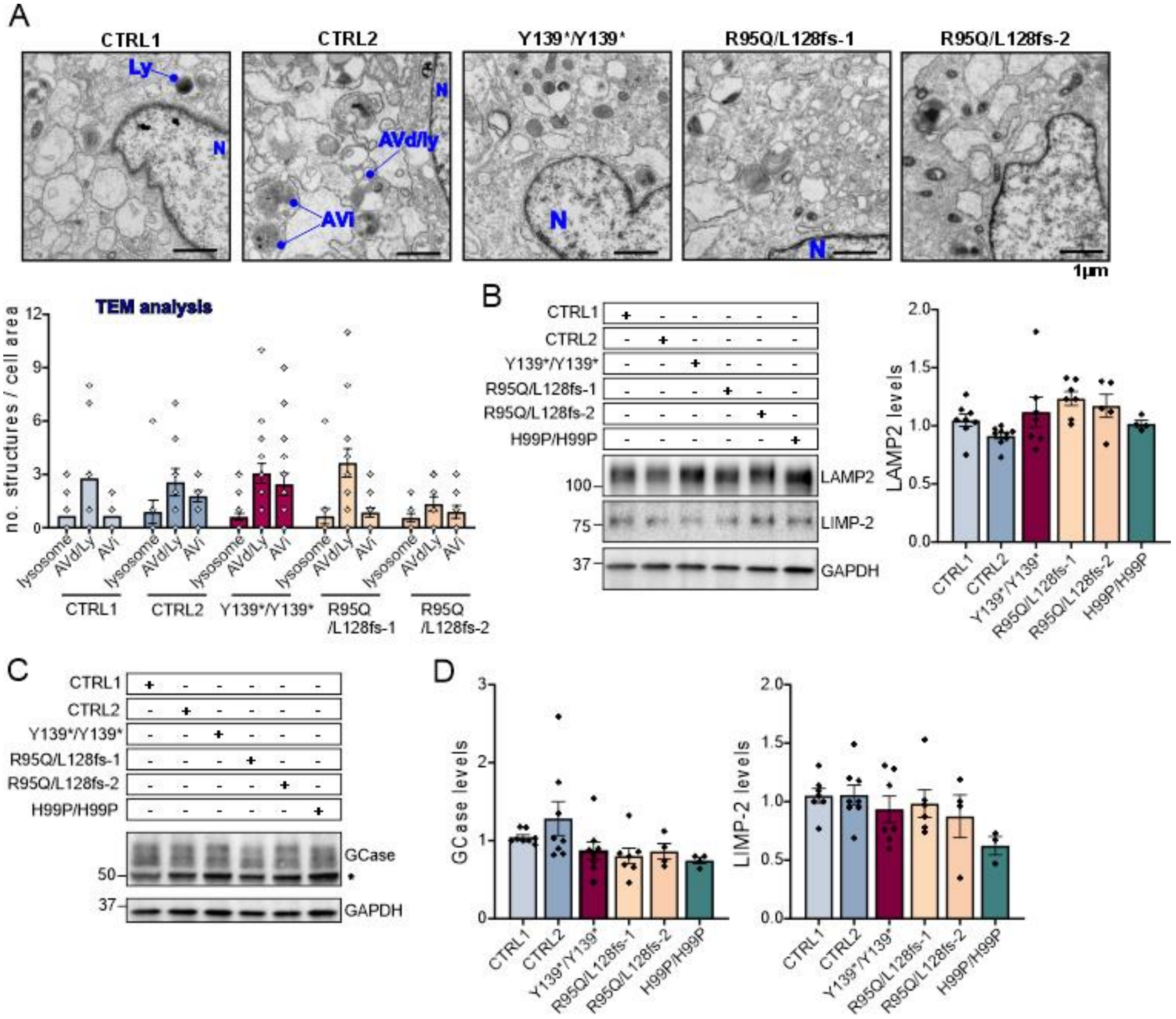

Supplementary figure 4: A. TEM of fibroblasts from the indicated *BORCS5* genotypes. Individual autophagic structures were classified according to previously published criteria.[58] Outlined insets are presented at higher magnification on the right, indicated by dashed lines. Abbreviations: N: Nucleus; Avi: Early/initial autophagic vacuole; Avd: Degradative autophagic vacuole/autolysosome; Ly: Lysosome. Graph shows mean±SEM of the number of individual structures identified in individual cells, N=9 to 18 individual cells per fibroblast line. Statistics: Two-way ANOVA $F_{interaction}(8,162)=1.371$, P=0.2131. B-D. WB and quantification of relative lysosomal protein amounts in the indicated control and BORCS5 patient fibroblasts. Graphs show mean±SEM over the mean of control lines, N=3-6 independent experiments. Statistics: One way ANOVA, $F_{LAMP2}(5,31)=2.86$, P=0.031; F_{LIMP-2} (5,26)=1.354, P=0.274, P=0.21; $F_{GCase}(5,30)=1.794$, P=0.144. Asterisk indicates non-specific band in the GCase blot.

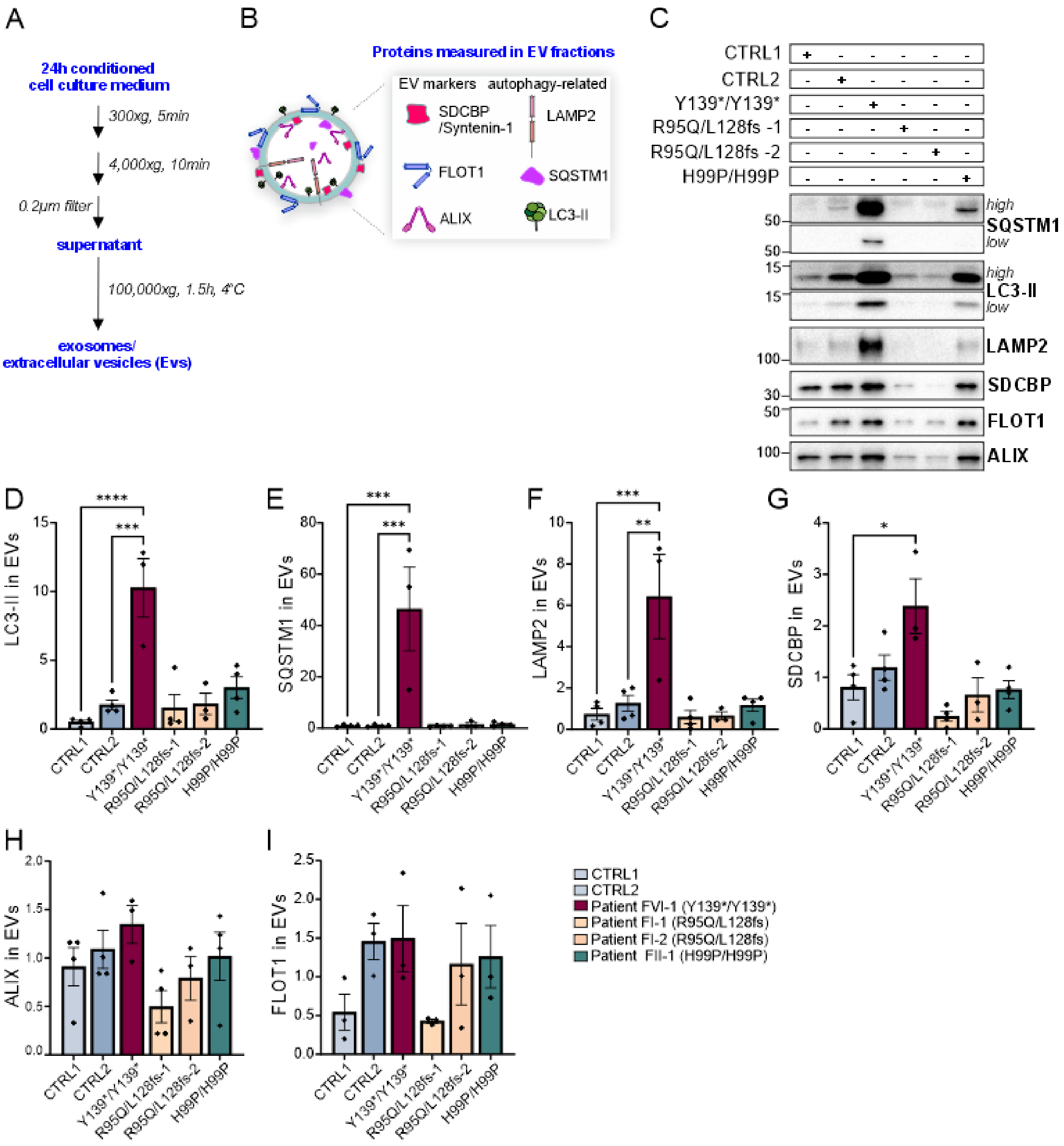

Supplementary figure 5: Schematic representation of fibroblast EV isolation via ultracentrifugation. B. Summary of proteins analyzed in exosome/extracellular vesicle (EV) fractions isolated from identical volumes of fibroblast conditioned medium via ultracentrifugation.

C. WB of EVs and quantification of autophagy/lysosome-related markers normalized to controls. D-I. Graphs show mean±SEM, N=3-4 independent experiments. Statistics: One way ANOVA with Tukey's multiple comparisons test (mean of each column compared to the mean of every other column). $F_{LC3\text{-}II}(5,16)=12.51$, $P<0.0001$. **** $p<0.0001$, *** $p=0.0002$; $F_{SQSTM1}(5,16)=10.65$, $P=0.0001$. ***$p=0.0002$; $F_{LAMP2}(5,16)=8.572$, $P=0.0004$. ***$p=0.0007$, **$p=0.0017$. $F_{SDCBP}(5,16)=6.691$, $P=0.0015$. *$p=0.011$; $F_{ALIX}(5,16)=1.843$, $P=0.1611$; $F_{FLOT1}(5,12)=1.743$, $P=0.1993$.

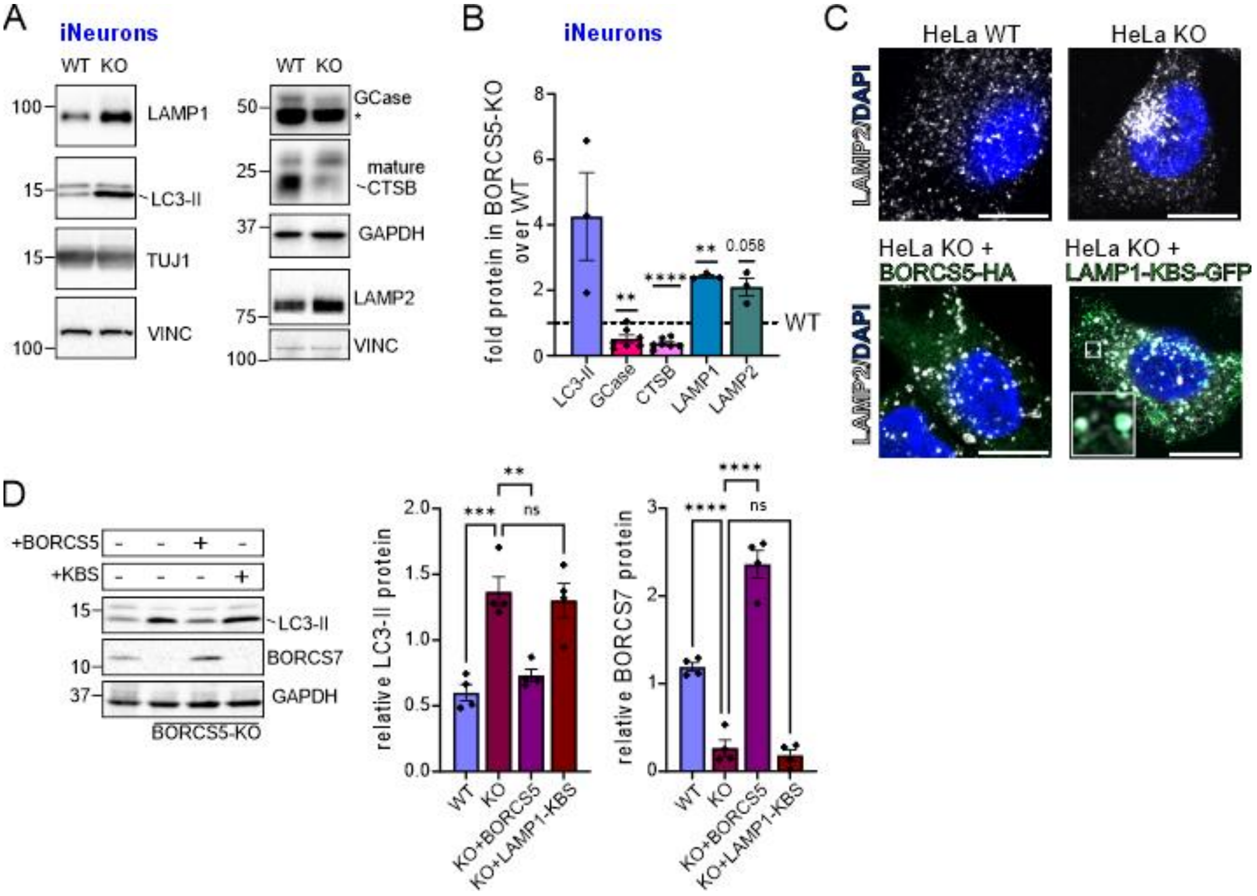

Supplementary figure 6: A. WB analysis of total lysates from iNeurons at day 21. Asterisk includes unspecific band in GCase blot. Markers GAPDH or VINC (Vinculin) were used as loading controls. B. Graphs show mean±SEM of the fold change in protein levels normalized to loading controls, N=3-7 independent experiments. Statistics: One sample t test, **** $P<0.0001$, ** $P<0.0012$. C. ICC shows LAMP2 particle (white) distribution in HeLa WT or BORCS5-KO cells with or without expression of LAMP1-KBS-GFP which promotes lysosomal anterograde trafficking. D. WB analysis of BORCS7 and LC3-II proteins, with GAPDH used as a loading control. Graphs show mean±SEM for fold change of protein levels, normalized to the mean of all conditions, N=4 independent experiments. Statistics: One way ANOVA with Dunnette's post hoc, compared to KO condition. $F_{LC3\text{-}II}(3,12)=16.79$, $P=0.0001$; $F_{BORCS7}(3,12)=103.7$, $P<0.0001$. **$p=0.0014$, ***$p=0.0003$, ****$p<0.0001$.

Supplemental references

1. Le Fevre A, Baptista J, Ellard S, Overton T, Oliver A, Gradhand E, et al. Compound heterozygous Pkd1l1 variants in a family with two fetuses affected by heterotaxy and complex Chd. *Eur J Med Genet.* 2020;63(2):103657.
2. Retterer K, Juusola J, Cho MT, Vitazka P, Millan F, Gibellini F, et al. Clinical application of whole-exome sequencing across clinical indications. *Genet Med.* 2016;18(7):696-704.
3. Mencacci NE, Kamsteeg EJ, Nakashima K, R'Bibo L, Lynch DS, Balint B, et al. De Novo Mutations in PDE10A Cause Childhood-Onset Chorea with Bilateral Striatal Lesions. *American journal of human genetics.* 2016;98(4):763-71.
4. do Couto NF, Queiroz-Oliveira T, Horta MF, Castro-Gomes T, and Andrade LO. Measuring Intracellular Vesicle Density and Dispersion Using Fluorescence Microscopy and ImageJ/FIJI. *Bio-protocol.* 2020;10(15):e3703.
5. Haeussler M, Schönig K, Eckert H, Eschstruth A, Mianné J, Renaud JB, et al. Evaluation of off-target and on-target scoring algorithms and integration into the guide RNA selection tool CRISPOR. *Genome Biol.* 2016;17(1):148.
6. Deen MC, Zhu Y, Gros C, Na N, Gilormini PA, Shen DL, et al. A versatile fluorescence-quenched substrate for quantitative measurement of glucocerebrosidase activity within live cells. *Proceedings of the National Academy of Sciences of the United States of America.* 2022;119(29):e2200553119.
7. Stirling DR, Swain-Bowden MJ, Lucas AM, Carpenter AE, Cimini BA, and Goodman A. CellProfiler 4: improvements in speed, utility and usability. *BMC Bioinformatics.* 2021;22(1):433.
8. Minakaki G, Safren N, Bustos BI, Lubbe SJ, Mencacci NE, and Krainc D. Commander complex regulates lysosomal function and is implicated in Parkinson's disease risk. *Science.* 2025;388(6743):204-11.
9. Abu-Remaileh M, Wyant GA, Kim C, Laqtom NN, Abbasi M, Chan SH, et al. Lysosomal metabolomics reveals V-ATPase- and mTOR-dependent regulation of amino acid efflux from lysosomes. *Science.* 2017;358(6364):807-13.
10. Kimmel CB, Ballard WW, Kimmel SR, Ullmann B, and Schilling TF. Stages of embryonic development of the zebrafish. *Dev Dyn.* 1995;203(3):253-310.
11. De Pace R, Maroofian R, Paimboeuf A, Zamani M, Zaki MS, Sadeghian S, et al. Biallelic BORCS8 variants cause an infantile-onset neurodegenerative disorder with altered lysosome dynamics. *Brain : a journal of neurology.* 2024;147(5):1751-67.
