# Supplementary material for: Pathogenic variants in BORCS5 cause a spectrum of neurodevelopmental and neurodegenerative disorders with lysosomal dysfunction
Source: J Clin Invest. 2026 Apr 21;136(11):e195336. doi: 10.1172/JCI195336 (PMC13221227; doi:10.1172/JCI195336)

Figure 5A

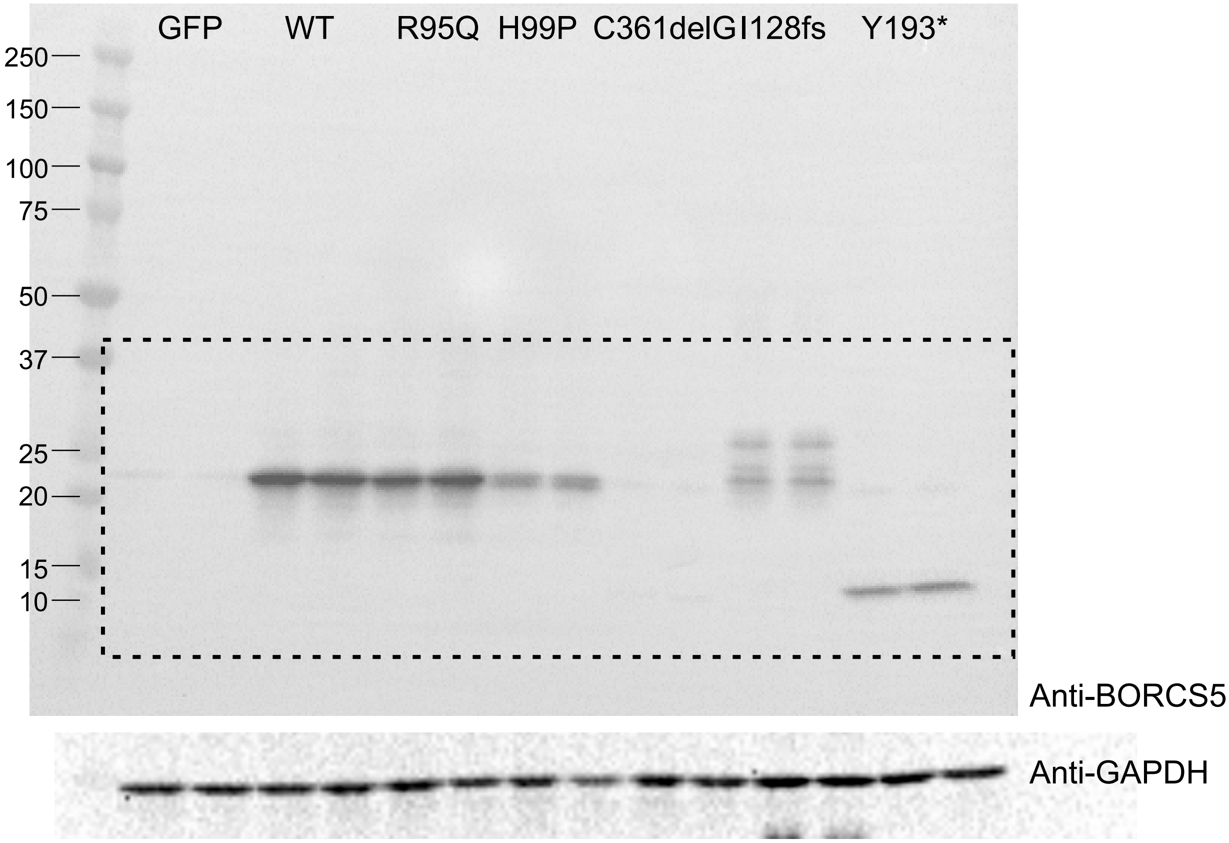

GFP
WT
R95Q
H99P
C361delG
I128fs
Y193*
250
150
100
75
50
37
25
20
15
10
Anti-BORCS5
Anti-GAPDH

Figure 5B

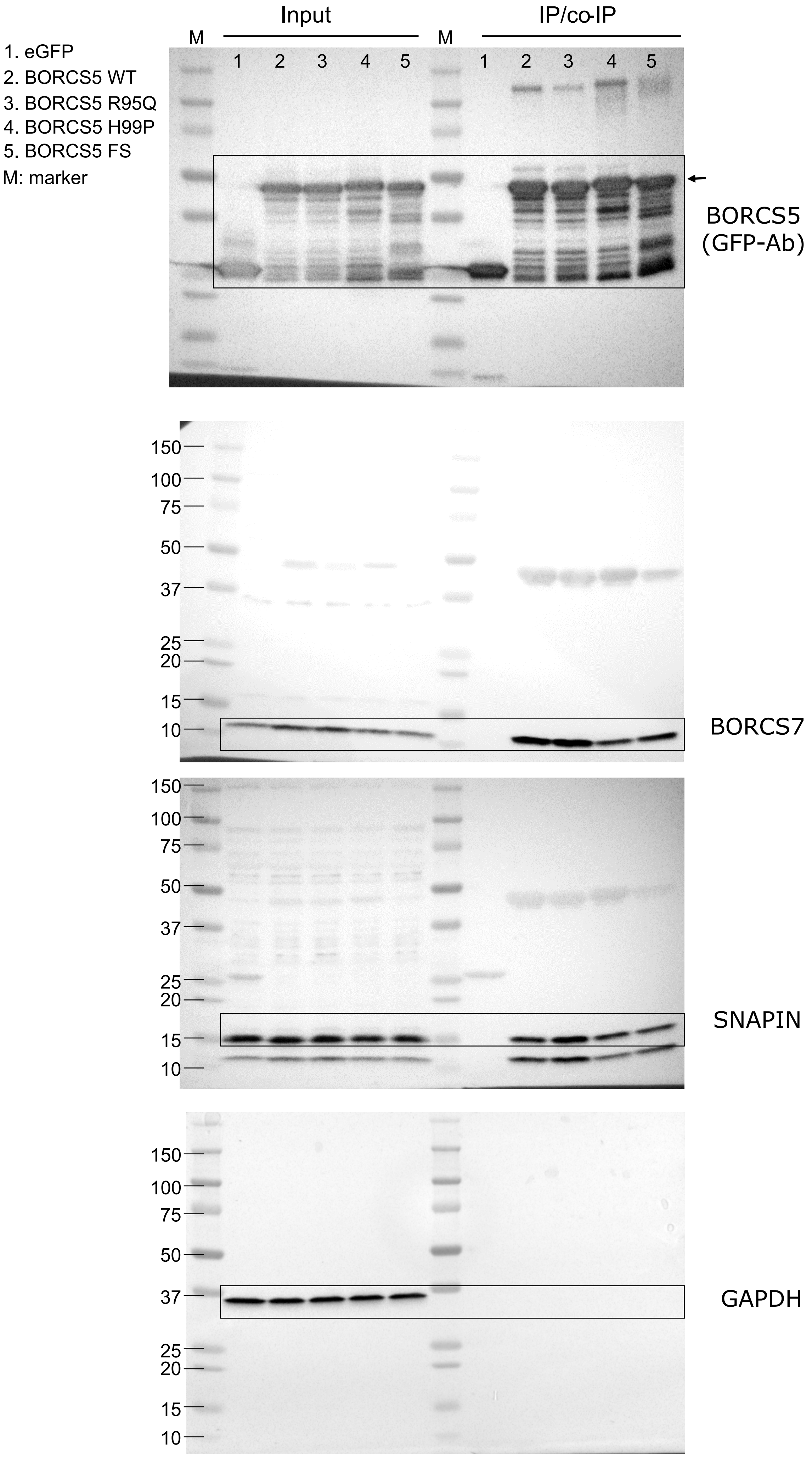
1. eGFP
2. BORCS5 WT
3. BORCS5 R95Q
4. BORCS5 H99P
5. BORCS5 FS
M: marker
Input
IP/co-IP
M
1
2
3
4
5
BORCS5
(GFP-Ab)
BORCS7
SNAPIN
GAPDH
150
100
75
50
37
25
20
15
10

Figure 6A

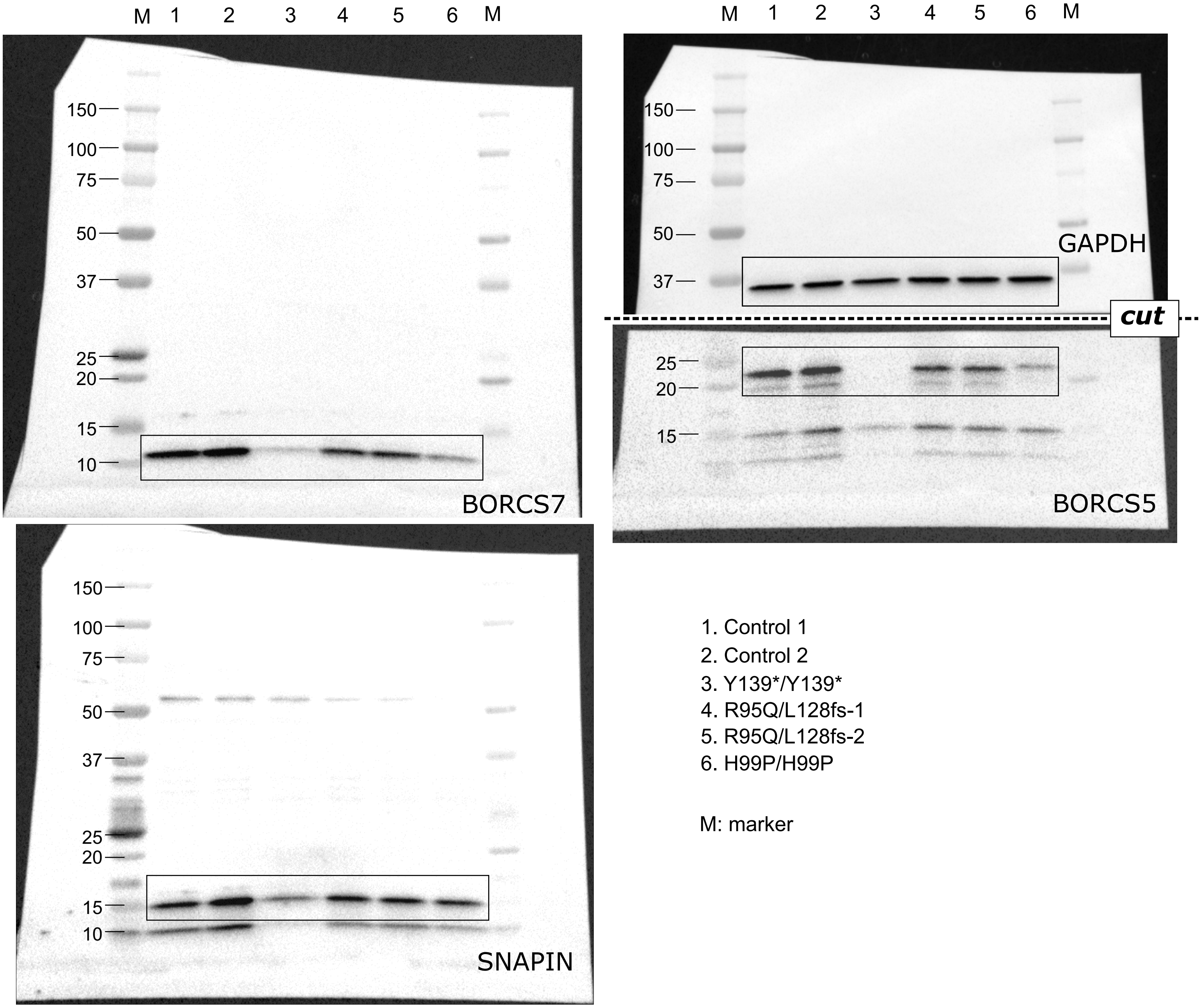

1. Control 1
2. Control 2
3. Y139*/Y139*
4. R95Q/L128fs-1
5. R95Q/L128fs-2
6. H99P/H99P

M: marker

Figure 8A

gel 1

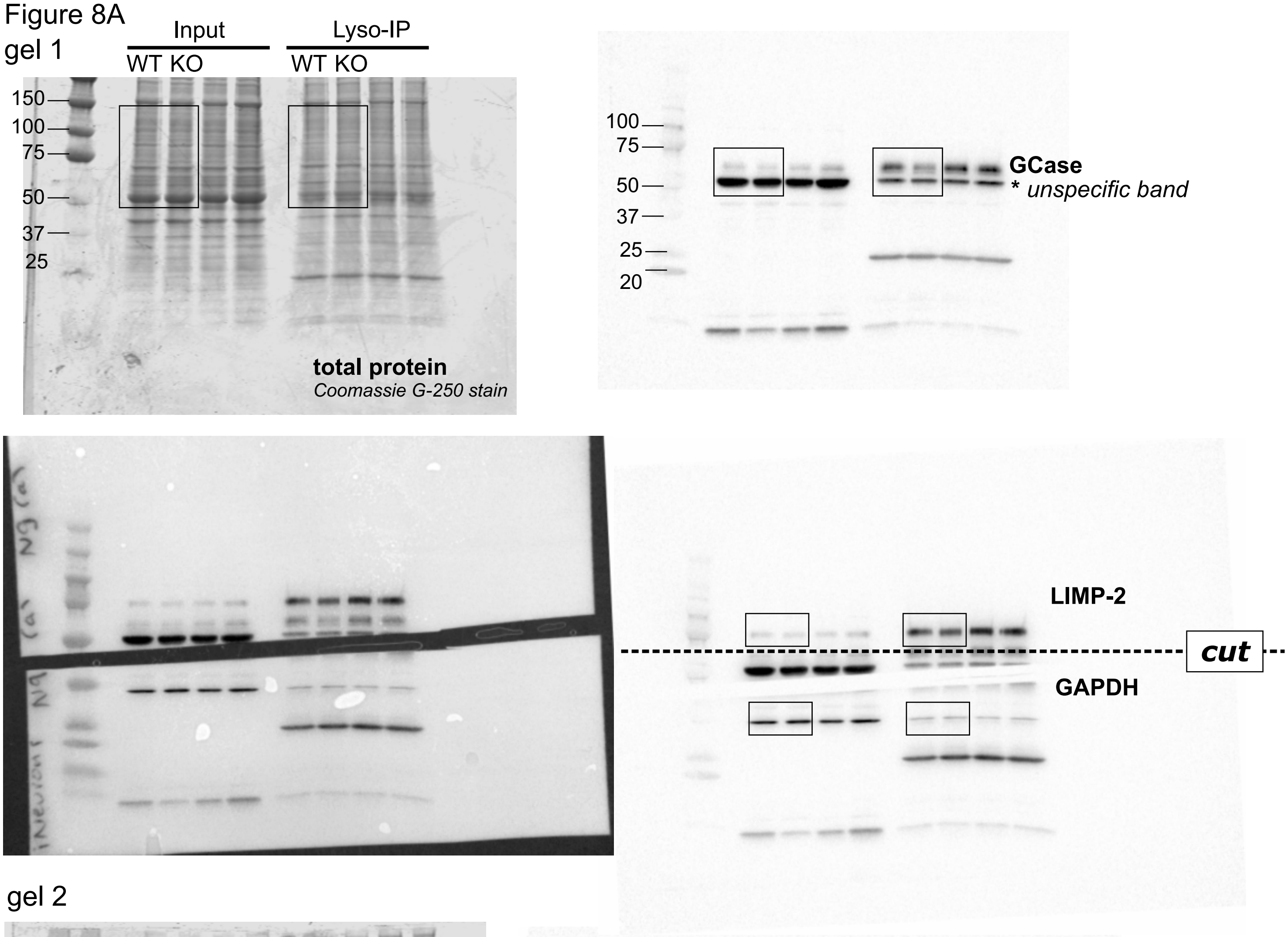

gel 2

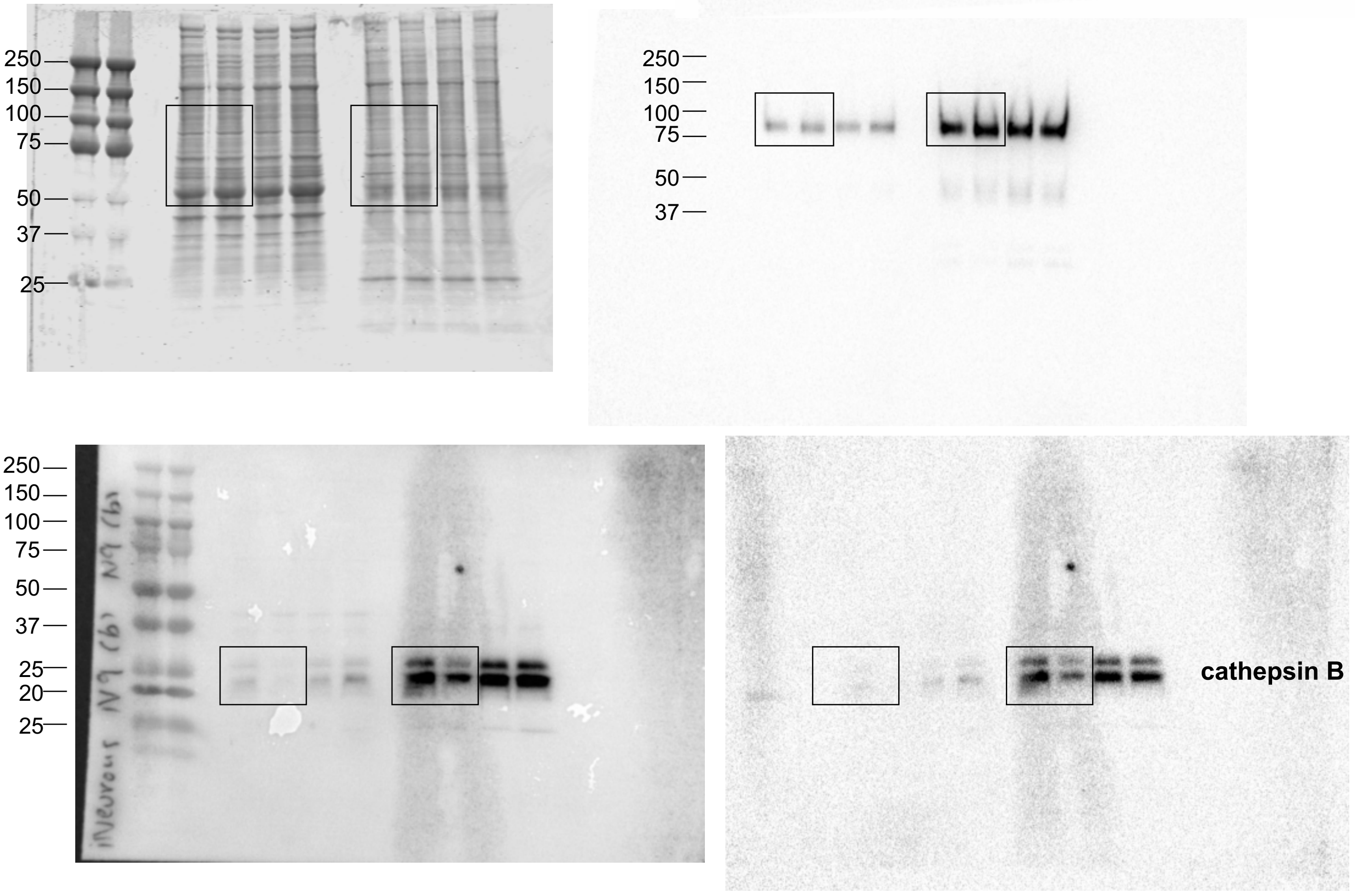

Supplemental Figure 4B

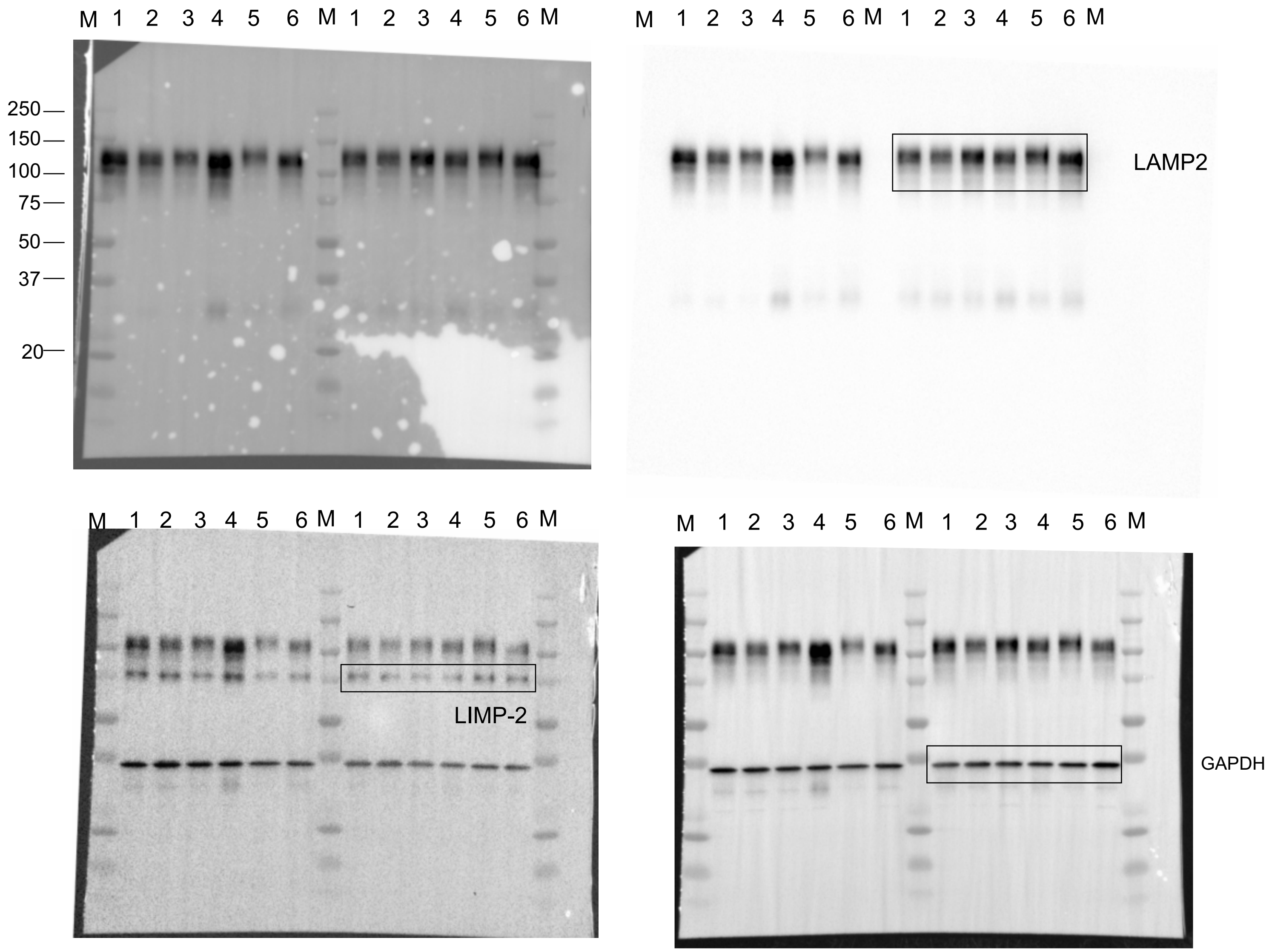

1. Control 1
2. Control 2
3. Y139*/Y139*
4. R95Q/L128fs-1
5. R95Q/L128fs-2
6. H99P/H99P

M: marker

Supplemental Figure 4C

1. Control 1
2. Control 2
3. Y139*/Y139*
4. R95Q/L128fs-1
5. R95Q/L128fs-2
6. H99P/H99P

M: marker

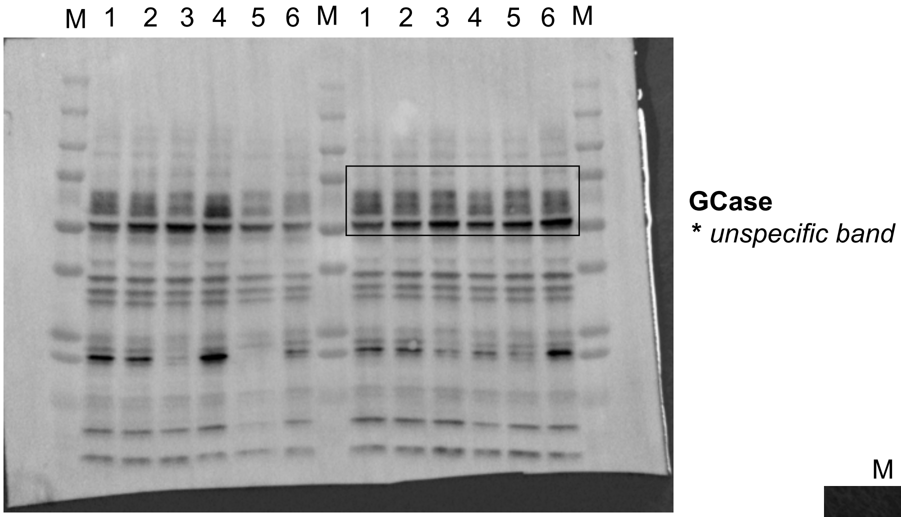

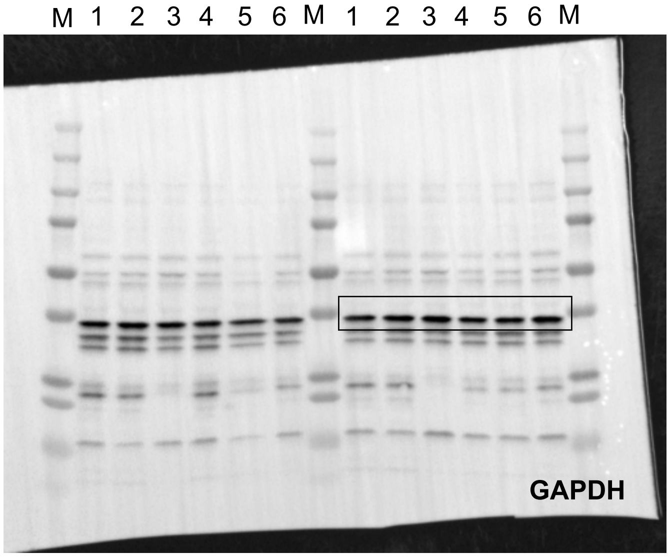

Supplemental Figure 5C

Lane 1: P100_N1_CTR1
Lane 2: P100_N1_CTR2
Lane 3: P100_N1_Patient FVI-1 (Y139*/Y139*)
Lane 4: P100_N1_Patient FI-1 (R95Q/L128fs)
Lane 5: P100_N1_Patient FI-2 (R95Q/L128fs)
Lane 6: P100_N1_Patient FII-1 (H99P/H99P)
Lane 7: P100_N2_CTR1
Lane 8: P100_N2_CTR2
Lane 9: P100_N2_Patient FVI-1 (Y139*/Y139*)
Lane 10: P100_N2_Patient FI-1 (R95Q/L128fs)
Lane 11: P100_N2_Patient FI-2 (R95Q/L128fs)
Lane 12: P100_N2_Patient FII-1 (H99P/H99P)

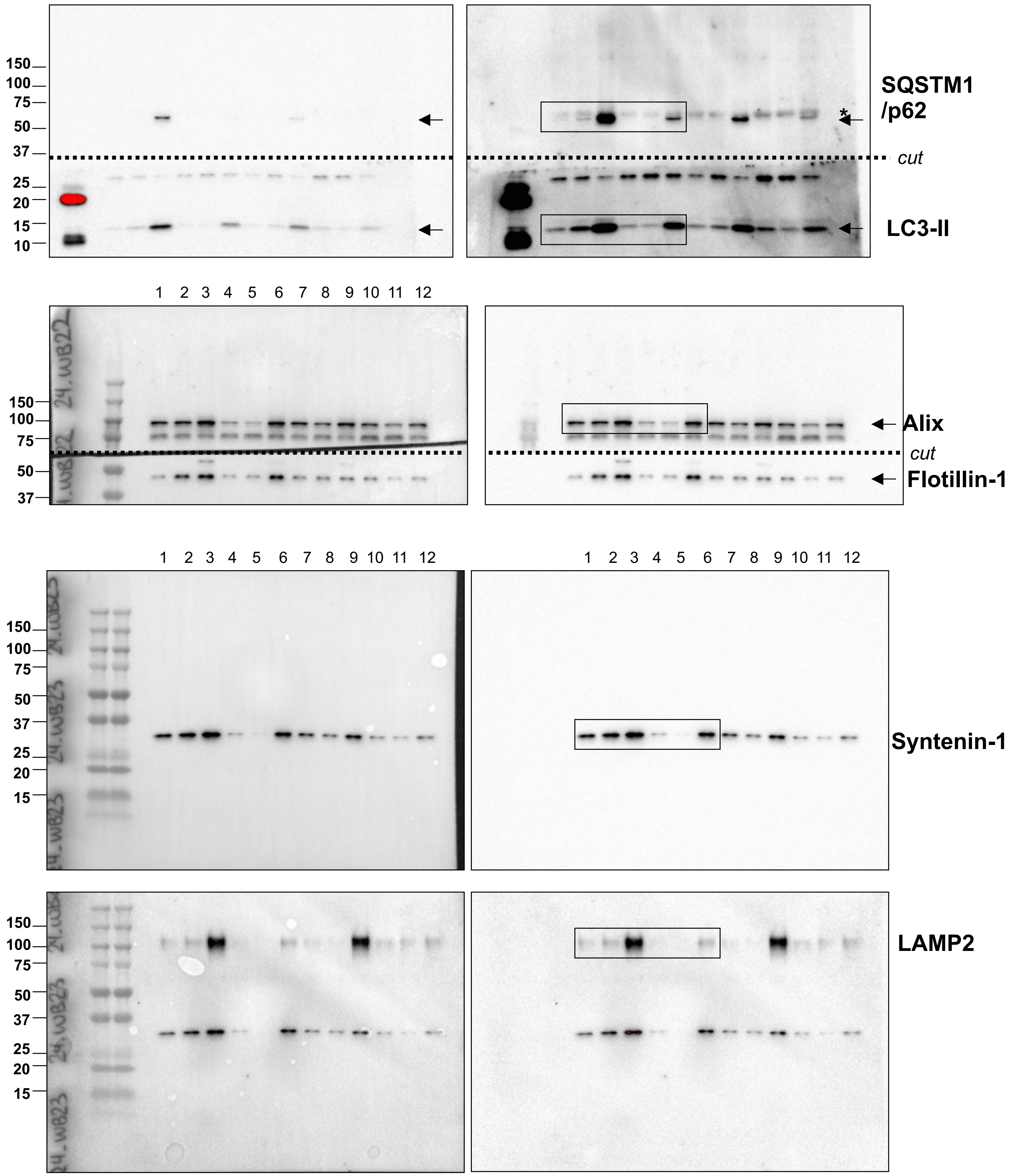

Supplemental Figure 6A

total lysates from WT or BORCS5 KO iNeurons

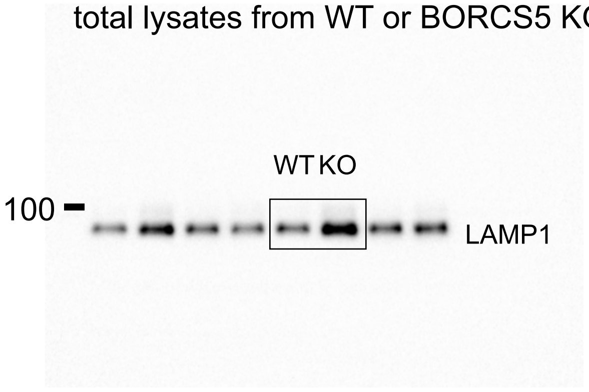

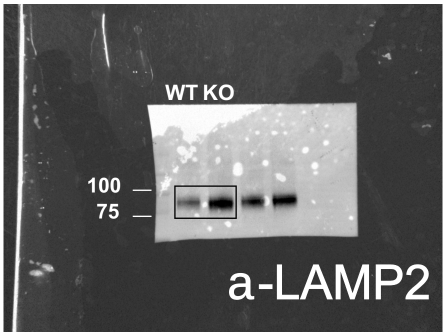

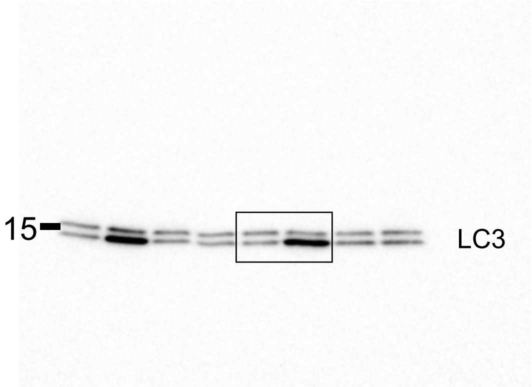

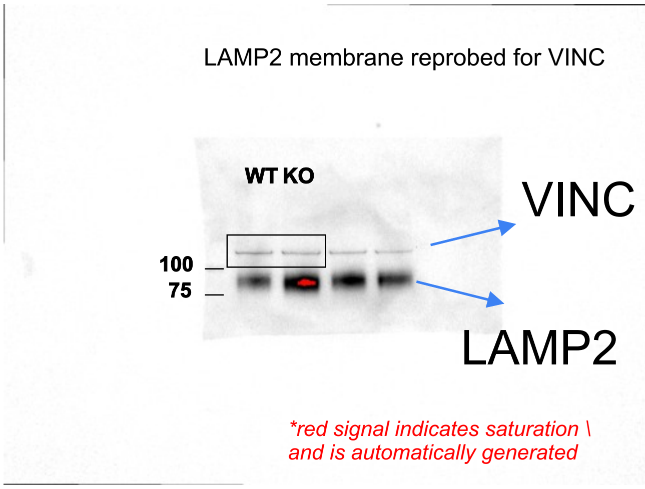

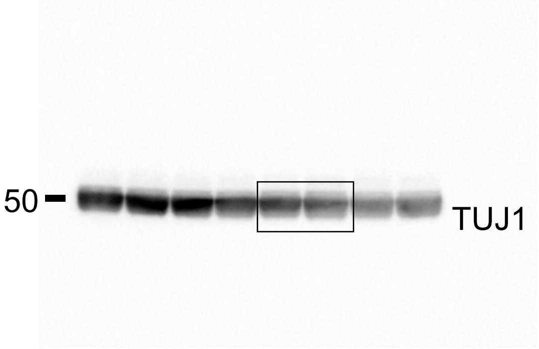

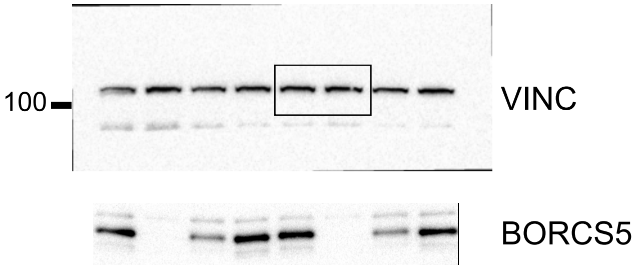

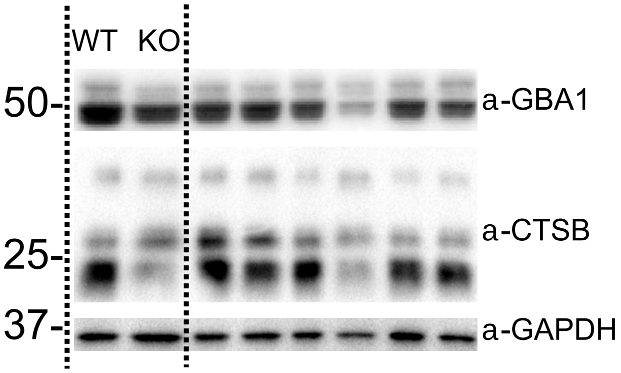

*Here we had to use cropped blots
due to limited material/availability for
probing at certain molecular weight

Supplemental Figure 6D

1. N1 HeLa WT
2. N1 HeLa BORCS5-KO
3. N1 HeLa BORCS5-KO + BORCS5-HA
4. N1 HeLa BORCS5-KO + LAMP1-KBS-GFP
5. N2 HeLa WT
6. N2 HeLa BORCS5-KO
7. N2 HeLa BORCS5-KO + BORCS5-HA
8. N2 HeLa BORCS5-KO + LAMP1-KBS-GFP

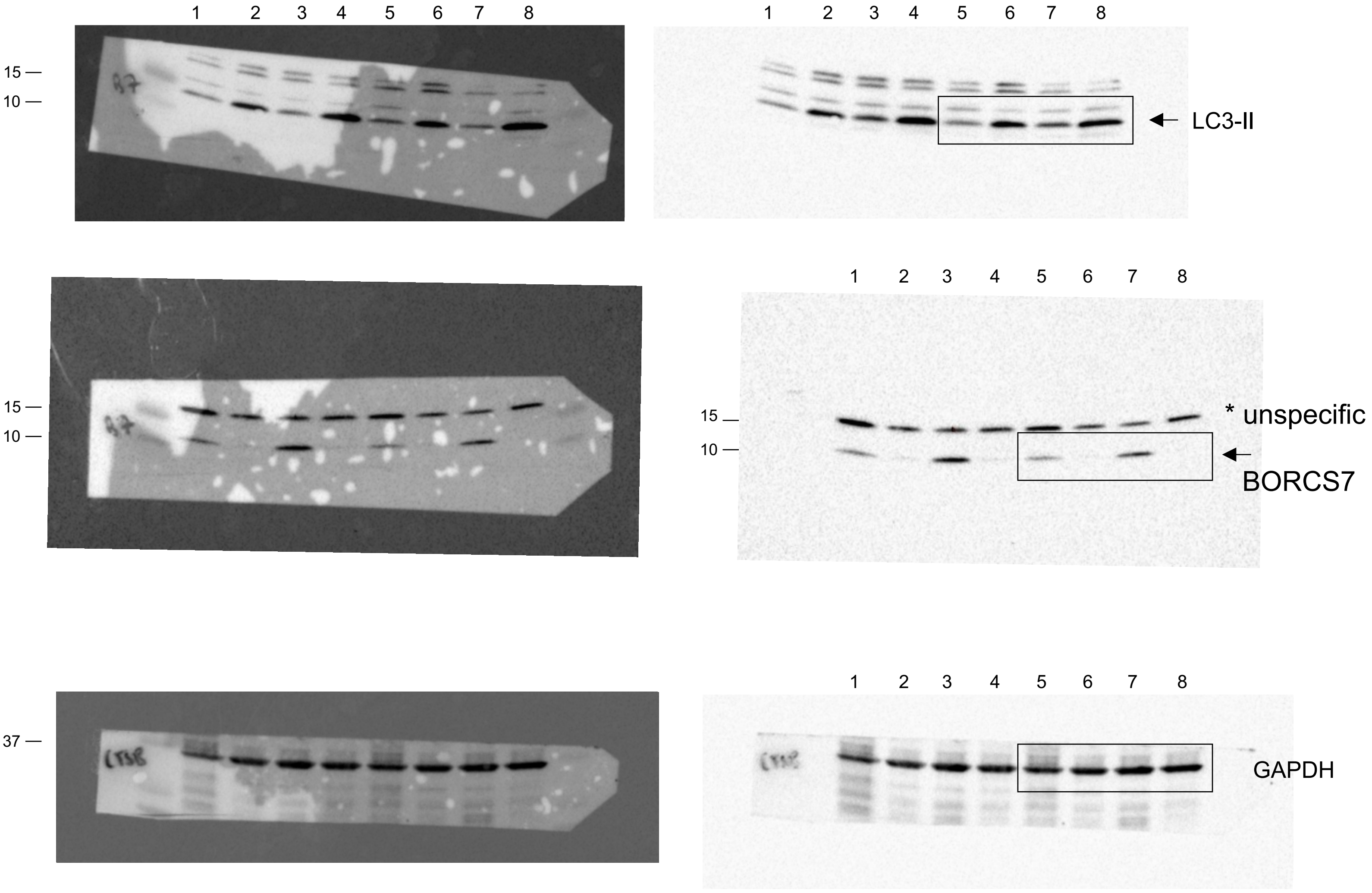

Supplement: Unedited blot and gel images [file jci-136-195336-s099.pdf]
